# A Genomic Survey of Positive Selection in *Burkholderia pseudomallei* Provides Insights into the Evolution of Accidental Virulence

**DOI:** 10.1371/journal.ppat.1000845

**Published:** 2010-04-01

**Authors:** Tannistha Nandi, Catherine Ong, Arvind Pratap Singh, Justin Boddey, Timothy Atkins, Mitali Sarkar-Tyson, Angela E. Essex-Lopresti, Hui Hoon Chua, Talima Pearson, Jason F. Kreisberg, Christina Nilsson, Pramila Ariyaratne, Catherine Ronning, Liliana Losada, Yijun Ruan, Wing-Kin Sung, Donald Woods, Richard W. Titball, Ifor Beacham, Ian Peak, Paul Keim, William C. Nierman, Patrick Tan

**Affiliations:** 1 Genome Institute of Singapore, Singapore, Republic of Singapore; 2 Defense Medical and Environmental Research Institute, DSO National Laboratories, Singapore, Republic of Singapore; 3 Institute for Glycomics, Griffith University (Gold Coast Campus), Southport, Queensland, Australia; 4 Defence Science and Technology Laboratory, Porton Down, Salisbury, United Kingdom; 5 Microbial Genetics and Genomics, Northern Arizona University, Flagstaff, Arizona, United States of America; 6 J. Craig Venter Institute, Rockville, Maryland, United States of America; 7 Faculty of Medicine, University of Calgary Health Sciences Centre, Calgary, Alberta, Canada; 8 School of Biosciences, University of Exeter, Exeter, United Kingdom; 9 Duke-NUS Graduate Medical School Singapore, Singapore, Republic of Singapore; University of Toronto, Canada

## Abstract

Certain environmental microorganisms can cause severe human infections, even in the absence of an obvious requirement for transition through an animal host for replication (“accidental virulence”). To understand this process, we compared eleven isolate genomes of *Burkholderia pseudomallei* (Bp), a tropical soil microbe and causative agent of the human and animal disease melioidosis. We found evidence for the existence of several new genes in the Bp reference genome, identifying 282 novel genes supported by at least two independent lines of supporting evidence (mRNA transcripts, database homologs, and presence of ribosomal binding sites) and 81 novel genes supported by all three lines. Within the Bp core genome, 211 genes exhibited significant levels of positive selection (4.5%), distributed across many cellular pathways including carbohydrate and secondary metabolism. Functional experiments revealed that certain positively selected genes might enhance mammalian virulence by interacting with host cellular pathways or utilizing host nutrients. Evolutionary modifications improving Bp environmental fitness may thus have indirectly facilitated the ability of Bp to colonize and survive in mammalian hosts. These findings improve our understanding of the pathogenesis of melioidosis, and establish Bp as a model system for studying the genetics of accidental virulence.

## Author Summary

With recent advances in genomics now permitting the systematic comparison of dozens, if not hundreds, of closely related bacterial strains, the opportunity arises for developing novel approaches to identify the complete repertoire of molecular factors governing interactions between hosts and pathogens. We explored these approaches using the model system *Burkholderia pseudomallei* (Bp), a Gram-negative bacterium that causes the tropical disease melioidosis. At 7.2 Mb, the Bp genome represents one of the most complex bacterial genomes sequenced to date. In this study, we present the first nucleotide-resolution comparative analysis of a panel of sequenced Bp strains. We identified a novel panel of genes demonstrating “positive selection”, referring to functional adaptations related to survival in soil, the natural reservoir of Bp. We propose a model and provide functional evidence that some of these genes may also have indirectly facilitated the ability of Bp to colonize and infect a mammalian host.

## Introduction

*Burkholderia pseudomallei* (Bp), the causative agent of the often-fatal disease melioidosis, represents one of the most complex bacterial genomes sequenced to date [1]. Comprising two circular chromosomes with a combined length of 7.2 Mb, the Bp genome contains an estimated ∼5800 genes involved in a myriad of functions, allowing microbial survival in extreme environments and virulence in diverse host species including humans, gorillas, pigs, and fish [2]–[3]. Epidemiological and genetic evidence suggests that Bp is likely an ‘accidental pathogen’, in that adaptations incurred by Bp in its natural environmental reservoir (soil) may have indirectly contributed to its ability to colonize a mammalian host [4]–[7]. Understanding the genetic basis of these environmental adaptations may thus provide important insights into the pathogenesis of melioidosis, and shed light on how environmental microorganisms are able to acquire novel traits enhancing their ability to cause opportunistic disease.

The evolutionary success of Bp as a thriving soil microbe suggests that most Bp strains are likely to possess a common repertoire of genes (the Bp core genome, or BpCG) regulating survival and fitness in this highly competitive environmental niche. Specific selective pressures encountered in soil, such as evading phagocytosis by amoebae [8] or ingestion by nematodes [9] might further enhance Bp environmental fitness by inducing modifications in BpCG genes, and some of these modifications might also contribute indirectly to mammalian virulence. Indeed, many classical virulence genes such as adhesins, fimbrae, exopolysaccharides and Type III secretion (TTS) systems are part of the BpCG [7], suggesting a plausible link between the BpCG and mammalian pathogenicity. Currently, little is known regarding the extent of genetic variation in the Bp core genome (BpCG) and whether BpCG variations might underlie potential virulence phenotypes. In this study, we undertook a comprehensive qualitative and quantitative survey of the BpCG across a panel of eleven Bp genomes, comprising nine independently derived strains, and two related strain pairs isolated from human patients at primary infection and disease relapse. We found evidence for the presence of several new genes in the Bp genome, and discovered a sizeable degree of genetic variation in BpCG genes. We identified over two hundred BpCG genes with signatures of positive selection, likely reflecting the activity of multiple distinct environmental pressures. Finally, we provide experimental evidence that some of these positively selected genes may have indirectly contributed to Bp pathogenesis in mammals, by facilitating interactions with host cellular pathways or the use of host nutrients.

## Results

### Genome Sequencing and Annotation

We analyzed whole-genome sequences from eleven Bp strains, comprising ten clinical isolates from four countries (Australia, Thailand, Singapore, and Vietnam) and one soil isolate (S13) from Singapore. To achieve maximal genetic diversity, we elected to analyze all Bp strains regardless of their source of isolation (clinical or environmental). Notably, environmental Bp isolates have also been shown to exhibit high levels of virulence in animal models [10]. Among the clinical isolates, strain pairs 1106a–1106b and 1710a–1710b were isolated from the same patients during either primary infection or disease relapse (Table S1). Reflecting the genetic diversity in this panel, the Bp isolates belong to different multi-locus subtypes (MLST) with an overall MLST allele/subtype ratio of 2.67, markedly higher than the allele/subtype ratio of the general Bp population (0.43, as of Jan 2009). Ten genomes were sequenced by conventional Sanger based shotgun methods (coverage range 7.75x – 11.4x), while strain Bp 22 was sequenced using next-generation instrumentation (GS20-454, average read length 100 bp, 20× coverage) followed by *de novo* assembly using a custom 454 large-insert paired-end sequencing protocol (CN and YR, manuscript in prep). The genome sequences were uniformly annotated by a FGENESB gene prediction pipeline [11], and predicted protein-coding regions, tRNAs, rRNAs, and potential promoters, terminators and operons were identified. Predicted genes were comprehensively annotated against known proteins in the NR, COG, KEGG and STRING databases (details in Methods). All genomes revealed similar benchmark data such as genome size, GC content, and numbers of predicted genes (Table 1).

**Table 1:** Genome Statistics of Sequenced *B. pseudomallei* Strains.

Genomes	K96243		1655		Pasteur 52237		406e		S13		22	
Chromosome	I	II	I	II	I	II	I	II	I	II	I	II
Genome size	4074542	3173005	4001239	2982333	4128191	3168620	4058126	3211140	4192562	3117285	3937887	3090538
# Predicted ORFs	3713	2619	3601	2524	3771	2612	3716	2657	3770	2594	3652	2636
Total predicted ORFs	*6332*		6125		6383		6373		6364		6288	
# Operons	774	475	784	471	801	483	794	502	802	472	776	520
Genome GC%	67.71	68.49	67.92	68.11	67.7	68.4	67.72	68.25	67.76	68.55	67.78	68.32
CDS length	924	1026	936.009	996.161	919.881	1022.21	918	1016	937.716	1017.72	885	949
# tRNA	53	7	43	5	50	5	50	7	47	5	52	7
# rRNA	9	3	7	1	5	2	8	3	5	2	9	3

Genomes	668		1106a		1106b		1710a		1710b		
Chromosome	I	II	I	II	I	II	I	II	I	II	
Genome size	3912947	3127456	3988455	3100794	3976828	3117768	4118877	3175883	4126292	3181762	
# Predicted ORFs	3460	2688	3560	2551	3553	2576	3748	2622	3733	2603	
Total predicted ORFs	6148		6111		6129		6370		6336		
# Operons	737	488	759	458	761	473	796	469	793	472	
Genome GC%	68.02	68.63	67.96	68.64	67.97	68.63	67.59	68.41	67.61	68.46	
CDS length	949	990	949	1029	950	1020	926	1019	923	1035	
# tRNA	52	7	52	7	47*	7*	50	5	53*	7*	
# rRNA	9	3	9	3	6*	6*	8	2	9*	3*	

*Differences in tRNA and rRNA numbers between primary and relapsed pairs arise due to differences in genome sequence coverage.

### Chromosomal Organization

**Figure 1:**
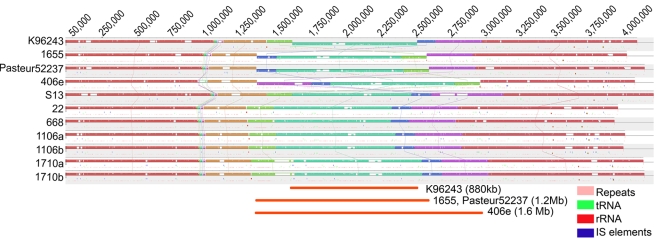
Genome Alignment of Bp Chromosome 1 Across Strains. Each strain chromosome is depicted as a series of ordered LCBs (Locally Collinear Blocks) with the putative origin of replication indicated by a black rectangle on the left side of each alignment. Vertical lines connect homologous LCBs across the genomes. LCBs identically present in the eleven genomes are given the same colors and horizontally flipped LCBs identify chromosomal inversions. Genomic locations of tandem repeats [12] (pink), rRNAs (red), tRNAs (green) and IS elements (blue) are depicted as short vertical lines below the LCBs. IS elements were identified using the ISfinder database [13]. Gaps or white spaces in LCB order represent strain-specific regions. Three large-scale inversions (dark orange) occurring in 4 strains are shown at the bottom of the alignment.

Both chromosomes (1 and 2) were highly syntenic across the Bp genomes (Figure 1 [12]–[13] and Figure S1). No evidence for inter-chromosomal exchange of genetic material across the two chromosomes was observed. We identified three large-scale inversions of 1.6 Mb, 1.2 Mb and 880 Kb on Chromosome 1, largely flanked either by rRNAs, tRNAs, or inverted protein units (Text S1). The 1.2 Mb inversion was observed in two strains, 1655 and Pasteur 52237, hailing from distinct geographic origins (Australia and Vietnam) and belonging to unrelated MLSTs, suggesting that this rearrangement may have independently occurred at least twice during Bp genome evolution. The other two inversions were only observed in single strains (406e and K96243), however it is worth noting that K96243 represents the original Bp reference genome described in 2004 [1].

### An Updated Bp Annotation Reveals Additional Genomic Complexity

**Figure 2:**
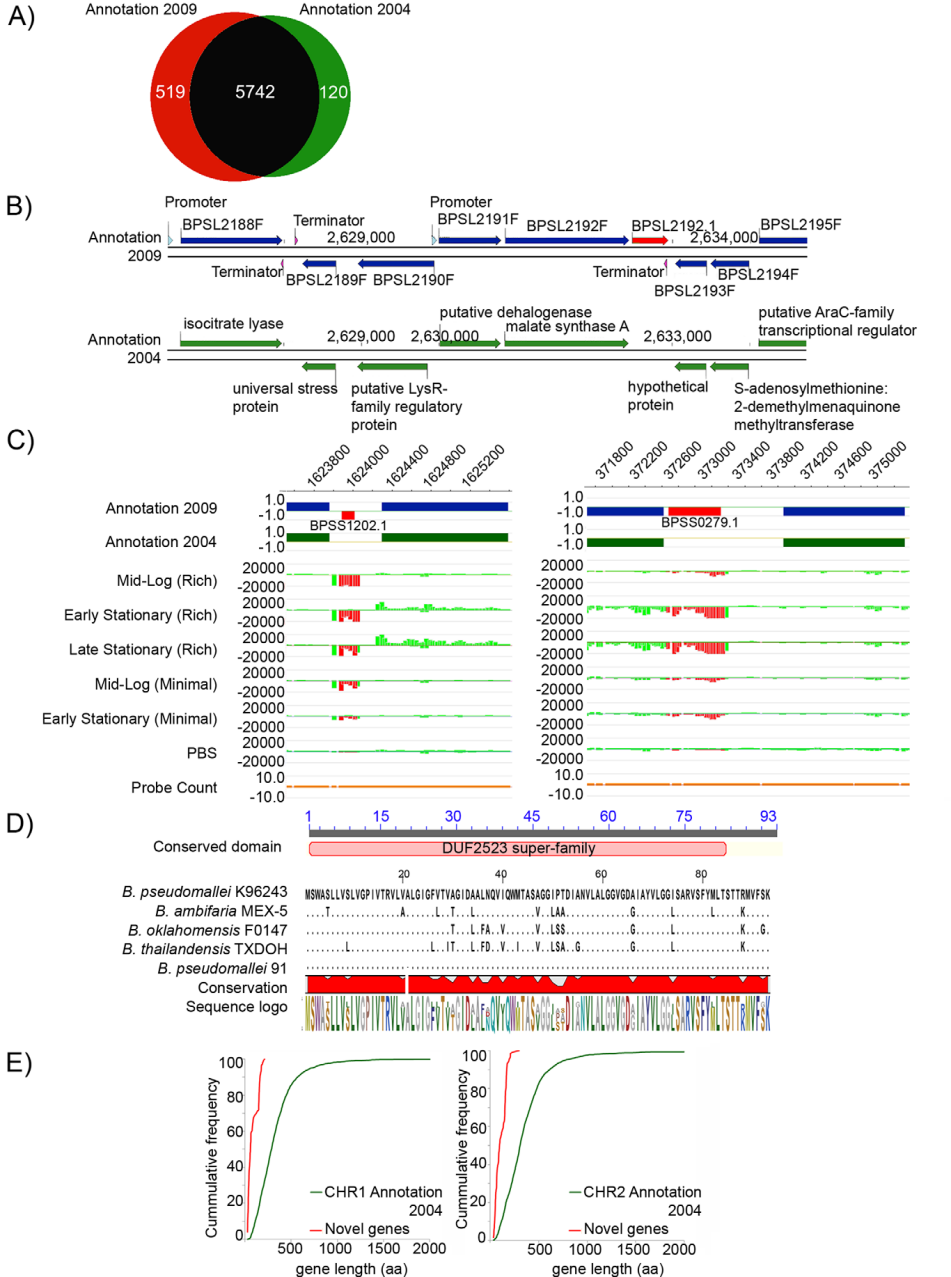
Experimental Re-annotation of the Bp Genome. A) Venn diagram showing the number of predicted genes either shared (black) or uniquely found in either the 2009 Bp annotation (red) or the original 2004 K96243 annotation (green). B) Bp K96243 genomic tracks showing novel genes. Row 1: Genomic locations of 9 Bp genes on Chr1 on both the positive (+) and the negative (−) strand in the 2009 genome annotation including a novel gene *BPSL2192.1* (red arrow) on the positive (+) strand. Row 2: Genomic locations of 8 Bp genes on the same region of Chr 1 in the 2004 genome annotation. C) mRNA transcripts associated with novel genes. (left) Row 1: Genomic locations of 5 Chr 2 Bp genes (blue bars) and 1 novel gene (*BPSS1202.1*: red bar) in the 2009 annotation. The novel gene lies on the negative (−) strand. Row 2: Genomic locations of the same 5 Chr 2 genes on Chr 2 (green bars) in the 2004 annotation. Rows 3–7: Transcript expression under six growth conditions. *BPSS1202.1* is expressed in all conditions except early stationary phase in minimal media. Row 8: Probe coverage associated with these genes. (right) A second example of a novel gene (*BPSS0279.1*) expressed primarily in both early and late stationary phase in rich media. D) Evolutionary conservation of a novel gene. Cross species comparison by BLAST of a predicted novel gene (*BPSL3348.1*) across five *Burkholderia* species. Multiple sequence alignments were generated using ClustalX [21]. Organism names are indicated at the beginning of the alignment. Identical residues are indicated as black dots. The conserved domain (identified by CDD search) is shown at the top of the sequence alignment in pink. The red block below the alignment indicates the level of conservation. The sequence logo of the alignment is shown at the bottom. E) Comparison of gene length between the 282 novel genes supported by two lines of evidence (red), and all 2004 genes (green) for chromosomes 1 and 2. The graph plots cumulative gene frequency against gene length in amino acids.

Our comparative analysis allowed us to revisit the original 2004 genome analysis with updated annotation protocols. Our annotation pipeline identified 6332 protein coding genes in Bp K96243 (Datasets S1 and S2), a considerably higher number (∼10%) than the 5855 genes originally described [1]. The vast majority (90%) of genes, however, were commonly identified in both annotation pipelines (Figure 2A), indicating that differences in the two annotation sets are likely due to subtle differences in the prediction algorithms used [14]–[15] (FGENESB vs GeneMark/Glimmer). Deciding to investigate these previously unreported genes, we sought to distinguish between likely *bona-fide* new genes and those arising due to computational over-prediction (false positives). We manually curated a set of 519 novel predicted genes exhibiting non-overlapping start-stop boundaries to the previously reported genes (see Figure 2B for an example), and subjected the 519 putative novel genes to three independent lines of analysis (mRNA transcript information, homology to previously reported genes, and presence of ribosomal binding sites, RBSs).

First, using whole genome tiling microarrays covering the entire non-repetitive Bp K96243 genome, we identified transcription units from Bp cultures isolated from six distinct growth conditions (see Methods, [16]). Confirming the accuracy of the microarray, many mRNA transcripts were tightly associated with the boundaries of previously-identified genes (Figure S2). Of the 519 novel genes, we found that 280 (53%) were associated with discrete mRNA transcripts. 178 novel genes exhibited mRNA transcripts in at least 1 out of 6 different growth conditions, indicating that they are differentially-regulated (Figure 2C), while the remaining 102 were constitutively expressed across the six conditions. The presence of several novel gene transcripts was also directly confirmed by targeted RT-PCR assays (Figure S3). To investigate if any of the novel genes might correspond to non-coding RNAs (ncRNAs), we used Rfam, a public database of non-coding RNA families [17], to identify ncRNAs in the BpK96243 reference genome. Of 82 small ncRNAs identified by Rfam analysis, 8 ncRNAs corresponded to the novel genes.

Second, using matching criteria similar to other studies [18]–[19] (see Methods, [20]), approximately 46% of the novel genes (239) were associated with at least one other matching protein in the COG, KEGG, STRING and NR databases (Figure 2D, [21]). 138 novel genes had matching proteins previously observed in other Bp strains, and 97 novel genes had matches to other *Burkholderia* species. A small fraction (∼1%) exhibited homology to other non-*Burkholderia* species (eg *Xanthomonas oryzae* pv. oryzae MAFF, *Sodalis glossinidius* str morsitans).

Third, using the RBSfinder program [22]–[24], we checked the novel genes for the presence of ribosome binding sites (RBS). The ability of RBSfinder to detect true RBSs in the Bp genome was confirmed by benchmarking the numbers of RBS predictions using previously-identified Bp genes against a set of background randomized sequences [25]–[26] (Text S2). Of the 519 novel genes, we identified high-confidence RBSs in 309 genes (59.5%), without requiring alteration of the predicted gene start/stop coordinates.

Combining these three lines of supporting evidence (mRNA transcripts, database matches, presence of RBS), we identified 282 novel genes supported by two lines of evidence (“dual evidence genes”), and 81 novel genes supported by all three lines (Table S2). A comparison of compositional features (length, G+C content, CAI, hydrophobicity [27]) between the 282 dual evidence genes and 5728 protein-coding genes from the original 2004 annotation revealed striking differences in gene length between the sets (average gene length 98±56 aa vs 348±307 aa between novel and 2004 genes, p = 1.23×10^−304^) (Figure 2E). Significant differences in G+C content, CAI, and hydrophobicity were also observed (eg G+C content 0.63±0.1 vs 0.68±0.05, p = 9.69×10^−17^) (Table S3). Interestingly, some of these latter compositional differences might be indirectly related due to the short lengths of the novel genes, as significant G+C content, CAI, and hydrophobicity differences were also observed when a set of “short length” genes from the original annotation (<200 aa) were compared against the entire 5728 set (Table S3). Because compositional differences can often influence gene prediction accuracy [28]–[29], it is possible that some of these differences might have contributed to the novel genes being missed in the original annotation. To facilitate integration with existing genome features, we assigned identities to the 282 novel genes based on their proximity to existing genes (eg *BPSL2192.1*) (Table S2).

We also investigated the 120 genes missed in the current gene prediction analysis but identified by the previous 2004 genome annotation (Table S4). Of these 120 genes, 87 genes (73%) were categorized either as “doubtful CDs”, “gene remnants”, or “pseudogenes” in the original 2004 annotation, indicating that these genes were likely regarded as ambiguous in the previous annotation as well. Of the remaining 33 genes, 21 genes encode hypothetical proteins while another 6 appear to have bacteriophage origins that may contain coding signals distinct from the rest of the Bp genome. The ambiguous nature for three-quarters of these genes, coupled with presence of atypical coding signals, provides the most likely explanation for their failure to be detected by the current automated prediction pipeline.

The availability of multiple Bp genomes also permitted the analysis of pseudogene dynamics within a species. Of 26 previously-described pseudo-genes in Bp K96243 [1], at least 6 were ‘resurrected’ in >6 other Bp genomes. For example, the *BPSL2828* pseudo-gene exhibits a premature truncation due to a stop codon at position 107 (TGG → TGA). This mutation, however, was only observed in Bp K96243 and Bp Pasteur 52237; while the other 9 Bp genomes had an extended gene sequence to position 147 (Figure S4). The differential presence of multiple pseudogenes across the Bp strains suggests that pseudogene formation in Bp is likely to be an active and highly dynamic process, consistent with its role as a recently evolved pathogen.

### Comparative Analysis of the Bp Core Genome

**Figure 3:**
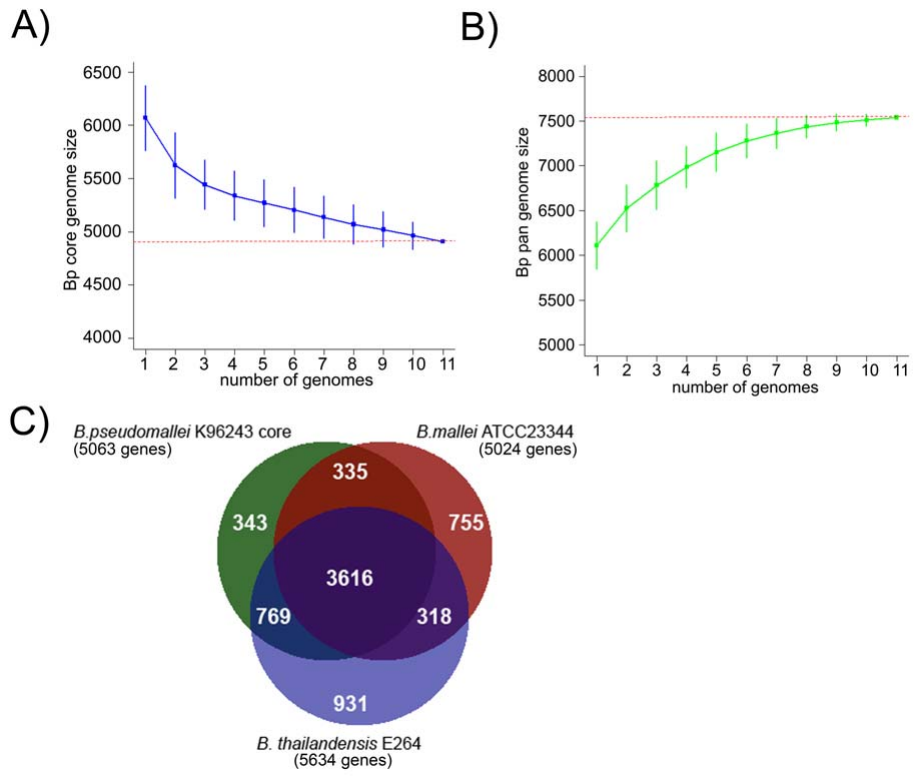
Comparative Analysis of the Bp Core Genome. A) Depletion curves for the Bp core genome (blue). Vertical bars represent standard deviation values based upon one hundred randomized input orders of the Bp genomes (http://www.rproject.org/, The R Project for Statistical Computing) [30]. B) Accumulation curves for the Bp pan genome (green). C) Distribution of orthologous genes between the Bp K96243 core genome (4908 core genes + K96243 paralogs  = 5063 genes), *B. mallei* ATCC23344 and *B. thailandensis* E264. The Venn diagram depicts the number of genes either shared or unique between one or more *Burkholderia* species. Figures in brackets indicate the total number of genes compared.

An analysis of gene orthologs across the Bp genomes identified a BpCG of 4908 genes present in all 11 strains (Figure 3A, [30]), with slight variations in individual genomes due to the presence of gene duplications and paralogs (range 5049–5139 genes). Similar core genome estimates were obtained when the analysis was confined to the nine independently derived isolates (Figure S5). We confirmed the robustness of this BpCG estimate using the method of Tettelin et al [31]. An evolutionary comparison of the BpCG against two closely related *Burkholderia* species with highly distinct niches - *B. mallei* ATCC23344 (Bm), a intracellular pathogen specific to horses [32], and *B. thailandensis* E264 (Bt), a non pathogenic, environmental bacterium [33]–[34], defined a common set of ∼3616 genes found in all three species (Figure 3C). 270 out of 335 genes are common to Bp and Bm with no orthologs in Bt, while 641 out of 769 genes are common to Bp and Bt with no ortholog in Bm. Besides the core genes, gene accumulation curves also project the global gene repertoire of Bp (the Bp pangenome) to be ∼7,500 genes (Figure 3B), a number close to 1.5x the size of the Bp core genome. A detailed analysis of the Bp pangenome will be described elsewhere.

### Genetic Variation in the Bp Core Genome

**Figure 4:**
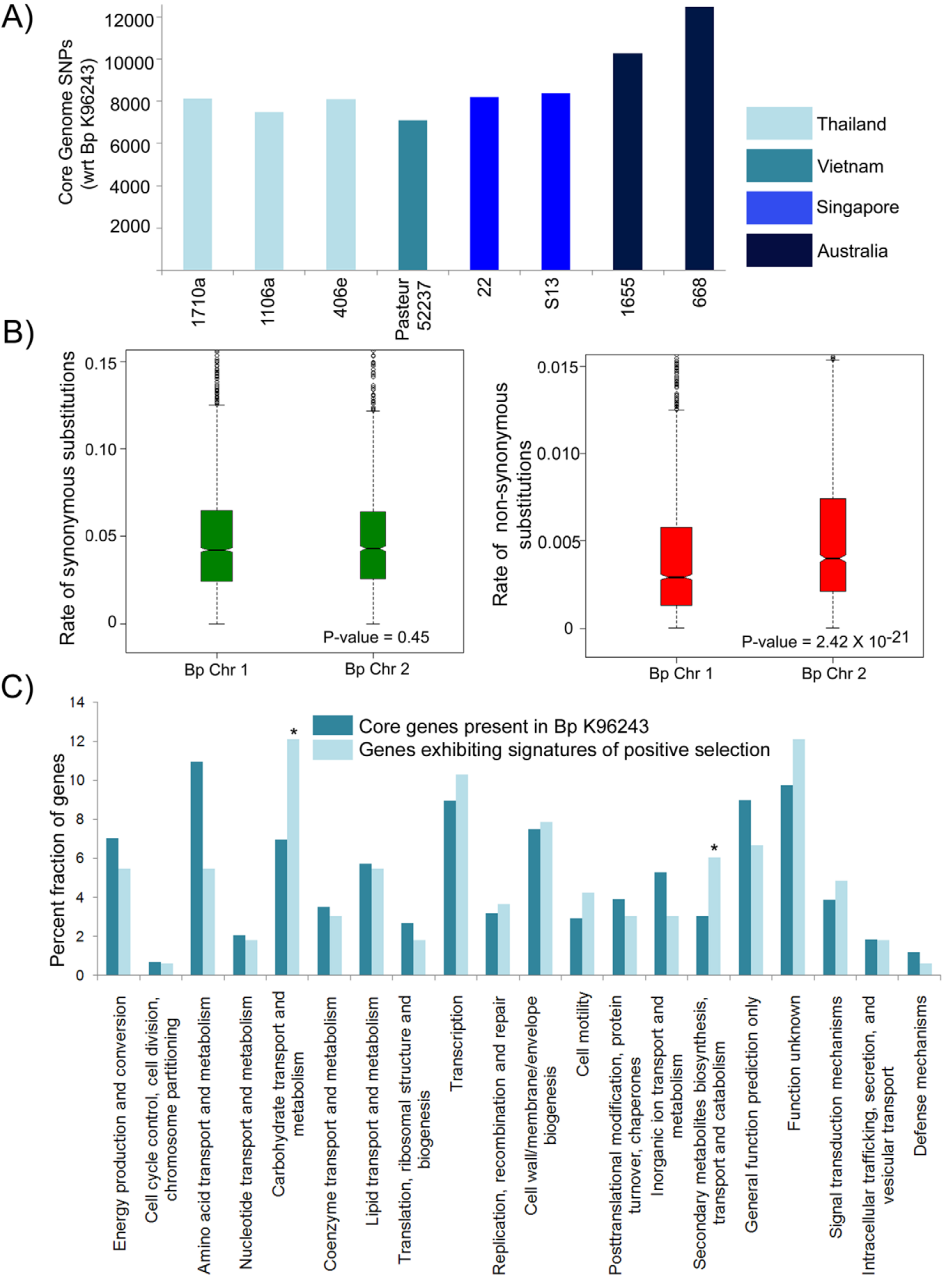
Genetic Variation in the Bp Core Genome. A) Distribution of SNPs across 11 Bp genomes. Core genome genes in all strains were compared against Bp K926243 to identify SNPs. For primary and relapse strain pairs (1106a/b and 1710a/b), only the primary strain is depicted. Geographical origins of the strains are depicted as different column colors. B) Chromosomal patterns of synonymous and nonsynonymous SNPs. Rates of synonymous (K_s_) and nonsynonymous (K_a_) substitution were estimated for Chromosomes 1 and 2 using a set of 4673 high-quality orthologous genes covering the 11 Bp strains. Each hourglass plot (interquartile range, IQR) represents the 25% to 75% range for that chromosome, with the bottleneck placed at the sample median. Horizontal tick marks show the range of all elements within Quartile 1–1.5 X IQR and Quartile 3+1.5 X IQR (equivalent to the 99.3% interval of a normal distribution). Open circles represent outliers (data points outside this range). The width of the bottleneck (i.e., the length of the V-shaped notch) is an indication of the confidence of the median; a lack of overlap of the bottleneck between samples implies that the samples are statistically different. Chromosomes differ significantly in K_a_ values (P = 2.42×10^−21^, t-test) but not in K_s_ values. C) Functional enrichments in the Bp core genome. COG functional categories are indicated on the x axis, and the percentage of genes in each COG category is shown on the y axis. Dark blue bars represent Bp core genes. Light blue bars indicate genes under positive selection in Bp strains. COG categories that are significantly enriched, (P<0.05, binomial test; bonferoni correction applied) in positively selected genes relative to the core genes are indicated by an asterisk.

To survey the landscape of genetic variation in Bp, we focused on a high quality ortholog set of 4673 BpCG genes (one orthologous gene per genome with >50% sequence similarity, each member exhibiting positional conservation to every other member, and excluding paralogs). We catalogued single-nucleotide polymorphisms (SNPs) and insertion/deletion sequences (indels) in the BpCG. Each Bp strain exhibited an average of ∼8594 SNPs compared to the K96243 reference genome, resulting in an overall SNP/Kb frequency of ∼2.0 for BpCG genes, while indels account for 0.1% and 0.3% of the total genetic variation in chromosomes 1 and 2 respectively. We confirmed the reliability of the genetic variation data by several methods. First, we confirmed by targeted resequencing >100 randomly-selected SNPs and 25 randomly-selected indels (data not shown). Second, 83% of identified SNPs are either (a) recurrently observed across multiple genomes (Table S5) [35], or (b) observed in Bp genomes of particularly high sequence quality (1106a, 1710b, 22, K96243 and 406e) (Table S5). Third, the SNP distributions are entirely consistent with geographic models in that strains with the highest levels of genetic variation compared to K96243 were observed in isolates from Australia, the most geographically distant locale (Figure 4A). This is consistent with previous proposals that strains from Australia are genetically distinct from their Asian counterparts [36] and form an ancestral population [35]. The existence of a deep genetic distinction between the South East Asian and Australian strains was further supported by phylogenetic analysis of 14,544 shared orthologous SNPs across 23 Bp genomes (including the genomes analyzed in this study), and also by an MLST population structure analysis involving >1800 Bp strains (647 sequence types) (Figure S6).

Among the clinical isolates, strain pairs 1106a–1106b and 1710a–1710b were isolated from the same patients during either primary infection or disease relapse, with intervening periods of approximately three years (Table S1). Surprisingly, a comparison of the primary and relapse strain genomes in both pairs failed to reveal a significant number of newly acquired mutations in relapsed strains (4 variants in 1106a vs 1106b, 6 variants in 1710a vs 1710b, none recurrent between both pairs) (Table S6). This lack of genetic variation between the primary and relapsed strains suggests that the former may have remained dormant in the human host during this intervening period, supporting the notion that that the Bp genome is likely to exhibit a high degree of stability during *in vivo* infection and persistence.

### Positive Selection in the Bp Core Genome

To assess the functional implications of BpCG variation, we divided the BpCG SNPs into subsets predicted to cause either synonymous (K_s_) or nonsynonymous (K_a_) nucleotide substitutions. The K_s_ rate was similar between Bp Chr 1 and 2, indicating comparable levels of background genetic diversity between the two chromosomes. However, the K_a_ rate of Chr 2 was significantly higher than Chr 1 (P = 2.42×10^−21^, unpaired t-test, under a one-ratio model (M0) assuming a constant K_a_/K_s_ ratio, Figure 4B), indicating that BpCG genes on Chr 2 are experiencing a higher degree of functional substitution than Chr 1. These chromosomal differences support the model of Holden et al [1] that Chr 1 of Bp represents the ancestral chromosome, with genes primarily related to housekeeping functions while Chr 2 contains genes involved in accessory functions and secondary adaptation.

We identified BpCG genes with signatures of positive selection using established methods [37]–[39] (Figure S7 and Methods, [40]). A maximum likelihood analyses was performed on each Bp core gene to detect coding sequence sites displaying features of differential selective pressure (positive selection) using two different likelihood ratio (LR) models (M1a-M2a, or M7-M8). Out of 4673 genes, Model M1a-M2a was significant for 212 genes, while model M7 -M8 test was significant for 239 genes (K_a_/K_s_>1; ∼2% FDR; P<0.001, LR Test). In total, 211 genes were commonly identified by both models as being positively selected (Table S7). Consistent with these 211 genes exhibiting above-background rates of functional variation (median K_a_/K_s_ = 60.07 and P<0.001, LR Test), the average K_s_ value of the 211 positively selected genes was similar to the K_s_ value of non-PS genes (K_s_ = 0.2 for PS and non-PS genes, p = 0.56), while in contrast, K_a_, the rate of non-synonymous substitution was 3 times greater in the positively-selected genes compared to genes under neutral selection (p = 0.5×10^−5^, t-test). The K_a_/K_s_ value of the positively selected genes was also markedly higher compared to seven housekeeping genes typically used in MLST analysis (*ace*, *gltB*, *gmhD*, *lepA*, *lipA*, *narK* and *ndh*) (P<0.001, LR Test). A significantly greater fraction of positively-selected genes were identified on Chr 2 than Chr 1 (P = 0.006, χ^2^ test, 10000 simulations). These observations suggest that a significant proportion of the Bp core genome (∼4.5%) may be under positive selection.

We investigated whether the elevated K_a_/K_s_ rate of the 211 positively selected genes might be due to mutation or recombination between the genomes in this strain panel. All 4673 core genome alignments were tested for the potential presence of recombination using two different methods (GENECONV [41], and the Pairwise Homoplasy Index (Phi)) [42]. Combining both methods, 56 out of 4673 core genes were identified as exhibiting a recombination signature. Of these 56, only 3 belong to the 211 positively selected genes, indicating that only a relatively minor component of the 211 genes are associated with a recombination signature. We also assessed rho/theta, the recombination/mutation ratio, of the Bp genomes analyzed in this study [43]. Using the Clonalframe algorithm [43], an inspection of 4294032 variation sites estimated rho/theta to be 0.012–0.015 (95% credibility region) for Chr 1 and 0.015–0.019 for Chr 2 respectively. This low value suggests that mutation rather than recombination appears to be the predominant evolutionary process explaining the patterns of genetic variation observed in the current panel of Bp strains.

Consistent with the BpCG responding to multiple selective pressures, the positively selected genes were widely dispersed across a wide variety of functions, including metabolic processes, membrane functions, signal transduction, and gene expression regulation (Table 2). A functional category analysis subsequently revealed that positively selected genes in the Bp core genome were significantly enriched in COG categories related to secondary metabolism (P = 0.036) and carbohydrate metabolism (P = 0.01, binomial test after correction for multiple hypotheses) (Figure 4C), highlighting these two metabolic pathways as major processes experiencing selective pressure.

**Table 2:** Representative Bp Genes Exhibiting Signatures of Positive Selection.

Gene	P value	K_a_/K_s_	COG	Annotation
BPSL0321	2.01×10^−4^	6.77	G	N-acyl-D-glucosamine 2-epimerase
BPSL0709	1.38×10^−7^	116.83	K	Transcriptional regulator
BPSL0719	1.25×10^−5^	140.56	M	Membrane carboxypeptidase (penicillin-binding protein)
BPSL0837	2.34×10^−6^	17.35	G	Arabinose efflux permease
BPSL1057F1	8.38×10^−4^	62.27	-	Hypothetical protein
BPSL2084	2.90×10^−4^	23.87	Q	O-Methyltransferase involved in polyketide biosynthesis
BPSL1628	2.21×10^−6^	10.86	N	P pilus assembly protein, porin PapC
BPSL2015	7.13×10^−7^	36.39	G	Beta-glucosidase-related glycosidases
BPSL2770	8.39×10^−11^	175.40	M	Predicted sugar phosphate isomerase involved in capsule formation
BPSL3029	8.67×10^−8^	33.88	M	UDP-N-acetylmuramyl pentapeptide synthase
BPSS0117	6.13×10^−6^	32.67	T	Signal transduction histidine kinase
BPSS0151	7.59×10^−4^	27.17	I	Fatty acid desaturase
BPSS0161	7.48×10^−4^	57.64	Q	Probable taurine catabolism dioxygenase
BPSS1403	3.56×10^−4^	14.20	N	Flagellar motor switch/type III secretory pathway protein
BPSS0415	2.11×10^−6^	8.25	-	Putative lipoprotein
BPSS0460	5.00×10^−4^	205.37	N	Methyl-accepting chemotaxis protein
BPSS0893	5.81×10^−12^	48.72	M	Outer membrane protein (porin)

*P* values are derived from likelihood ratio tests (Model M2a vs M1a, or M8 with M7). K_a_/K_s_ - Ratio of nonsynonymous (K_a_) to synonomous (K_s_) mutation rates. K_a_/K_s_ values of >1 indicate positive selection, with larger values indicating stronger selection. COG pathway codes are as follows: G, carbohydrate transport and metabolism; I, Lipid transport and metabolism; K, transcription; M, cell wall/membrane biogenesis; N, cell motility; Q, secondary metabolites biosynthesis, transport and catabolism; and T, signal transduction mechanisms; “-” indicates that no COG category was assigned.

### Positively Selected Genes May Contribute to Mammalian Virulence

**Figure 5:**
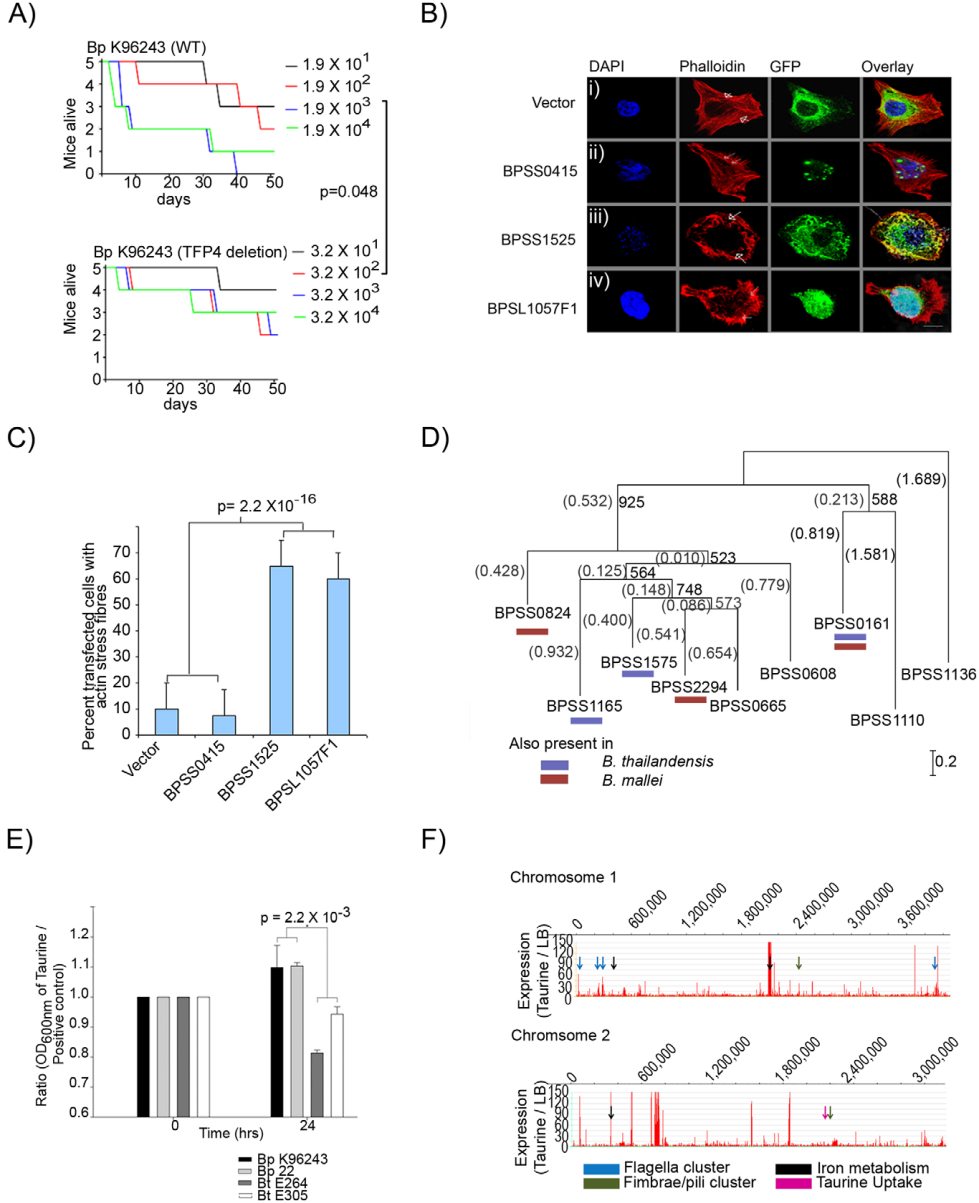
Functional Analysis of Positively Selected Genes. A) Relative Virulence of TFP4 Deletion Mutants: Graphs show survival curves of BALB/c mice following intranasal challenge with varying dosages of Bp (left – K96243 wild-type, right – TFP4 deletion strains). See Methods for infection assay details. The TFP4 deletion strain is significantly less virulent compared to Bp K96243 parental controls (p = 0.048, Mantel-Haenszel log rank test). Units in the color bar refer to Bp colony forming units (CFU). B) Transfection of HeLa cells using i) vector, ii) *BPSS0415*, iii) *BPSS1525* (*BopE*) and iv) *BPSL1057F1*. Cells were stained with rhodamine-phalloidin and DAPI to identify actin filaments and nuclei respectively. All genes were tagged with GFP at the N-terminus. Cells transfected with either empty vector or *BPSS0415* exhibited normal actin structures and filaments (arrowheads). Cells transfected with either *BopE* or *BPSL1057F1* exhibited dissolution of normal actin filaments with the presence of actin stress fibers (arrowheads); Bar 10 µm. C) Population analyses of transfected cells. Values were presented as percentage of the total number of transfected cells (n = 40), obtained from four independent experiments. D) Protein sequence relationships of nine taurine dioxygenase (*tauD*) genes from *Burkholderia pseudomallei* K96243. Bootstrapped Neighbor-Joining trees [52] were constructed using ClustalX [21], and drawn using NJplot [53]. Each branch was compared against 1000 resamplings of the alignment data. Bootstrap values and branch lengths are shown at the branch points, and distance units are shown in the lower right hand corners of the tree. The blue bar indicates the presence of a *tauD* homolog in *B. thailandensis*, red bar in *B. mallei*. *BPSS0665* is the *tauD* gene located in GI14. The *tauD* gene *BPSS0161* exhibits a signature of positive selection. E) Taurine utilization in Bp and Bt. Strains were grown in medium containing either taurine or free sulfate (Na_2_SO_4_-). To normalize differences in intrinsic growth rate, each strain was plotted as a ratio between its growth in taurine compared to free sulphate (y-axis) during 0 hrs or log-phase growth (x-axis). No growth was observed in taurine or Na_2_SO_4_-deficient media. Error bars represent the standard deviations between replicate cultures. F) Identification of taurine-regulated genes. Transcriptome profiles of taurine-exposed Bp were compared to Bp grown in laboratory rich media. Both populations were isolated at stationary phase. Rows represent Bp Chr 1 and Chr 2. Y-axes represent levels of transcriptional up-regulation of Bp genes in the presence of taurine relative to rich media (LB). Arrows depict gene clusters related to flagella (blue), iron metabolism (black), fimbrae/pili (green), and taurine metabolism (pink, *BPSS1572-1575*).

We were intrigued by the possibility that the positively selected genes, while overtly responding to environmental pressures encountered by Bp in soil, might indirectly facilitate the colonization of mammalian hosts. Supporting this notion, the positively selected genes were significantly enriched in genes previously identified as putative virulence-related genes [1] (20 genes, P = 0.019, based on 10,000 empirical permutations). For example, one representative class of virulence-related genes are Type IV pili (TFP), which are bacterial surface proteins implicated in multiple cellular processes, including motility, cell adhesion, microcolony formation, and virulence [44]. Of eight previously identified TFP loci in Bp K96243 [45], positively selected genes were associated with three TFP loci (TFP2, TFP4 and TFP7), with the TFP4 Type IVA minor pilin locus containing two positively selected genes (*BPSL2754* pilW and *BPSL2755* pilV). To evaluate if TFP4 might be involved in mammalian virulence, we generated isogenic Bp mutant strains deleted in the TFP4 locus, and tested the virulence of TFP4 deletion strains in a BALB/c mouse intranasal infection assay [46]. TFP4 deleted strains exhibited significantly reduced virulence compared to parental Bp K96243 wild-type controls (p = 0.048, Mantel-Haenszel log-rank test, Figure 5A), supporting a role for Type IV minor pilin activity in murine virulence. These results suggest that a subset of positively selected genes in Bp may influence virulence in mammals.

To further explore if other positively selected genes might conceivably provide traits facilitating successful mammalian infection, we then investigated two other features typically associated with successful intracellular human pathogens - a) the ability to interact with host cellular processes, and b) the ability to utilize host metabolites as nutrients. Previous studies have shown that many microbial pathogens can alter host cytoskeletons and cell morphology during infection, using proteins such as TTS factors to induce actin stress fibers, lamellipodia, and filapodia [46]–[48]. To examine the role of positive selection in this process, we curated a list of ten positively selected genes, either related to TTS biology (*BPSS1552*) or present in Bp and Bm (both pathogenic species) but absent from Bt (non-pathogenic) (Table S8). We cloned and expressed these ten genes in Hela cells, and examined the transfected cells for cytoskeletal perturbations. As a positive control, we also included *BopE* (*BPSS1525*), a TTS effector protein capable of inducing actin rearrangements [49]. Nine of the positively selected genes were successfully expressed in Hela cells but did not induce any significant differences in actin morphology compared to vector controls (eg *BPSS0415*, Figure 5B). In contrast, cells transfected with *BPSL1057F1*, a hypothetical protein and one of the novel genes identified in this study, exhibited a marked increase in actin stress fiber formation in the majority (60%) of transfected cells, with phenotypes very similar to *BopE* transfection (Figure 5B and 5C). Protein analysis of *BPSL1057F1* revealed the presence of a twin-arginine signal peptide sequence, often found in proteins exported into an extra-cellular environment [50]. These results suggest that some positively selected genes in Bp may provide Bp with the potential to interact with host cellular pathways.

We also analyzed the list of positively selected genes for potential genes involved in host metabolite catabolism. Of metabolites linked to the 10 positively selected secondary metabolism genes, we focused on taurine (2-aminoethanesulfonate), since taurine is an amino acid found at high levels in potential mammalian hosts in muscles, bile, and white blood cells, but absent or present at only trace levels in bacteria and plants [51]. Supporting the notion that Bp has developed an ability to metabolize taurine, the taurine dixoygenase gene *BPSS0161* (*tauD*) exhibited a significant degree of positive selection across the eleven Bp genomes (P<0.001, K_a_/K_s_ = 57.6, EC 1.14.11.17). Prompted by this finding, we further explored the role of taurine metabolism genes in Bp and discovered a previously-unreported species-specific expansion of additional *tauD* gene members in Bp. Specifically, compared to Bt or Bm which have three *tauD* genes on Chr 2, the Bp Chr 2 genomes harbor eight-nine *tauD* genes, a three-fold expansion (Figure 5D [52]–[53], also on Chr 2). The Bp *tauD* genes all share the same *tauD* pfam family domain (PF02668) but otherwise exhibit low sequence similarity between each other (average nucleotide homology of 36%), arguing against this expansion occurring by gene duplication. Instead, sequence analysis suggests that many of the Bp *tauD* genes were likely acquired by lateral gene transfer. For example, *BPSS0665*, another *tauD* gene, is localized to genomic island 14 (GI14), a region of codon bias deviation and atypical % GC content (Figure S8). Intriguingly, despite exhibiting many features of mobile elements, GI14 has been previously shown to be consistently present across a large panel of natural Bp isolates in contrast to other GIs [7] (Figure S8). It is possible that a selective requirement for maintaining levels of *tauD* activity might have contributed to GI14 behaving as a conserved feature of the Bp genome.

In other bacterial species, *tauD* is required to metabolize taurine as a sulphur source [54]–[55]. Experimental assays comparing the growth Bp and Bt strains confirmed that Bp also exhibits a significantly enhanced ability to efficiently utilize taurine as a sulphur source compared to Bt (p = 0.002, Figure 5E). The ability of Bp to metabolize taurine for sulphur utilization is specific, as Bp was unable to use taurine as an alternative carbon or nitrogen source, activities which are not mediated by *tauD* (Figure S8). Finally, to investigate the molecular response of Bp to taurine, we generated whole-genome transcriptome profiles of Bp exposed to high levels of taurine (250 uM). Here, the taurine concentrations used were based on previous reports studying taurine metabolism in *E. coli* [54]–[55]. Compared to Bp grown in standard laboratory media, taurine-exposed Bp exhibited transcriptional up-regulation of ∼280 genes, of which 40% (126 genes) have been previously associated with pathogenicity, host–cell interaction, or survival in diverse and challenging environments [1]. Specific examples of taurine-regulated genes implicated in virulence included several flagella gene clusters (*BPSL0024-BPSL0032*, *BPSL0224-BPSL0236*, *BPSL0266-BPSL0282*, *BPSL3288- BPSL3330*) [56], siderophore biosynthesis and iron metabolism genes (*BPSL1771- BPSL1787*, *BPSS0239- BPSS0244*, *BPSS0581- BPSS0588*) [57], and fimbrae/pili (*BPSL2026- BPSL2031*, *BPSS1593- BPSS1605*) [45] (Figure 5F, Table S9A and S9B). Taken collectively, these findings suggest that altered taurine metabolism likely mediated by *tauD* may represent a species-specific adaptation of Bp that may have also facilitated its ability to survive in infected mammalian hosts [58].

## Discussion

In this, the first nucleotide-scale comparative analysis of multiple Bp genomes, we expanded the known gene repertoire of Bp, defined the BpCG, and described the extent of genetic variation in BpCG genes. We identified a set of genes exhibiting positive selection, and examined how such variations can impact genomic organization and structure. Our results suggest that a significant proportion of the BpCG may be experiencing functional selection, and that a large aspect of this selection involves the modification of preexisting metabolic circuits related to carbohydrate and secondary metabolism. Importantly, we also provide evidence that a subset of these genes may have also facilitated the ability of Bp to interact with mammalian hosts, either structurally or nutritionally.

In our analysis, we have proposed that many of the genetic alterations observed in the positively selected genes were primarily driven by environmental pressures outside the human or mammalian host. Nevertheless, if Bp undergoes cryptic cycling through normal humans or other potential mammalian hosts, such as livestock or wild cattle [59], it remains possible that certain survival and virulence traits were directly selected for in mammals. In melioidosis-endemic NE Thailand, the majority of healthy individuals have antibodies to Bp by the age of 4 years, indicating constant exposure to the bacterium that may occur by inoculation, inhalation or ingestion [4]. Within such hosts, Bp might spend periods of time being exposed to the mammalian immune response and various physiologic traits. Subsequent return to the environment in a viable state, through skin desquamation or in urine and stool, could also lead to the selection of factors that promote survival *in vivo*. However, because we a) consider the mammalian host to be a relatively minor component of Bp ecology, b) such cryptic cycling through mammalian hosts has yet to be documented, and c) the lack of genetic variation between the primary and relapsed strains suggests that the Bp genome is likely to exhibit a high degree of stability during mammalian infection, we argue that this scenario is, on balance, possible but less likely.

A large proportion of Bp genes are still unannotated or poorly characterized, raising the need for systematic approaches to link discrete sets of Bp genes to their specific biological and cellular functions. The genomic identification of these positively selected genes should facilitate the process of targeted experimentation to elucidate the pathogenesis of melioidosis. The prioritization of candidate genes for targeted experimentation is particularly relevant for Bp due to its classification as a potential biothreat agent. Under international biosafety regulations, Bp research is typically conducted in high containment (Category 3) facilities and limited to highly focused projects [60] (http://www.selectagents.gov/). Finally, it is worth noting that the ability of this approach to uncover candidate host interaction genes and pathways from a genome as complex as Bp suggests that similar approaches should prove equally fruitful in elucidating novel aspects of biology in other recently emergent pathogens as well.

## Methods

### Ethics Statement

This research was approved by the Genome Institute of Singapore Institutional Review Board. All animal experimentation was conducted at DSTL (Defence Science and Technology Laboratory) in the United Kingdom (UK) under Animal (Scientific Procedures) Act 1986.

### Genome Annotations and Comparative Analysis

Bp genes were predicted using FGENESB [http://linux1.softberry.com/berry.phtml?topic=fgenesb&group=help&subgroup=gfindb (Softberry)]. tRNA genes were identified using tRNAScan-SE [20], and rRNA genes by sequence conservation (blastn, e-value threshold: 1e-08). Operons were identified based on a) distances between genes, b) likelihood of neighboring genes also appearing in other bacterial genomes as neighbors, and c) locations of predicted promoters and terminators. Genes were annotated against the NR, COG, KEGG and STRING [www.ncbi.nlm.nih.gov (NR); www.ncbi.nlm.nih.gov/COG (COG); www.genome.jp/kegg (KEGG); http://string.embl.de/ (STRING)] databases using the following criteria: i) BLASTP e-value threshold of <1e-10; ii) percent identify threshold of >60%, and iii) a percentage coverage threshold of 80%. These criteria were used based on previous studies [18]–[19]. Ribosome binding sites (RBSs) were identified using RBSfinder [22]–[24]. Notably, the consensus RBS sequences between *E. coli* and Bp are similar [25]–[26]. Non-coding RNAs were identified using the Rfam database [17]. CodonW (http://codonw.sourceforge.net/) was used to identify codon adaptation indexes (CAI), Kyte and Doolittle scales of hydrophobicity [27], GC percentages and gene lengths. Multiple whole-genome alignments were performed using Mauve 2.2.0 [61].

### Transcriptome Profiling

Bp K96243 cultures were isolated from six conditions: Luria-Bertani broth (mid-logarithmic, early stationary and late stationary phases, conditions 1–3), minimal media (mid-log and early stationary, conditions 4–5), or exposure to 1x PBS solution (condition 6). Bacterial mRNAs were profiled on a high-density Bp tiling array representing both strands of the Bp K96243 genome (7.2 Mb) (Nimblegen) (50-mers, 15-base overlap). All transcriptome profiles are the average of 2 biological replicates. Three distinct criteria were employed to consider a novel gene as “expressed”. First, an “expressed” novel gene was required to exhibit a minimum of 3 consecutive array probes with fluorescence intensities above the array median intensity. Second, for genes covered by more than five array probes, the combined pseudo-median expression value of the novel gene was assessed using the SIGN Test, a statistical method previously used to measure the transcriptional activity of genes using tiling microarrays [16]. Only novel genes passing the SIGN test were considered as “expressed” (p<0.05). Third, short novel genes covered by less than five probes that did not qualify for the SIGN Test were manually curated to confirm the presence of contiguous expression signals for each gene. For analyses of differential gene expression, ratios of normalized probe signals were computed. Probe identities with more than 2-fold up-regulation or down-regulation were matched to Bp gene identities. Genes that have 50% or more probes showing at least 2-fold up-regulation or down-regulation were taken as differentially expressed between the conditions compared.

### Bp Core Genome and Pan Genome

Gene orthologs across the Bp genomes were determined using OrthoMCL [62]. An all-against-all BLASTp [63] was performed, followed by a reciprocal BLAST to define putative ortholog pairs or recent paralogs (genes within the same genome that are reciprocally more similar to each other than any sequence from another genome). Reciprocal BLASTp values were converted to a normalized similarity matrix that was analyzed by the Markov Cluster algorithm MCL to define ortholog clusters. OrthoMCL was run with a BLAST e-value cut-off of 1e-5, and an inflation parameter of 1.5. The OrthoMCL output was used to construct tables of shared orthologs and strain-specific genes.

### Bp Core Genome Variation and Positive Selection

Orthologs exhibiting positional conservation across the Bp genomes were aligned at the DNA level with ClustalW [21] and manually confirmed. SNAP.pl was used to calculate the number of synonymous vs. non-synonymous base substitutions (Nei and Gojobori method) for all pairwise comparisons of ortholog sequences [40]. Ambiguous codons or codons with insertions were excluded from the tally of compared codons. Base-substitutions were also manually inspected to remove from consideration substitutions indirectly caused by upstream frame-shifts. GENECONV [41] was used to identify recombination breakpoints, and genes exhibiting a recombination signature were fragmented at the predicted breakpoints. The recombination sub-fragments (total 152 sub-fragments) were individually applied to the PHYLIP pipeline to infer maximum parsimony trees. The core gene alignments were also tested for the presence of recombination using the Pairwise Homoplasy Index (Phi) as implemented in the HYPHY package (100000 permutations, cutoff at ∼1% FDR) [42]. ClonalFrame version 1.1 was used to compute rho/theta, the recombination/mutation ratio [43]. Protein sequences were aligned using ClustalW (‘ktuple’ ⇒ 2 and ‘matrix’ ⇒ ‘BLOSUM’). PAL2NAL [64] Perl scripts were used to convert the multiple sequence protein alignments and corresponding DNA sequences into codon alignments. Maximum parsimony (MP) trees were generated using PHYLIP (‘dnapars’ module) using default values (http://evolution.genetics.washington.edu/phylip.html). Codon alignments and MP trees were analyzed by PAML 4.0 [38] to calculate K_a_/K_s_ (or ω) ratios and test different evolutionary models. The following nested models were used: M1a-M2a and M7-M8 [39]. A likelihood ratio test was used to compare model M2a with M1a, and model M8 with M7, at a significance cutoff of ∼2% FDR [38]. The nested model M0 (one-ratio)-M3 (discrete) was also used to confirm heterogeneity of K_a_/K_s_ in the cohort of positively selected genes [65].

### Construction of Isogenic Mutant Strains

Isogenic unmarked mutant Bp strains carrying a 3.7 kb deletion of the TFP4 gene cluster were generated as previously described in Boddey et al., 2006 [66]. Briefly, a TFP4 (*BPSL2749-BPSL2755*) targeting vector was constructed and conjugated into Bp K96243. Integrants were selected on chloramphenicol plates (100 ug/ml) and confirmed by PCR. Merodiploid integrants were then cultured without selection and plated onto medium lacking sodium chloride but containing 15% sucrose to enrich for colonies carrying a deleted chromosomal locus. Bp TFP4 mutants were confirmed both by PCR and Southern blotting.

### Mouse Virulence Studies

Virulence of wild-type and mutant Bp strains were assessed using an intranasal BALB/c mouse model as previously described [45]. Briefly, groups of six age-matched BALB/c female mice were anesthetized and infected intranasally with 10-fold dilutions (101–106) of either wild-type Bp K96243 or TFP5 deletion strains grown overnight at 37degC with shaking. Mice were recovered and survival was recorded for up to 51 days. The survival data was analyzed using the Mantel-Haenszel log rank test in GraphPad Prism 4 or by Regression with Life Data in MIniTAB v13.0, using a significance threshold of α = 0.05.

### Cell Culture, DNA Transfection and Immunoflouresence

Positively selected genes were PCR-amplified from Bp genomic DNA and subcloned into Vivid Colors®pcDNA® 6.2/N-EmGFP-GW/TOPO® mammalian expression vectors (Invitrogen). Hela cells were transfected using Gene Juice (Novagen), and cultured for 24 h after tranfection. Cells were fixed in 3.7% paraformaldehyde/PBS (pH 7.0). After washing and preincubation, cells were stained with Alexa Flour 555 phalloidin (Invitrogen) and DAPI (Sigma-Aldrich). Stained cells were visualized using a confocal Zeiss LSM 150 inverted laser scanning microscope and analyzed using Zeiss LSM Image Browser software (Carl Zeiss, Oberkochen, Germany).

### Taurine Utilization

2 Bp and 2 Bt strains (Bp K96243, Bp 22, Bt ATCC700388 and Bt E305) were cultured in modified M63 media, or media supplemented with 250 µM taurine or 250 µM Na_2_SO_4_. Cultures were grown at 37°C, 150 rpm and OD_600_ readings were taken every 2 hrs for 72 hrs. To study differential gene expression, Bp K96243 was cultured in modified M63 medium with 250 µM taurine at 37°C, 150 rpm for 48 hrs to reach stationary phase. The expression profile obtained was compared with that obtained for Bp K96243 cultured in LB at stationary phase. All transcriptome profiles are the average of 2 biological replicates.

## Supporting Information

Figure S1 Genome Alignment of Bp Chromosome 2 across Bp Strains. Each genome is depicted as a single LCB (Locally Collinear Block) with the putative origin of replication being indicated by a black rectangle (left side of each alignment). Gaps or white spaces within the LCBs represent strain-specific regions.
(0.20 MB PDF)

Figure S2 Bp transcript expression is associated with previously-identified genes. Top Row: Locations of 5 Bp genes on Chr 1 (green bars) and 6 Bp genes on Chr2 (green bars) on the positive (+) and negative (−) strands. All 10 genes are commonly found in both the 2004 and 2009 annotations. Bottom row: Transcript expression on both positive and negative strands as measured using tiling microarrays. Notice that the transcripts are tightly associated with the previously-identified genes. Red regions likely correspond to either 5′ or 3′ untranslated UTR regions.
(0.13 MB PDF)

Figure S3 Experimental PCR Validation of mRNA transcripts associated with novel genes. (A) mRNA transcripts detected by tiling microarrays associated with novel 2009 genes BPSL1301.1 and BPSL2337.1. Top Row: Locations of Bp genes on Chromosome 1 on positive (+) and negative (−) strands. Novel genes validated are shown in red. Bottom row: Transcript expression on both the positive and negative strands. (B) RT-PCR validation of novel gene transcripts. Lane 1: Blank/Negative control (water); Lane 2: positive control: 16S rRNA; Lane 3: Novel gene BPSL1301.1; Lane 4: Novel gene BPSL2337.1. The 100 bp molecular weight ladder is shown on the left.
(0.15 MB PDF)

Figure S4 Example of a differential pseudogene. Multiple sequence alignment of BPSL2828 identified as a pseudogene in BpK96243, against its homologs from other sequenced Bp genomes [a) gene sequence b) protein sequence]. Alignments were performed using ClustalW [6]–[7]. The Bp strain names are indicated at the beginning of the alignment. The black bar at the bottom of the alignment indicates the consensus. The mutation is encircled by a blue box.
(3.81 MB PDF)

Figure S5 Bp Core genome estimates from nine isolates. Depletion curves for the Bp core genome (blue: 11 Bp genomes; brown: nine genomes representing independently-derived strains). Vertical bars represent standard deviation values based upon one hundred randomized input orders of the Bp genomes [8]. The analysis revealed a highly similar BpCG gene set based on 9 isolates, comprising 4920 ORFs (compared to the 4908 ORFs based on the 11-isolate analysis).
(0.15 MB PDF)

Figure S6 Phylogenetic and MLST Analysis of Sequenced Bp Strains. To infer phylogenetic relationships between the sequenced Bp strains, we generated phylogenetic trees based on whole-genome shotgun sequencing data of 33 *Burkholderia* strains, including 23 Bp strains and 10 *B. mallei* strains as an outlier group. Consistent with Figure 4A in the Main text, the two Australian strains (668 and 1655) segregated in phylogenetic subbranches distinct from the South-East Asian strains (Figure S6A). This phylogenetic separation was further supported by a larger MLST-based population genetic analysis of 1827 isolates (647 sequence types), confirming the division of Bp into two major populations (Figure S6B). These results suggest that there are two major populations of Bp, an Australian and a Southeast Asian population [5], and that the Australian population may be more ancient and more diverse than the Southeast Asian population. A) Phylogenetic relationships of Bp isolates used in this study compared to other *Burkholderia* isolates with whole genome sequences. This phylogeny contains 33 genomes of Bp and Bm and is based on 14,544 shared orthologous SNPs [5]. Genomes used in this study are shown in red. B) Estimated population structure of Bp and *B. mallei* using allele frequencies of MLST data. Each thin vertical line represents a sequence type that is divided into two portions that resemble the proportion of 5,000 iterations where that sequence type was assigned to each of two populations. The red population is dominated by sequence types from Australia, while the black population is dominated by sequence types of Southeast Asian origin. Geographic affiliations of sequence types are labeled below the figure. Isolates whose genomes were used in this study are indicated along with the percentage of iterations that assigned them to each population. Data used was downloaded from http://bpseudomallei.mlst.net/ on November 23rd, 2009. Isolates with no information on the country of origin were excluded, leaving 647 sequence types of Bp (n = 645) and *B. mallei* (n = 2). Structure 2.2 [9] was used to analyze these sequence types according to the methods described in Pearson et al. 2009[5].
(0.15 MB PDF)

Figure S7 Schematic of Positive Selection (PS) Analysis Workflow. Overview of the positive selection analysis scheme. Size of each dataset is indicated in parentheses. Programs used are indicated next to the arrows.
(0.12 MB PDF)

Figure S8 Expansion of *tauD* Taurine Dioxygenase Genes in Bp. A) Row 1: Genome organization of GI14 (BPSS0652-BPSS0666) and surrounding regions on *Burkholderia pseudomallei* K96243 chromosome 2. Row 2: Columns represent codon bias deviation (dark blue) and %GC bias (grey) respectively, using a six-gene sliding window. Values were obtained using PredictBias Server [10]. The location of GI14 is shown in red at the bottom, corresponding to a region of codon bias and atypical GC content. The *tauD* gene BPSS0665F is highlighted in red. B) Hardwiring of GI14 in the Bp genome. Presence and absence of all 16 GIs were assessed in a panel of 98 Bp isolates by aCGH [11]. Both GI7 and GI14 (marked in red) are present in all Bp strains. C) Utilization of taurine as the sole i) carbon source or ii) nitrogen source by Bp K96243 and Bt ATCC700388. Cultures with taurine as the sole carbon and nitrogen source showed comparable growth with the respective negative controls, which is significantly less than the respective positive controls. Error bars represent the standard deviations between replicate cultures.
(0.30 MB PDF)

Table S1 List of *B. pseudomallei* Strains
(0.08 MB PDF)

Table S2 Novel genes supported by two or three lines of evidence
(0.06 MB PDF)

Table S3 Compositional Features of Novel Predicted Genes and Short-Length Sanger Genes Compared to All Sanger Genes. *Sanger genes less than 200 aa were defined as “short length”. All p-values were determined using an unpaired two tailed t-test (unequal variance).
(0.06 MB PDF)

Table S4 Previously-predicted BpK96243 genes missed by the FGENESB pipeline
(0.11 MB PDF)

Table S5 Recurrent SNPs and SNPs identified in five high-quality Bp genomes. ^ξ^SNP observed in at least two of eleven Bp genomes. *SNPs observed across 23 Bp genomes after removal of paralogous and non-shared loci [5]. ^†^The five high sequence quality genomes are: *B. pseudomallei* K96243, *B. pseudomallei* 22, *B. pseudomallei* 1106a, *B. pseudomallei* 1710b, *B. pseudomallei* 668.
(0.04 MB PDF)

Table S6 Sequence Variations between Primary and Relapse Bp Strains. GeneID: Based on 1106a annotation; SNP a -> b: nucleotide changes; S/N: Synonymous vs Nonsynonymous alteration; BPCG+: Present in Bp core genome.
(0.07 MB PDF)

Table S7 List of Positively Selected Bp Genes (ranked by K_a_/K_s_). A) Chromosome 1. B) Chromosome 2. *Genes with a recombination signature.
(0.14 MB PDF)

Table S8 List of selected gene candidates for transfection
(0.06 MB PDF)

Table S9 A) List of taurine regulated genes in Bp K96243 Chr 1 (up regulated > = 2 fold). B) List of taurine regulated genes in Bp K96243 Chr 2 (up regulated > = 2 fold).
(0.09 MB PDF)

Dataset S1 GenBank file of Bp K96243 Chromosome 1
(8.10 MB TXT)

Dataset S2 GenBank file of Bp K96243 Chromosome 2
(6.22 MB TXT)

Text S1 Motifs at inversions
(0.06 MB PDF)

Text S2 Accuracy Estimate of RBSfinder on the *B. pseudomallei* genome
(0.11 MB PDF)
