# Supplementary figures and images for: A Genomic Survey of Positive Selection in Burkholderia pseudomallei Provides Insights into the Evolution of Accidental Virulence
Source: PLoS Pathog. 2010 Apr 1;6(4):e1000845. doi: 10.1371/journal.ppat.1000845 (PMC2848565; doi:10.1371/journal.ppat.1000845)

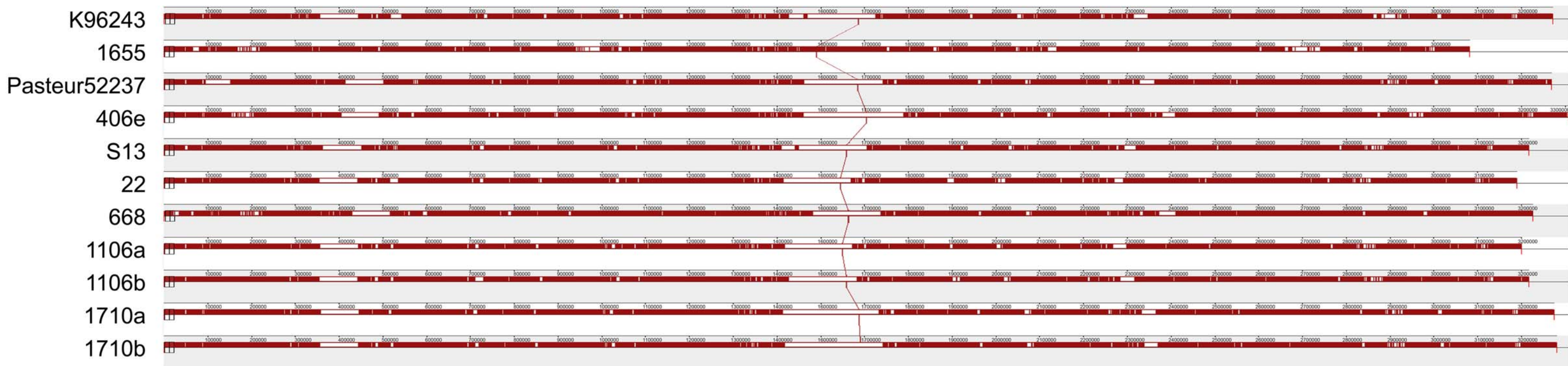

Supplement: Figure S1 — Genome Alignment of Bp Chromosome 2 across Bp Strains. Each genome is depicted as a single LCB (Locally Collinear Block) with the putative origin of replication being indicated by a black rectangle (left side of each alignment). Gaps or white spaces within the LCBs represent strain-specific regions. (0.20 MB PDF) [file ppat.1000845.s001.pdf]

A)

Chromosome 1: BPSL1301.1 (1516738-1516941)

Chromosome 1: BPSL2337.1 (2825017-2825109)

Genomic Location

Transcript  
Expression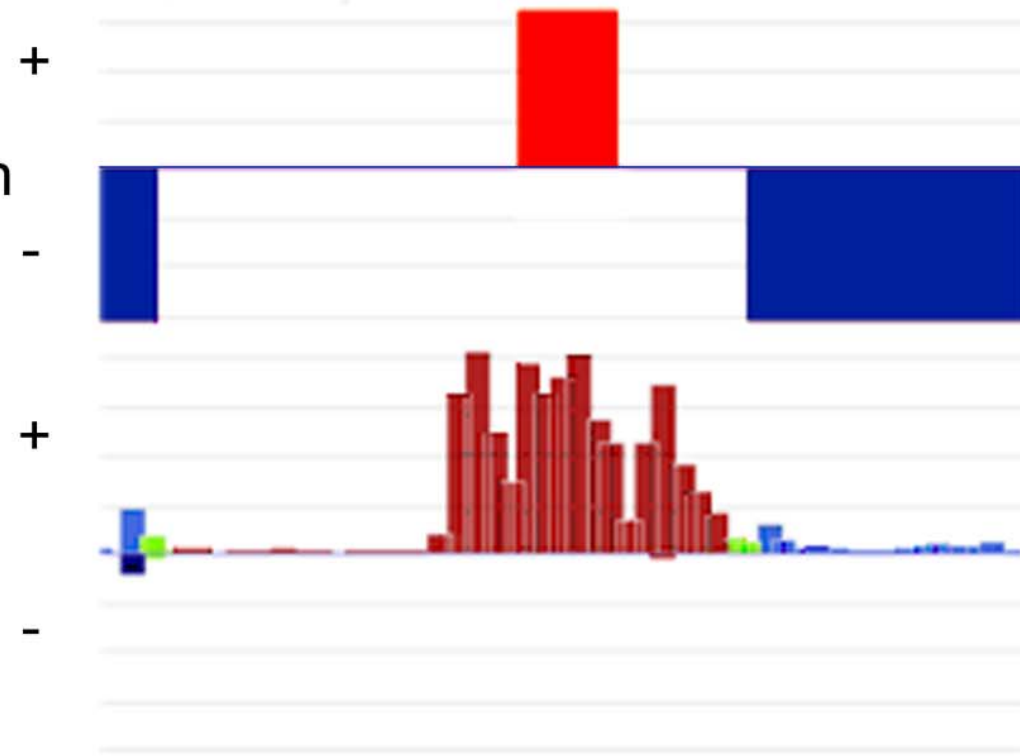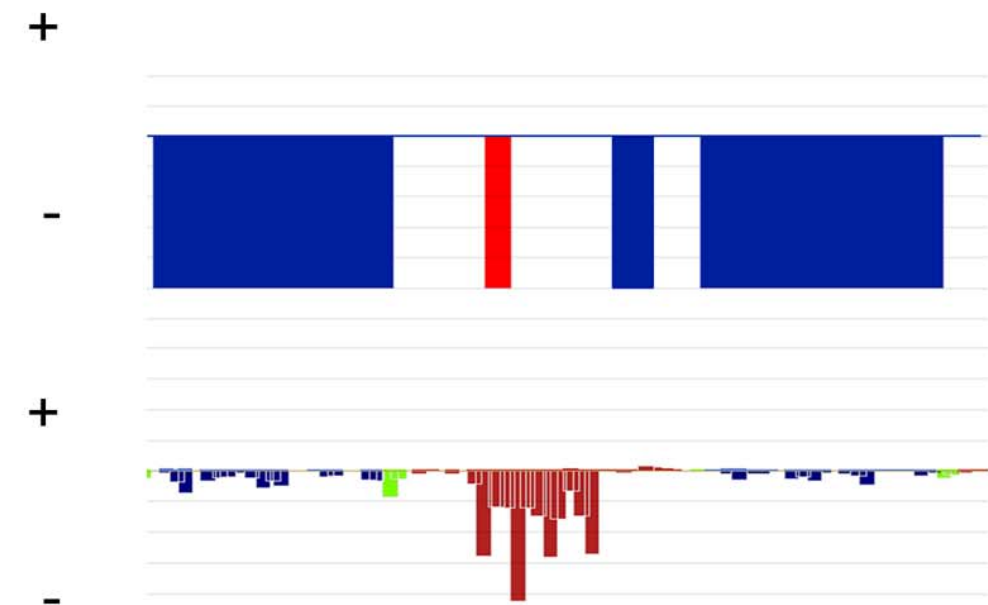

B)

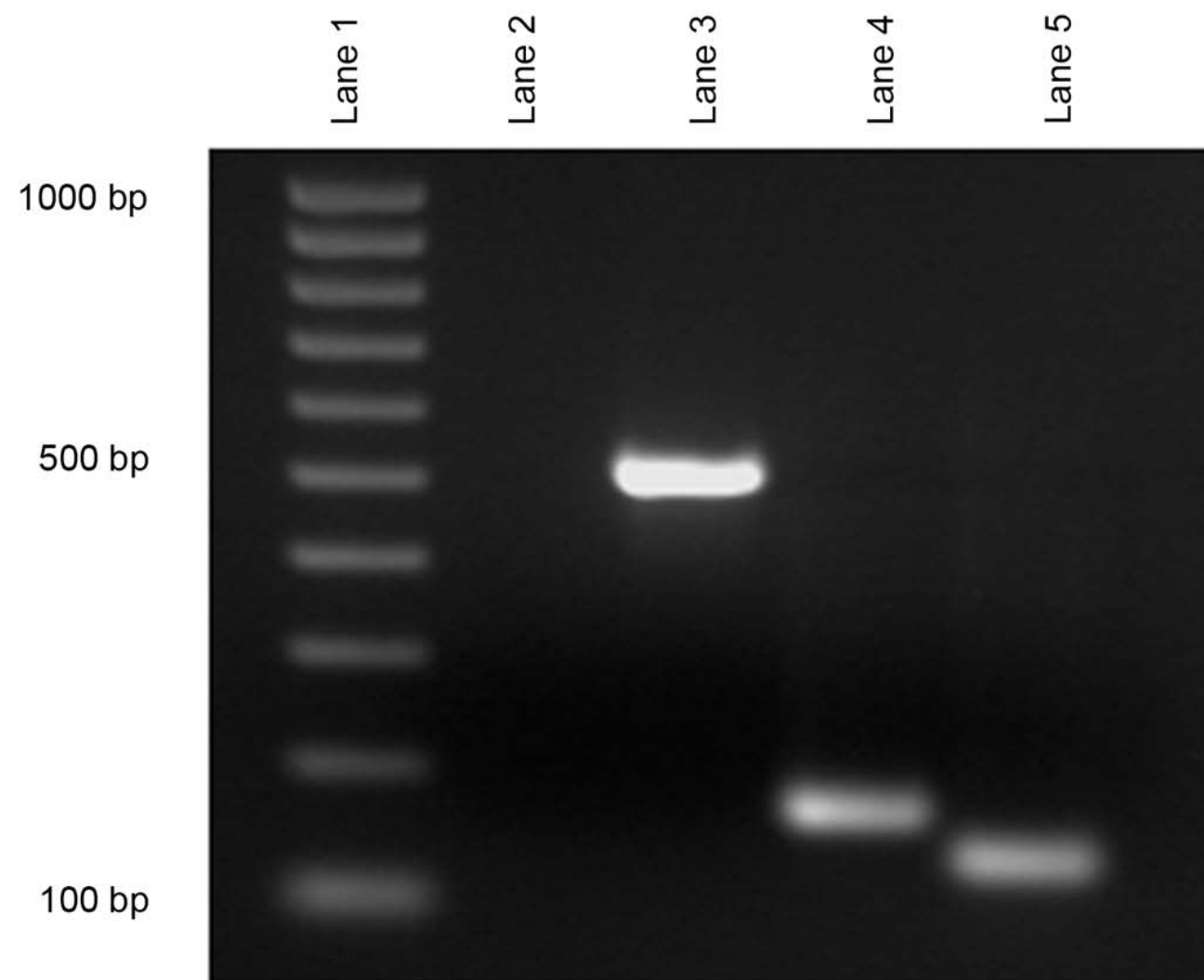

Supplement: Figure S3 — Experimental PCR Validation of mRNA transcripts associated with novel genes. (A) mRNA transcripts detected by tiling microarrays associated with novel 2009 genes BPSL1301.1 and BPSL2337.1. Top Row: Locations of Bp genes on Chromosome 1 on positive (+) and negative (−) strands. Novel genes validated are shown in red. Bottom row: Transcript expression on both the positive and negative strands. (B) RT-PCR validation of novel gene transcripts. Lane 1: Blank/Negative control (water); Lane 2: positive control: 16S rRNA; Lane 3: Novel gene BPSL1301.1; Lane 4: Novel gene BPSL2337.1. The 100 bp molecular weight ladder is shown on the left. (0.15 MB PDF) [file ppat.1000845.s003.pdf]

## A) Gene alignment

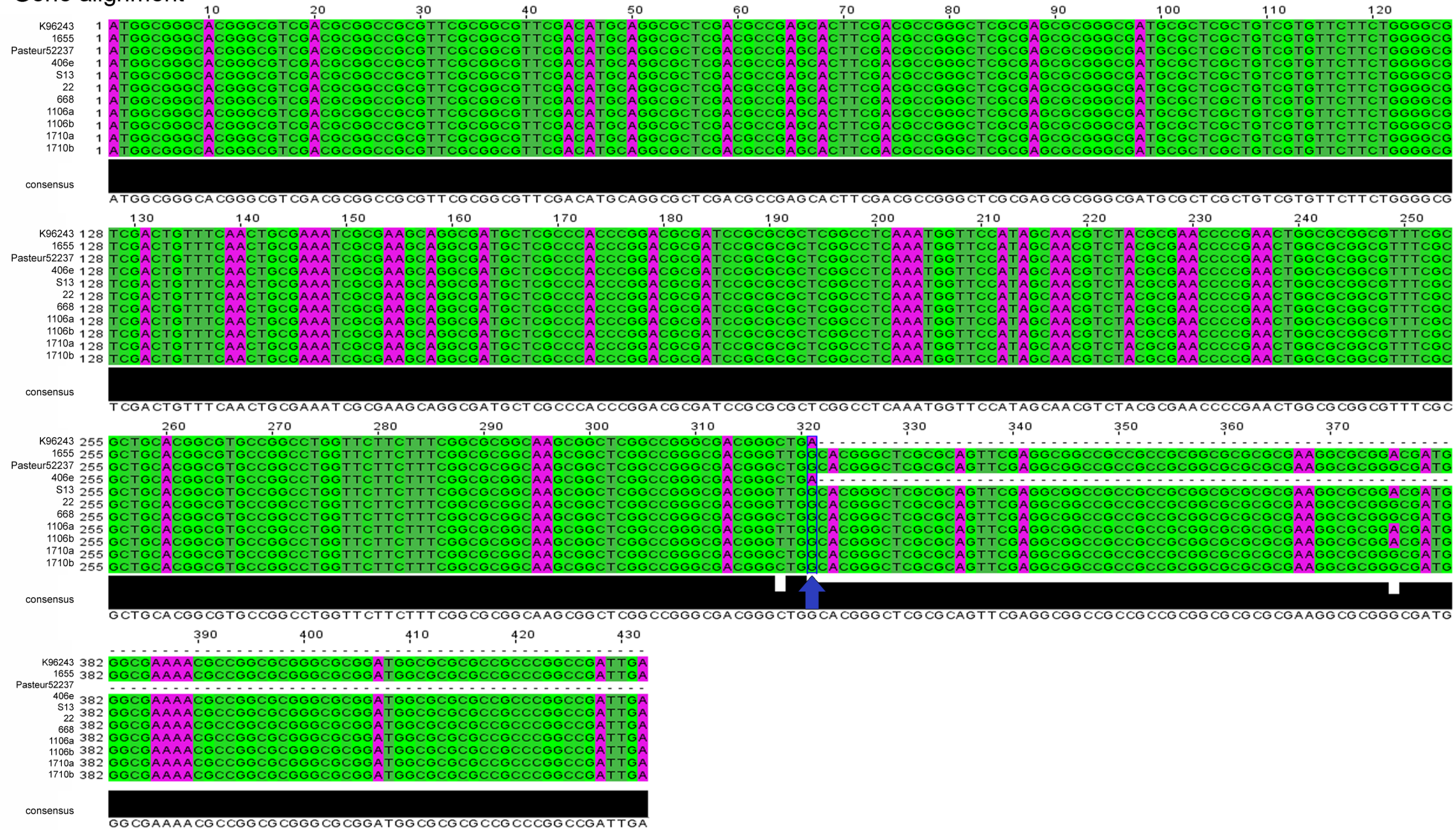

## B) Protein alignment

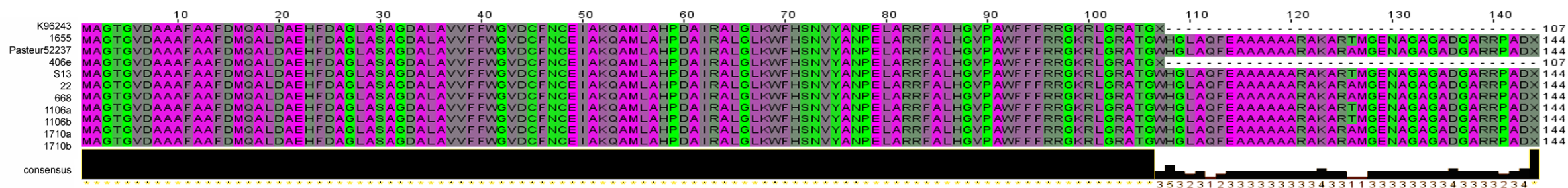

Supplement: Figure S4 — Example of a differential pseudogene. Multiple sequence alignment of BPSL2828 identified as a pseudogene in BpK96243, against its homologs from other sequenced Bp genomes [a) gene sequence b) protein sequence]. Alignments were performed using ClustalW [6]–[7]. The Bp strain names are indicated at the beginning of the alignment. The black bar at the bottom of the alignment indicates the consensus. The mutation is encircled by a blue box. (3.81 MB PDF) [file ppat.1000845.s004.pdf]

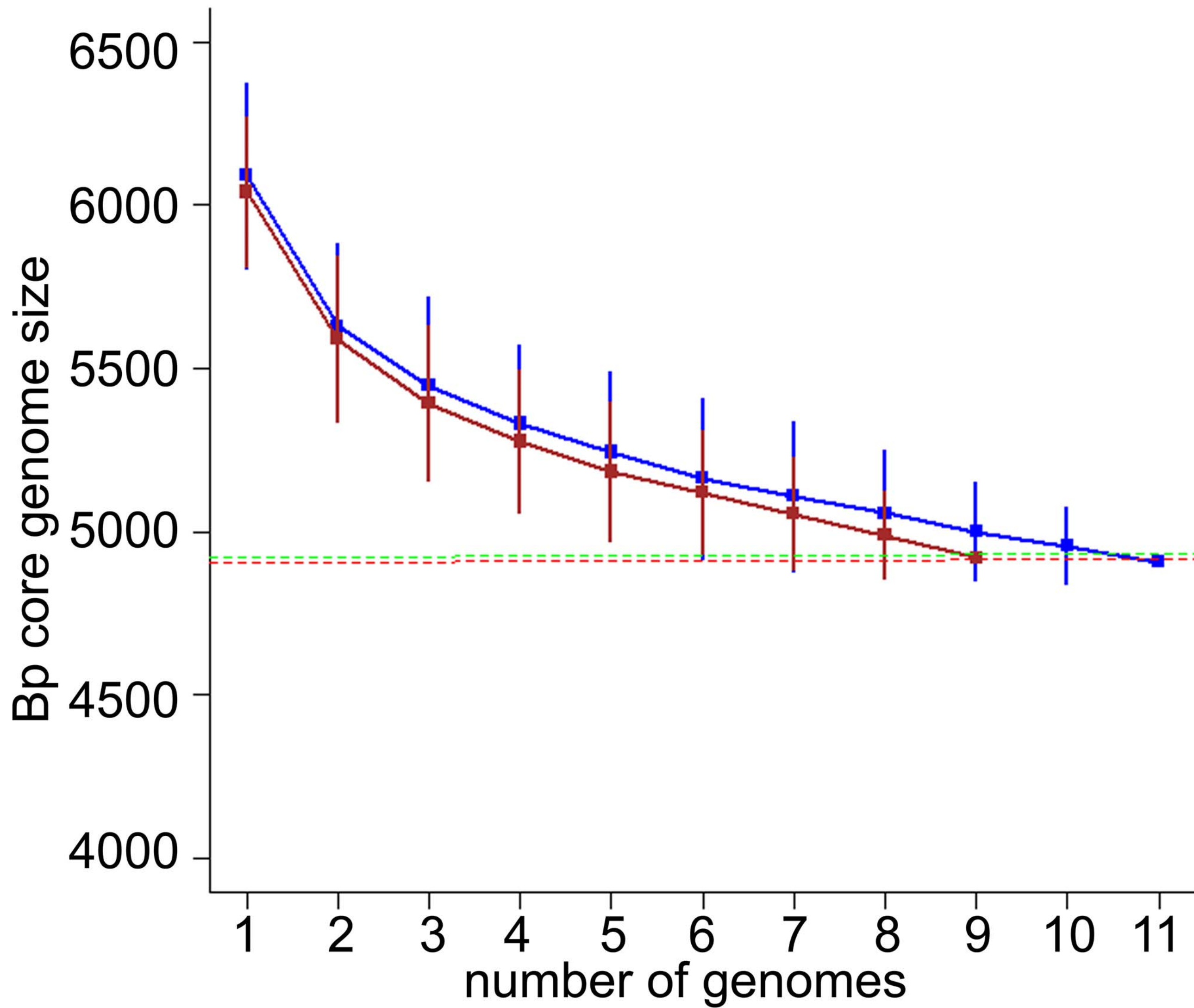

Supplement: Figure S5 — Bp Core genome estimates from nine isolates. Depletion curves for the Bp core genome (blue: 11 Bp genomes; brown: nine genomes representing independently-derived strains). Vertical bars represent standard deviation values based upon one hundred randomized input orders of the Bp genomes [8]. The analysis revealed a highly similar BpCG gene set based on 9 isolates, comprising 4920 ORFs (compared to the 4908 ORFs based on the 11-isolate analysis). (0.15 MB PDF) [file ppat.1000845.s005.pdf]

A)

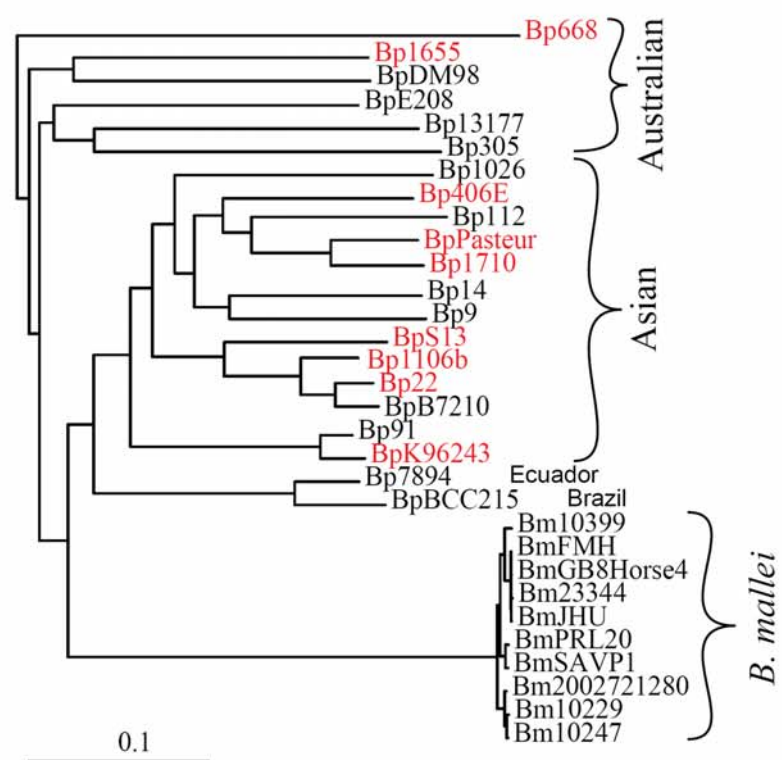

B)

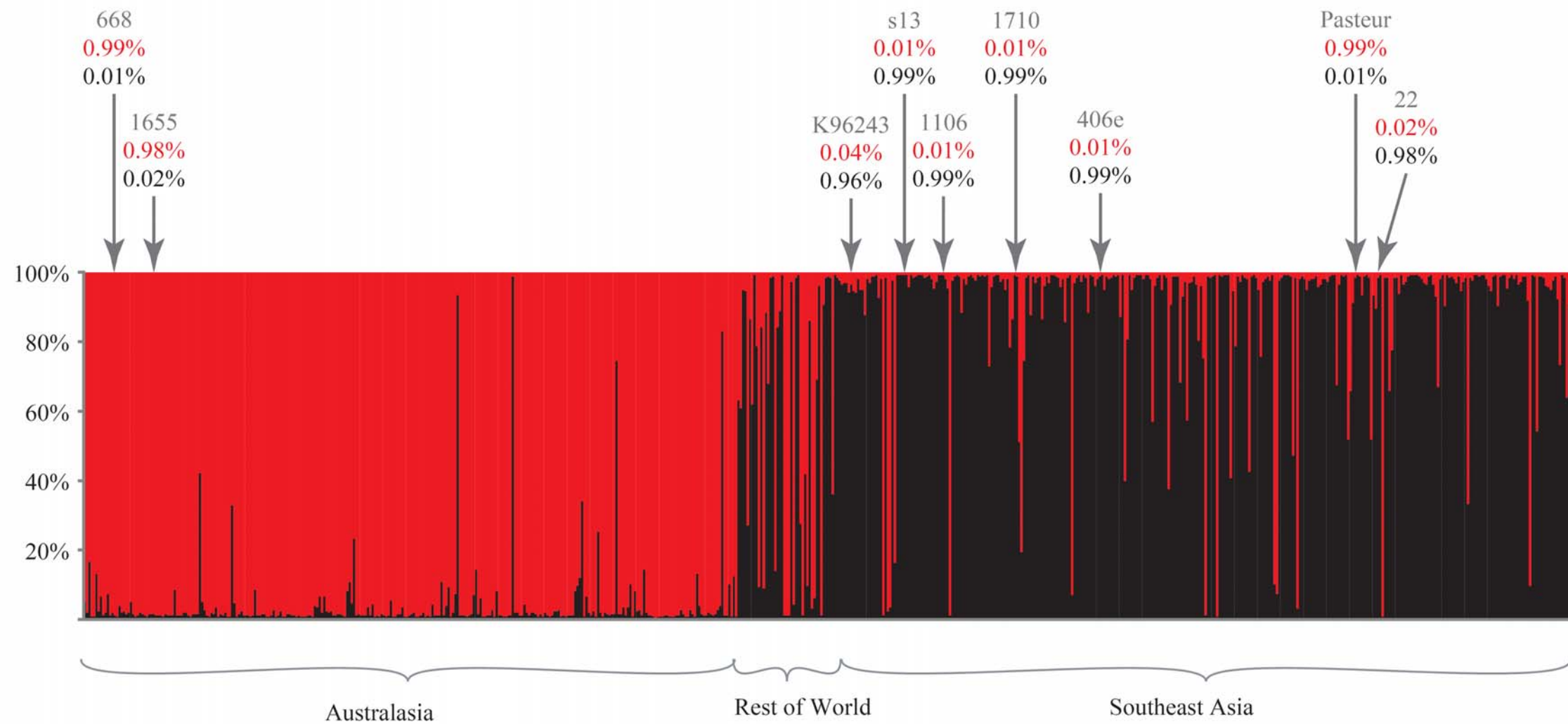

Supplement: Figure S6 — Phylogenetic and MLST Analysis of Sequenced Bp Strains. To infer phylogenetic relationships between the sequenced Bp strains, we generated phylogenetic trees based on whole-genome shotgun sequencing data of 33 Burkholderia strains, including 23 Bp strains and 10 B. mallei strains as an outlier group. Consistent with Figure 4A in the Main text, the two Australian strains (668 and 1655) segregated in phylogenetic subbranches distinct from the South-East Asian strains (Figure S6A). This phylogenetic separation was further supported by a larger MLST-based population genetic analysis of 1827 isolates (647 sequence types), confirming the division of Bp into two major populations (Figure S6B). These results suggest that there are two major populations of Bp, an Australian and a Southeast Asian population [5], and that the Australian population may be more ancient and more diverse than the Southeast Asian population. A) Phylogenetic relationships of Bp isolates used in this study compared to other Burkholderia isolates with whole genome sequences. This phylogeny contains 33 genomes of Bp and Bm and is based on 14,544 shared orthologous SNPs [5]. Genomes used in this study are shown in red. B) Estimated population structure of Bp and B. mallei using allele frequencies of MLST data. Each thin vertical line represents a sequence type that is divided into two portions that resemble the proportion of 5,000 iterations where that sequence type was assigned to each of two populations. The red population is dominated by sequence types from Australia, while the black population is dominated by sequence types of Southeast Asian origin. Geographic affiliations of sequence types are labeled below the figure. Isolates whose genomes were used in this study are indicated along with the percentage of iterations that assigned them to each population. Data used was downloaded from http://bpseudomallei.mlst.net/ on November 23rd, 2009. Isolates with no information on the country of origin were [file ppat.1000845.s006.pdf]

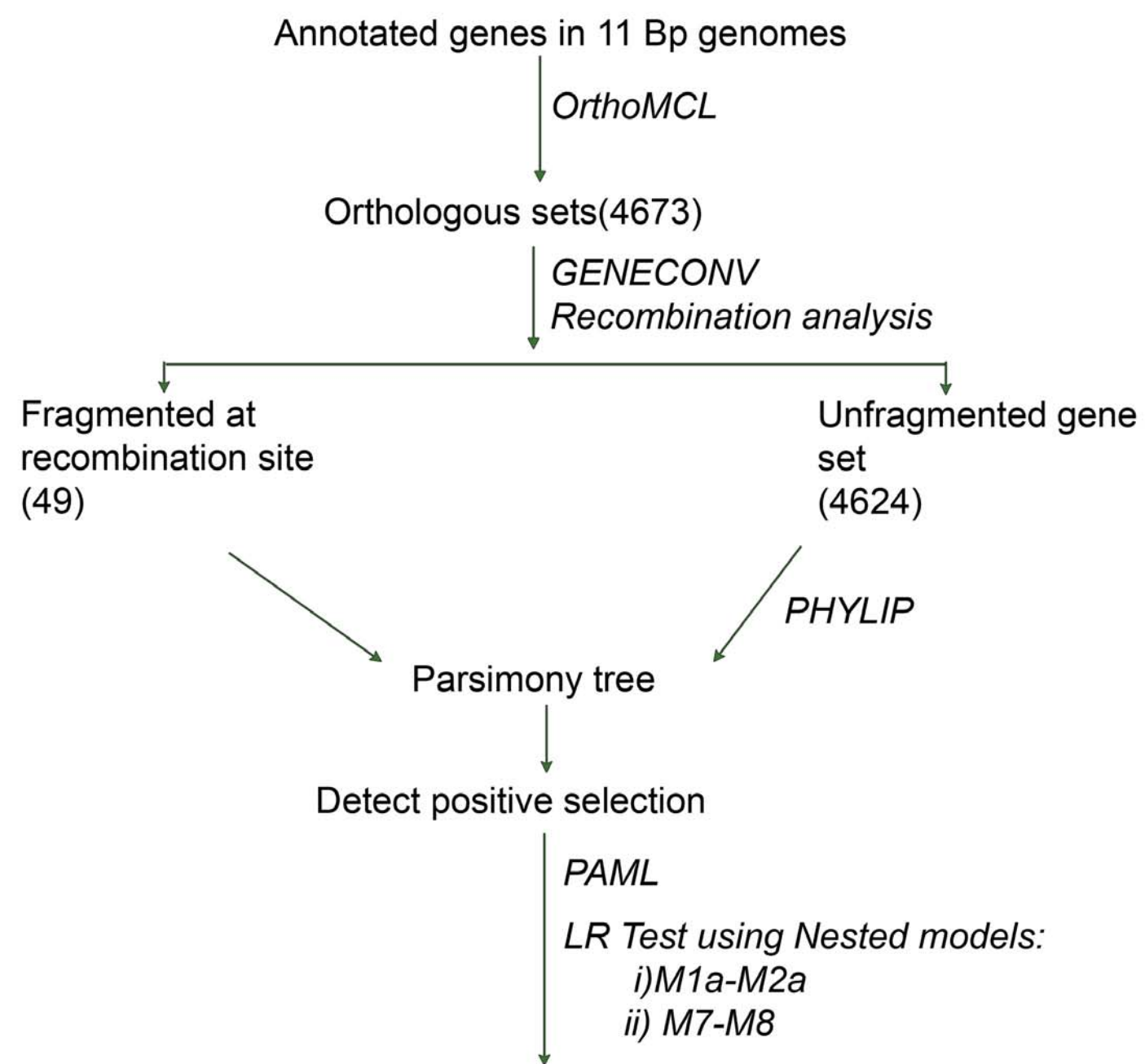

M1a-M2a

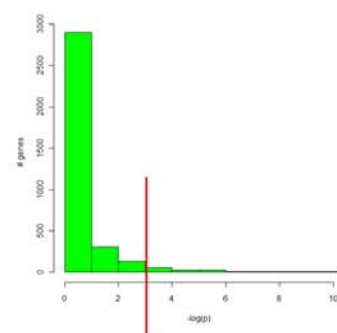

M7-M8

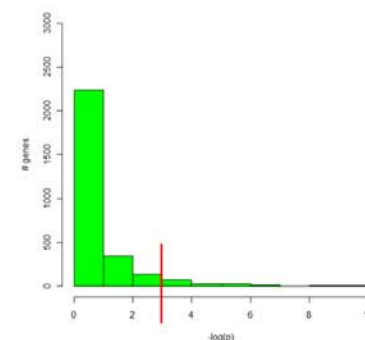

P value = 0.001

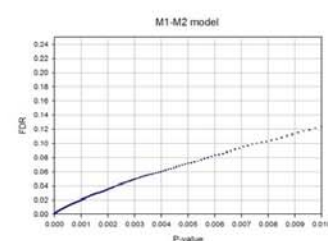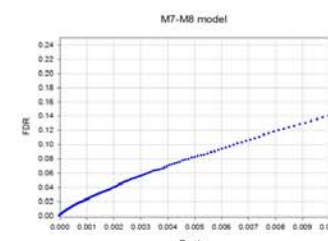

FDR ~2%

211 genes with signature  
of positive selection

Supplement: Figure S7 — Schematic of Positive Selection (PS) Analysis Workflow. Overview of the positive selection analysis scheme. Size of each dataset is indicated in parentheses. Programs used are indicated next to the arrows. (0.12 MB PDF) [file ppat.1000845.s007.pdf]

A)

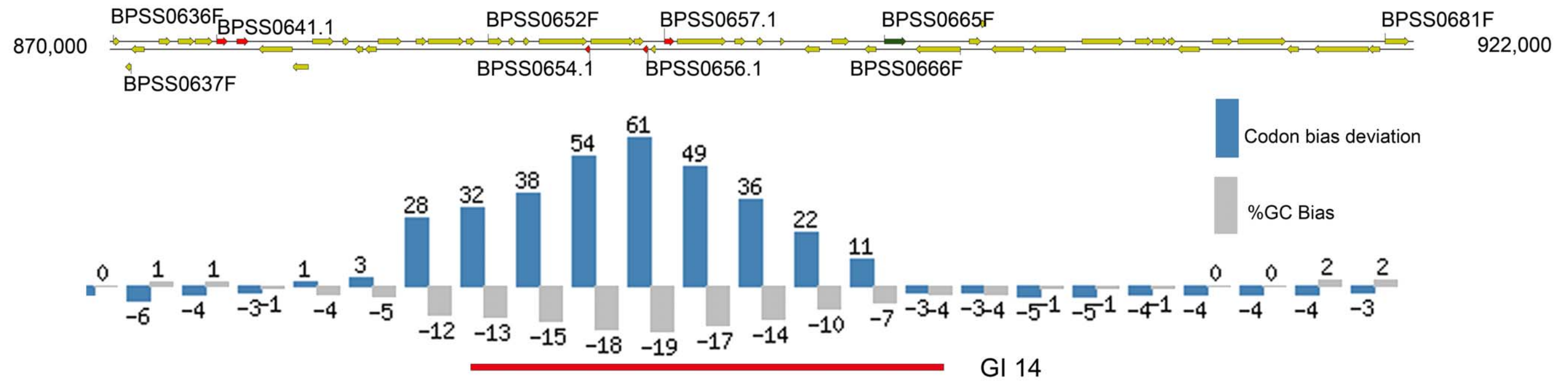

B)

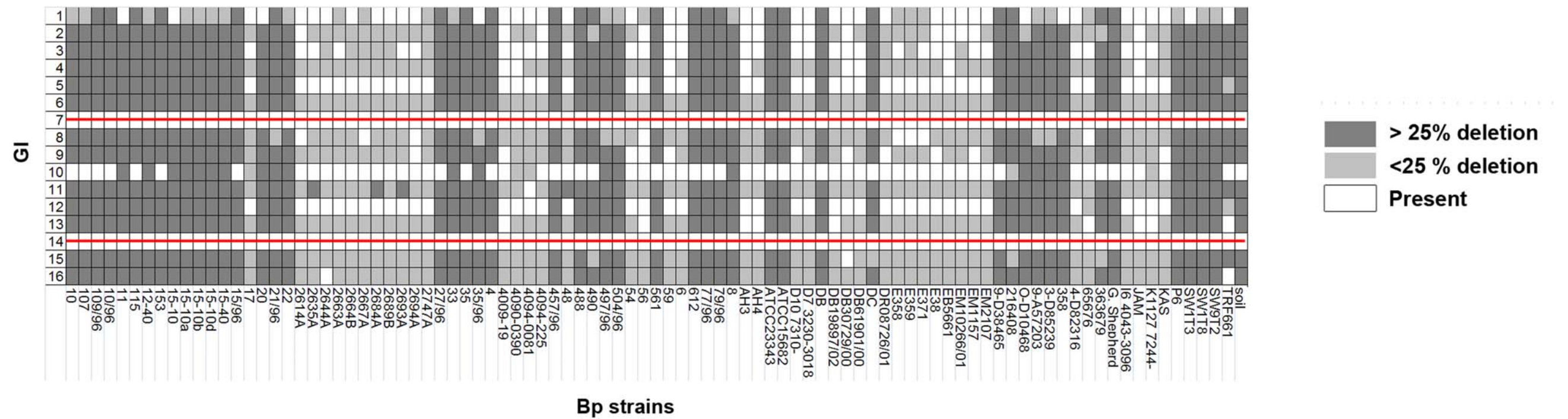

C)

i) Carbon Source

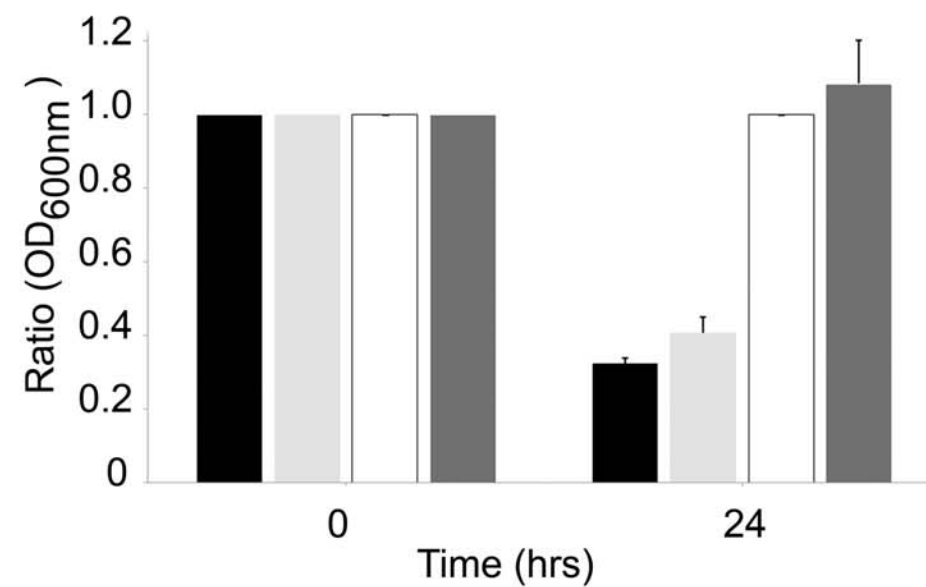

ii) Nitrogen Source

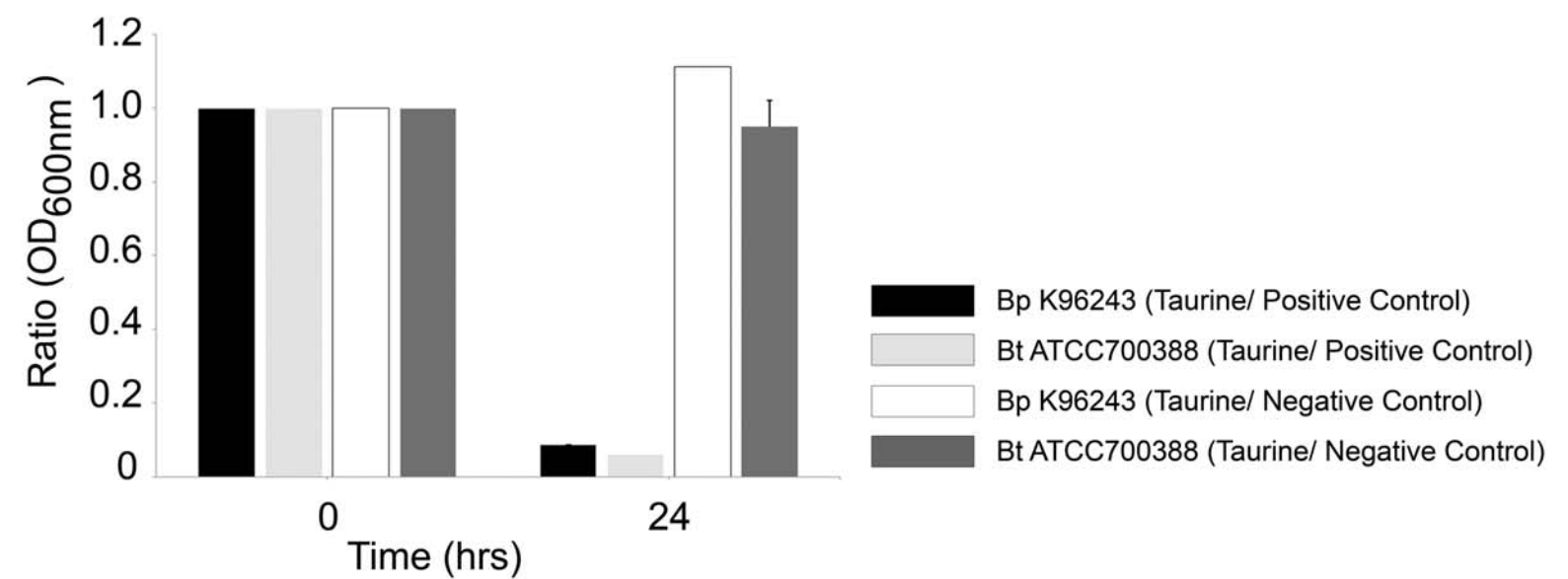

Supplement: Figure S8 — Expansion of tauD Taurine Dioxygenase Genes in Bp. A) Row 1: Genome organization of GI14 (BPSS0652-BPSS0666) and surrounding regions on Burkholderia pseudomallei K96243 chromosome 2. Row 2: Columns represent codon bias deviation (dark blue) and %GC bias (grey) respectively, using a six-gene sliding window. Values were obtained using PredictBias Server [10]. The location of GI14 is shown in red at the bottom, corresponding to a region of codon bias and atypical GC content. The tauD gene BPSS0665F is highlighted in red. B) Hardwiring of GI14 in the Bp genome. Presence and absence of all 16 GIs were assessed in a panel of 98 Bp isolates by aCGH [11]. Both GI7 and GI14 (marked in red) are present in all Bp strains. C) Utilization of taurine as the sole i) carbon source or ii) nitrogen source by Bp K96243 and Bt ATCC700388. Cultures with taurine as the sole carbon and nitrogen source showed comparable growth with the respective negative controls, which is significantly less than the respective positive controls. Error bars represent the standard deviations between replicate cultures. (0.30 MB PDF) [file ppat.1000845.s008.pdf]
