# Supplementary material for: A Genomic Survey of Positive Selection in Burkholderia pseudomallei Provides Insights into the Evolution of Accidental Virulence
Source: PLoS Pathog. 2010 Apr 1;6(4):e1000845. doi: 10.1371/journal.ppat.1000845 (PMC2848565; doi:10.1371/journal.ppat.1000845)
Supplement: Figure S2 — Bp transcript expression is associated with previously-identified genes. Top Row: Locations of 5 Bp genes on Chr 1 (green bars) and 6 Bp genes on Chr2 (green bars) on the positive (+) and negative (−) strands. All 10 genes are commonly found in both the 2004 and 2009 annotations. Bottom row: Transcript expression on both positive and negative strands as measured using tiling microarrays. Notice that the transcripts are tightly associated with the previously-identified genes. Red regions likely correspond to either 5′ or 3′ untranslated UTR regions. (0.13 MB PDF) [file ppat.1000845.s002.pdf]

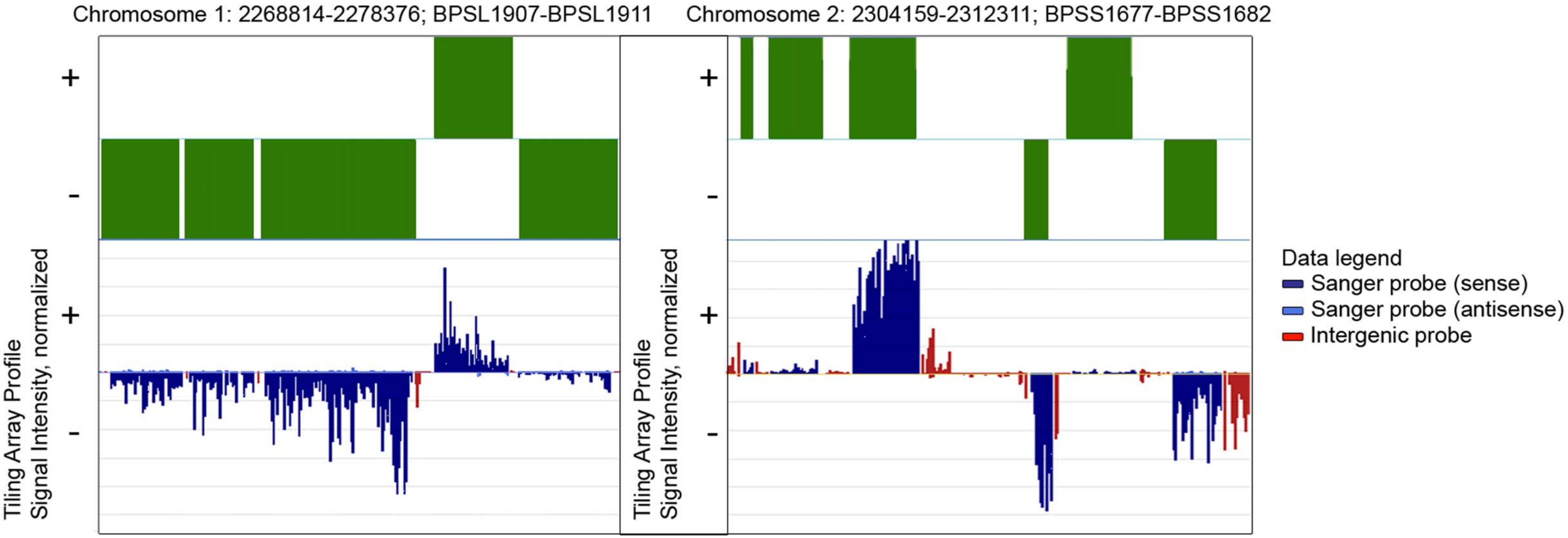

Chromosome 1: 2268814-2278376; BPSL1907-BPSL1911
Chromosome 2: 2304159-2312311; BPSS1677-BPSS1682
+
-
+
-
Tiling Array Profile
Signal Intensity, normalized
Data legend
Sanger probe (sense)
Sanger probe (antisense)
Intergenic probe
