# Supplementary material for: A Genomic Survey of Positive Selection in Burkholderia pseudomallei Provides Insights into the Evolution of Accidental Virulence
Source: PLoS Pathog. 2010 Apr 1;6(4):e1000845. doi: 10.1371/journal.ppat.1000845 (PMC2848565; doi:10.1371/journal.ppat.1000845)
Supplement: Table S1 — List of B. pseudomallei Strains (0.08 MB PDF) [file ppat.1000845.s009.pdf]

Table S1: List of *B. pseudomallei* Strains

S.No	Strain	Sequenced by	Original Source	Clinical Data	MLST
1	K96243	Sanger	Thailand	Isolated in 1996 from a 34 year old female diabetic patient at Khon Kaen hospital, northeast with a clinical history of short incubation, septicemic infection, and rapid progression to death.	ST 10
2	1655	JCVI	Australia	From Menzies School of Health Research, Australia. 2003 sputum isolate from a 64 year old female patient from Darwin treated at Royal Darwin Hospital for chronic pulmonary melioidosis on a background of severe bronchiectasis	ST 131
3	Pasteur 52237	JCVI	Vietnam	Obtained from Finkelstein collection. Am. J. Trop. Med. Hyg. 62:232-239, 2000.	ST 411
4	406e	JCVI	Bangkok	Isolated from toe swab in 1988 from a 21-year old male labourer presenting to Sappasithiprasong hospital. Disseminated disease (bacteremia, lung, skin, renal tract involvement). Died on second day of admission.	ST 211
5	S13	JCVI	Singapore	Mucoid environmental strain.	ST 51
6	22	GIS	Singapore	Isolated from National Service man in 1989.	ST423
7	668	JCVI	Australia	1995 blood culture isolate from a 53 year old male patient with severe melioidosis encephalomyelitis. Required prolonged ventilation at Royal Darwin Hospital ICU but survived with good cognitive function but residual hemiparesis.	ST 129
8	1106a	JCVI	Bangkok	Isolated from pus aspirated from liver abscess in 1993 from a 23-year old female rice farmer presenting to Sappasithiprasong hospital. Risk factors: thalassaemia and splenectomy. Multiple hepatic abscesses. Survived to discharge.	ST 70
9	1106b	JCVI	Bangkok	Isolated from pus aspirated from liver abscess in 1996 from patient described above. Survived to discharge.	ST 70
10	1710a	JCVI	Bangkok	Isolated from blood culture in 1996 from a 52-year old male rice farmer with a new diagnosis of diabetes mellitus presenting to Sappasithiprasong hospital. Disseminated disease (bacteremia plus lung and soft tissue involvement). Survived to discharge.	ST 177
11	1710b	JCVI	Bangkok	Isolated from blood culture in March 1999 from patient described above. Died on day of admission.	ST 177
