# Supplementary material for: A Genomic Survey of Positive Selection in Burkholderia pseudomallei Provides Insights into the Evolution of Accidental Virulence
Source: PLoS Pathog. 2010 Apr 1;6(4):e1000845. doi: 10.1371/journal.ppat.1000845 (PMC2848565; doi:10.1371/journal.ppat.1000845)
Supplement: Table S2 — Novel genes supported by two or three lines of evidence (0.06 MB PDF) [file ppat.1000845.s010.pdf]

Table S2: Novel genes supported by two or three lines of evidence

A. Novel genes supported by two lines of evidence

BPSL0021.1, BPSL0025.1, BPSL0068.1, BPSL0080.1, BPSL0085.1, BPSL0113.1, BPSL0113A.1, BPSL0158.1, BPSL0175.1, BPSL0176.2, BPSL0186.1, BPSL0200.1, BPSL0240.1, BPSL0294.2, BPSL0294.3, BPSL0326.1, BPSL0466.1, BPSL0494.1, BPSL0503.1, BPSL0504.1, BPSL0551.3, BPSL0568.1, BPSL0577.2, BPSL0616.3, BPSL0735.4, BPSL0735.6, BPSL0738.1, BPSL0753.1, BPSL0762.1, BPSL1057F1, BPSL1057.1, BPSL1057.2, BPSL1057.3, BPSL1108.1, BPSL1133.1, BPSL1246.1, BPSL1252.1, BPSL1269.1, BPSL1344.1, BPSL1393.1, BPSL1409.1, BPSL1544.1, BPSL1550.1, BPSL1637.1, BPSL1658.1, BPSL1660.1, BPSL1705.1, BPSL1758.1, BPSL1794.1, BPSL1851.1, BPSL1884.1, BPSL1935.1, BPSL1945.1, BPSL1948.1, BPSL2036.1, BPSL2071.1, BPSL2100.1, BPSL2126.2, BPSL2140.1, BPSL2192.1, BPSL2196.2, BPSL2199.1, BPSL2297.1, BPSL2346.1, BPSL2377.1, BPSL2439.1, BPSL2451.1, BPSL2451.2, BPSL2470A.1, BPSL2524.1, BPSL2569.1, BPSL2586.1, BPSL2614.1, BPSL2699.1, BPSL2729.1, BPSL2758.1, BPSL2911.1, BPSL2912.1, BPSL3046.1, BPSL3061.1, BPSL3065.1, BPSL3065.2, BPSL3066.1, BPSL3262.1, BPSL3280.1, BPSL3336.1, BPSL3338.1, BPSS0012.1, BPSS0033.1, BPSS0054.1, BPSS0057.1, BPSS0065.1, BPSS0143.1, BPSS0143.2, BPSS0199.1, BPSS0225.1, BPSS0287.1, BPSS0351.2, BPSS0384.1, BPSS0391A.1, BPSS0563.1, BPSS0641.1, BPSS0641.2, BPSS0706.1, BPSS0709.1, BPSS0717.1, BPSS0740.1, BPSS0826.1, BPSS0880.1, BPSS0885.1, BPSS0944.1, BPSS0956.1, BPSS0973.1, BPSS1002.1, BPSS1002.2, BPSS1108.1, BPSS1151.1, BPSS1213.1, BPSS1213.2, BPSS1296.1, BPSS1355.1, BPSS1362.1, BPSS1363.1, BPSS1384.1, BPSS1421.1, BPSS1471.1, BPSS1571.1, BPSS1575.1, BPSS1588.1, BPSS1588.2, BPSS1635.3, BPSS1720.1, BPSS1821.2, BPSS1849.1, BPSS1879.1, BPSS1897.1, BPSS1907.1, BPSS2000.1, BPSS2003.1, BPSS2052.1, BPSS2056.1, BPSS2056.2, BPSS2057.1, BPSS2225.1, BPSS2268.1, BPSS2344.1, BPSS2348.1, BPSL1153.1, BPSL3228.1, BPSL1246.4, BPSL3228.2, BPSL3348.1, BPSL2974.1, BPSL1631.1, BPSL0735.2, BPSL1850.1, BPSL3254A.1, BPSL2036.2, BPSL0735.3, BPSL3281.1, BPSL2743.1, BPSL2598.1, BPSL1128.1, BPSL3071.1, BPSL0256.1, BPSL2337.2, BPSL0055.1, BPSL1823.1, BPSL1787.1, BPSS2351.2, BPSS0707.1, BPSS1089.1, BPSS1213.3, BPSS0350.3, BPSS0632.1, BPSS2036.1, BPSS1993.2, BPSS1815.1, BPSS0989.1, BPSS0957.1, BPSS0579.1, BPSS1492.1, BPSS1159.1, BPSS1998.1, BPSS1845.1, BPSS1728A.1, BPSS1098.1, BPSS0878.1, BPSS0795.3, BPSS0795.1, BPSS0746.2, BPSS2269.1, BPSS1709.1, BPSS1648.1, BPSS1568.1, BPSS1489.1, BPSS1860.1, BPSS0746.1, BPSS2316.1, BPSS1002.3, BPSS0946.1, BPSS1704.1

B. Novel genes supported by three lines of evidence

BPSL0079.1, BPSL0294.1, BPSL0294.4, BPSL0393.1, BPSL0407.1, BPSL0429.1, BPSL0499.1, BPSL0706.1, BPSL0762.2, BPSL0887.1, BPSL0938A.2, BPSL0991.1, BPSL1043.1, BPSL1246.2, BPSL1301.1, BPSL1304B.1, BPSL1311.1, BPSL1323.1, BPSL1449.1, BPSL1618.1, BPSL1658.2, BPSL1672.1, BPSL1794.2, BPSL1970.1, BPSL2036.3, BPSL2112.1, BPSL2126.1, BPSL2279.1, BPSL2304.1, BPSL2355.1, BPSL2367.1, BPSL2422.1, BPSL2554.1, BPSL2880.1, BPSL2882.1, BPSL3038.2, BPSL3062.1, BPSL3228.4, BPSL3376.1, BPSL3403.1, BPSS0035.1, BPSS0058.1, BPSS0063.1, BPSS0063.2, BPSS0138.1, BPSS0192.1, BPSS0229.1, BPSS0233a.1, BPSS0279.1, BPSS0350.1, BPSS0351.1, BPSS0603.1, BPSS0709.2, BPSS0809.1, BPSS0812A.1, BPSS0818.1, BPSS1043.1, BPSS1090.1, BPSS1111.1, BPSS1115.1, BPSS1202.1, BPSS1216.1, BPSS1217.1, BPSS1240.1, BPSS1259.1, BPSS1552.1, BPSS1555.1, BPSS1561.1, BPSS1635.1, BPSS1656.1, BPSS1682.1, BPSS1754.1, BPSS1877.1, BPSS1927.1, BPSS1960.1, BPSS2052.2, BPSS2053.1, BPSS2130.2, BPSS2171.1, BPSS2208.1, BPSS2329.1
