# Supplementary material for: A Genomic Survey of Positive Selection in Burkholderia pseudomallei Provides Insights into the Evolution of Accidental Virulence
Source: PLoS Pathog. 2010 Apr 1;6(4):e1000845. doi: 10.1371/journal.ppat.1000845 (PMC2848565; doi:10.1371/journal.ppat.1000845)
Supplement: Table S3 — Compositional Features of Novel Predicted Genes and Short-Length Sanger Genes Compared to All Sanger Genes. *Sanger genes less than 200 aa were defined as “short length”. All p-values were determined using an unpaired two tailed t-test (unequal variance). (0.06 MB PDF) [file ppat.1000845.s011.pdf]

Table S3: Compositional Features of Novel Predicted Genes and Short-Length Sanger Genes Compared to All Sanger Genes

A) Comparison between Sanger Genes (5728) vs Novel Genes (282)

Sanger genes : 5728 # Novel genes : 282

	Length (aa)	CAI	GC	Hydrophobicity
Sanger genes (5728)	348 ± 307	0.29 ± 0.04	0.68 ± 0.05	-0.03 ± 0.38
Novel genes (280)	98 ± 56	0.22 ± 0.07	0.63 ± 0.10	-0.45 ± 0.50
P-value	*1.2347E-304*	*2.16158E-54*	*9.688E-17*	*3.19675E-34*

B) Comparison between Sanger Genes (5728) vs Short-length Sanger Genes (1587)

Total Sanger genes : 5728 # Sanger genes (≤200aa*) : 1587

	CAI	GC	Hydrophobicity
All Sanger genes (5728)	0.29 ± 0.04	0.68 ± 0.05	-0.03 ± 0.38
Short Sanger genes (1587)	0.29 ± 0.06	0.66 ± 0.05	-0.12 ± 0.48
P-value	*0.000449302*	*1.83603E-34*	*1.55853E-13*
