# Supplementary material for: A Genomic Survey of Positive Selection in Burkholderia pseudomallei Provides Insights into the Evolution of Accidental Virulence
Source: PLoS Pathog. 2010 Apr 1;6(4):e1000845. doi: 10.1371/journal.ppat.1000845 (PMC2848565; doi:10.1371/journal.ppat.1000845)
Supplement: Table S4 — Previously-predicted BpK96243 genes missed by the FGENESB pipeline (0.11 MB PDF) [file ppat.1000845.s012.pdf]

Table S4: Previously-predicted BpK96243 genes missed by the FGENESB pipeline

Sanger genes missed by FGENESB	Reported Features (2004 annotation)	Functional annotation
BPSL0557	No significant database matches. Doubtful CDS	hypothetical protein
BPSL0574A	No significant database matches	hypothetical protein
BPSL0574B	No significant database matches	hypothetical protein
BPSL0739	Doubtful CDS. No significant database matches	hypothetical protein
BPSL0740	Doubtful CDS. No significant database matches	hypothetical protein
BPSL0747a	Doubtful CDS. No significant database matches	hypothetical protein
BPSL0760	No significant database matches	hypothetical protein
BPSL0771	No significant hits in the databases. Possible alternative start at codon 10	hypothetical protein
BPSL0844a	Doubtful CDS. No significant database matches	hypothetical protein
BPSL0954	Doubtful CDS. No significant database matches	hypothetical protein
BPSL1014	Doubtful CDS. No significant database matches	hypothetical protein
BPSL1038a	Doubtful CDS. No significant database matches	hypothetical protein
BPSL1041	No significant database matches	hypothetical protein
BPSL1047	No significant database matches	hypothetical protein
BPSL1060	No significant database matches	hypothetical protein
BPSL1063	No significant database matches	hypothetical protein
BPSL1123b	Doubtful CDS. No significant database matches	hypothetical protein
BPSL1147	No significant database matches	hypothetical protein
BPSL1152	No significant database matches	hypothetical protein
BPSL1255	No significant database matches	hypothetical protein
BPSL1290a	Doubtful CDS. No significant database matches	hypothetical protein
BPSL1406A	No significant database matches	hypothetical protein
BPSL1621	No significant database matches	hypothetical protein
BPSL1624	No significant database matches	hypothetical protein
BPSL2037	No significant database matches	hypothetical protein
BPSL2044	No significant database matches	hypothetical protein
BPSL2072	No significant database matches	hypothetical protein
BPSL2173	Doubtful CDS. No significant database matches. Contains a repeat at the N-terminal region	hypothetical protein
BPSL2206	No significant database matches	hypothetical protein
BPSL2507A	Doubtful CDS. Poor database matches. Similar to Aeropyrum pernix hypothetical protein ape0269 SWALL:Q9YFH7 (EMBL:AP000058) (125 aa) fasta scores: E(): 1, 31.42% id in 105 aa	hypothetical protein
BPSL2549	No significant database matches	hypothetical protein
BPSL2571	Doubtful CDS. No significant database matches	hypothetical protein
BPSL2573	No significant database matches	hypothetical protein
BPSL2580	Doubtful CDS. No significant database matches	hypothetical protein
BPSL2584	No significant database matches	hypothetical protein

BPSL2768	No significant database matches	hypothetical protein
BPSL3266	Doubtful CDS. No significant database matches	hypothetical protein
BPSL3268	No significant database matches	putative membrane protein
BPSS0380A	Doubtful CDS. No significant database matches	hypothetical protein
BPSS0380B	Doubtful CDS. No significant database matches	hypothetical protein
BPSS0406	No significant database matches. Doubtful CDS	hypothetical protein
BPSS0769A	Doubtful CDS. Similar to the C-terminal region of Azoarcus evansii PaaB SWALL:Q9F9U8 (EMBL:AF176259) (103 aa) fasta scores: E(): 1.4, 50% id in 52 aa	hypothetical protein
BPSS0874	No significant database matches. Doubtful CDS	hypothetical protein
BPSS1513	No significant database matches. Doubtful CDS.	hypothetical protein
BPSL0094	Poor database matches. Similar to Ralstonia solanacearum probable lipoprotein rsc2264 or rs01295 SWALL:Q8XX52 (EMBL:AL646069) (50 aa) fasta scores: ():1.6e-12, 74.51% id in 51 aa, and to the N-terminal region of Deinococcus radiodurans hypothetical protein DR1795 SWALL:Q9RTG9 (EMBL:AE002020) (264 aa) fasta scores: E(): 6.5, 40.38% id in 52 aa	putative lipoprotein
BPSL0139	Poor database matches. Similar to an internal region of Salmonella typhimurium and Salmonella typhi hypothetical protein STM2627 or STM1013 or STY1021 SWALL:O84891 (EMBL:AF001386) (124 aa) fasta scores: E(): 5.3, 31.76% id in 85 aa	putative phage DNA-binding protein
BPSL0167	No significant database matches. Similar to BPSS1068, 97.590% identity (97.590% ungapped) in 83 aa overlap	hypothetical protein
BPSL0552	No significant database matches	hypothetical protein
BPSL0553	Poor database matches. N-terminus is weakly similar to the N-terminal region of Rhizobium etli hypothetical protein YH035 SWALL:AAM54968 (EMBL:U80928) (101 aa) fasta scores: E(): 0.0068, 40.84% id in 71 aa	putative DNA-binding protein
BPSL0110A	Weakly similar to the N-terminal regions of Streptomyces avermitilis hypothetical protein SWALL:Q82F88 (EMBL:AP005038) (164 aa) fasta scores: E(): 2.1, 33.33% id in 96 aa, and Streptomyces coelicolor putative transcriptional regulator SC4C6.04 SWALL:Q9XAN6 (EMBL:AL939128) (204 aa) fasta scores: E(): 5.2, 35% id in 80 aa. CDS contains a frameshift after codon 77	conserved hypothetical protein (fragment)
BPSL0708	Probable gene remnant. Similar to internal regions of Escherichia coli aerobic C4-dicarboxylate transport protein DctA SWALL:DCTA_ECOLI (SWALL:P37312) (428 aa) fasta scores: E(): 5.5e-14, 38.02% id in 142 aa, and Ralstonia solanacearum C4-dicarboxylate transport protein 3 rsp0995 or rs02327 SWALL:DTA3_RALSO (SWALL:Q8XR66) (447 aa) fasta scores: E(): 3.7e-14, 38.46% id in 143 aa	putative transport protein (fragment)
BPSL0951	Similar to the N-terminal regions of Ralstonia solanacearum ISRso14-transposase orfB protein SWALL:Q8XF72 (EMBL:AL646083) (275 aa) fasta scores: E(): 4.4e-14, 75.47% id in 53 aa, and Pseudomonas aeruginosa hypothetical protein PA0987 SWALL:Q9I4Y2 (EMBL:AE004531) (280 aa) fasta scores: E(): 6e-08, 54.9% id in 51 aa. Probable gene remnant	insertion element hypothetical protein(fragment)
BPSL1058	Gene remnant. Similar to the C-terminal regions of Bradyrhizobium japonicum ID49 rsbeta5 SWALL:Q9ANP4 (EMBL:AF322012) (275 aa) fasta scores: E(): 3.4e-22, 58% id in 100 aa, and to Rhizobium meliloti putative transposase protein r03101 or smc03248 SWALL:Q92LG0 (EMBL:AL591792) (276 aa) fasta scores: E(): 2.4e-20, 55.44% id in 101 aa	putative transposase (fragment)

BPSL1371B	Probable gene remnant. Similar to the N-terminal region of Bordetella bronchiseptica phage-related integrase SWALL:Q7WFY2 (EMBL:BX640449) (424 aa) fasta scores: E(): 0.68, 25.64% id in 78 aa, and Xanthomonas axonopodis pv. citri str. 306 phage-related integrase Int SWALL:Q8PKF1 (EMBL:AE011860) (399 aa) fasta scores: E(): 0.76, 29.11% id in 79 aa	putative phage-related integrase (fragment)
BPSL1371C	Probable gene remnant. Similar to the C-terminal regions of Burkholderia thailandensis GP33 SWALL:Q8VP93 (EMBL:AY063741) (223 aa) fasta scores: E(): 1.5e-22, 84.41% id in 77 aa, and Chromobacterium violaceum hypothetical protein SWALL:Q7NVB7 (EMBL:AE016918) (214 aa) fasta scores: E(): 4.6e-07, 42.66% id in 75 aa. CDS contains a frameshift after codon 32	conserved hypothetical protein (fragment)
BPSL1384a	Probable gene remnant. C-terminal region is similar to the C-terminus of Burkholderia thailandensis temperate bacteriophage protein Gp27 SWALL:Q8VP99 (EMBL:AY063741) (146 aa) fasta scores: E(): 6.9e-14, 86.66% id in 45 aa, and to bacteriophage phiE125 protein Gp27 SWALL:Q8W6S4 (EMBL:AF447491) (262 aa) fasta scores: E(): 1.1e-13, 86.66% id in 45 aa	putative phage-related protein (fragment)
BPSL1391	Partial CDS. Similar to the C-terminal region of Bacteriophage phiE125 gp27 SWALL:Q8W6S4 (EMBL:AF447491) (262 aa) fasta scores: E(): 2.3e-17, 75.75% id in 66 aa	putative phage related protein (fragment)
BPSL1584	Similar to internal regions of Streptomyces verticillus peptide synthetase NRPS12 SWALL:Q9FB39 (EMBL:AF210249) (578 aa) fasta scores: E(): 7.3e-19, 41.29% id in 201 aa, and Streptomyces lavendulae peptide synthetase SWALL:Q93N89 (EMBL:AF386507) (2116 aa) fasta scores: E(): 3.3e-18, 45.4% id in 174 aa	putative peptide synthetase (fragment)
BPSL1668A	Similar to internal regions of Ralstonia solanacearum ISRso5-transposase protein TISRso5 SWALL:Q8Y371 (EMBL:AL646057) (363 aa) fasta scores: E(): 4.4e-07, 79.41% id in 34 aa, and to Agrobacterium tumefaciens transposase Orf1 SWALL:Q44454 (EMBL:Z18270) (366 aa) fasta scores: E(): 0.18, 54.54% id in 33 aa	putative transposase protein (fragment)
BPSL1697	Similar to parts of Ralstonia solanacearum putative integrase/recombinase protein rsp0902 or rs01673 SWALL:Q8XRF2 (EMBL:AL646081) (566 aa) fasta scores: E(): 4.4e-54, 58.68% id in 259 aa. Note: Contains frameshifts at residues 46 and 116 and lack an appropriate start codon	putative recombinase (fragment)
BPSL1703	Gene remnant, similar to internal regions of Escherichia coli transposase InsD for insertion element IS2 SWALL:INSD_ECOLI (SWALL:P19777) (301 aa) fasta scores: E(): 2e-18, 51.93% id in 129 aa, and Ralstonia solanacearum ISRso10-transposase OrfB protein rsc1830 or rs04263 SWALL:Q8XYC8 (EMBL:AL646066) (282 aa) fasta scores: E(): 8e-32, 67.66% id in 133 aa	transposase (fragment)
BPSL1708A	Similar to parts of Caulobacter crescentus insertion sequence IS511 ORFA and ORFB genes, complete CDS cc2742 and cc0515 and cc2290 and cc2690 SWALL:Q45993 (EMBL:U39501) (308 aa) fasta scores: E(): 2.8e-10, 48.75% id in 80 aa, and of Rhizobium loti putative transposase for insertion sequence MSI240 SWALL:CAD31272 (EMBL:AL672114) (309 aa) fasta scores: E(): 1.8e-09, 46.25% id in 80 aa	putative insertion element (fragment)
BPSL1949	Partial CDS. Similar to the N-terminal region of Pseudomonas aeruginosa hypothetical protein pa2566 SWALL:Q9I0R9 (EMBL:AE004684) (395 aa) fasta scores: E(): 2.4e-60, 59.09% id in 264 aa, and of Pyrobaculum aerophilum flavoprotein reductase, conjectural pae2618 SWALL:Q8ZUT5 (EMBL:AE009886) (380 aa) fasta scores: E(): 6.5e-19, 30.11% id in 259 aa. Note: CDS lacks a stop codon	conserved hypothetical protein (fragment)

BPSL2023	Gene remnant. Similar to the C-terminal region of Escherichia coli trans-aconitate 2-methyltransferase Tam or b1519 SWALL:TAM_ECOLI (SWALL:P76145) (251 aa) fasta scores: E(): 0.014, 31.42% id in 70 aa, and of Pseudomonas aeruginosa trans-aconitate 2-methyltransferase pa2564 SWALL:TAM_PSEAE (SWALL:Q9I0S1) (275 aa) fasta scores: E(): 1.2e-11, 52.85% id in 70 aa. Note: This CDS lack both, start and stop codons	putative trans-aconitase (fragment)
BPSL2084A	Probable gene remnant. Similar to the C-terminal region of Ralstonia solanacearum ISRso14-transposase OrfB protein SWALL:Q8XF72 (EMBL:AL646083) (275 aa) fasta scores: E(): 2.3e-11, 43.61% id in 94 aa, and to Oligotropha carboxidovorans transposase B SWALL:Q6LB76 (EMBL:X82447) (241 aa) fasta scores: E(): 1.1e-12, 46.15% id in 104 aa	putaive transposase protein (fragment)
BPSL2089	Partial CDS. Similar to the C-terminal region of bacteriophage phiE125 gp27 SWALL:Q8W6S4 (EMBL:AF447491) (262 aa) fasta scores: E(): 2.5e-41, 82.22% id in 135 aa	putative phage protein (fragment)
BPSL2766a	Probable gene remnant. Similar to Shigella flexneri orf, partial conserved hypothetical protein sf2566 SWALL:Q83K41 (EMBL:AE015272) (138 aa) fasta scores: E(): 3.4e-12, 72.3% id in 65 aa, and to Escherichia coli putative IS110 transposase s0055 SWALL:Q8GA45 (EMBL:AJ488511) (398 aa) fasta scores: E(): 4.1e-12, 72.3% id in 65 aa	putative transposase (fragment)
BPSL2806a	Gene remnant. Similar to C-terminal reion of Salmonella typhimurium phosphomannomutase ManB or CpsG or RfbL SWALL:MANB_SALTY (SWALL:P26341) (456 aa) fasta scores: E(): 0.8, 30.55% id in 72 aa	phosphomannomutase (fragment)
BPSS0072A	Partial CDS. Similar to the C-terminal region of Ralstonia solanacearum hypothetical protein rsc3358 or rs02633 SWALL:Q8XU36 (EMBL:AL646074) (179 aa) fasta scores: E(): 0.00013, 46.51% id in 43 aa, and of Neurospora crassa histone H3 methyltransferase Dim-5 SWALL:Q8X225 (EMBL:AF419248) (318 aa) fasta scores: E(): 0.49, 37.5% id in 48 aa;conserved hypothetical protein (fragment)	pseudo
BPSS0084	Gene remnant. Similar to internal region of Burkholderia cepacia OpcP1 SWALL:Q45106 (EMBL:D63823) (361 aa) fasta scores: E(): 1.9e-05, 58.06% id in 31 aa, and Ralstonia solanacearum probable porin signal peptide protein rsc2108 or rs01496 SWALL:Q8XXK5 (EMBL:AL646068) (381 aa) fasta scores: E(): 0.00028, 61.29% id in 31 aa. Feature lacks translational stop and start codons;putative porin-related protein (fragment)	pseudo
BPSS0285A	Gene remnant. N-terminus is similar to the N-terminal region of Burkholderia pseudomallei outer membrane porin precursor Omp38 SWALL:Q7WZL2 (EMBL:AY312416) (374 aa) fasta scores: E(): 6.6e-05, 36.28% id in 113 aa. C-terminus is similar to the C-terminal region of Burkholderia pseudomallei outer membrane porin precursor Omp38 SWALL:Q7WZL2 (EMBL:AY312416) (374 aa) fasta scores: E(): 0.00059, 51.85% id in 54 aa. CDS contains frameshift and nonsense mutations and a deletion relative to the B. pseudomallei protein;putative outer membrane porin precursor (fragment)	pseudo
BPSS0393	Gene remnant. Similar to the N-terminal region of Bacteriophage phiE125 Gp70 SWALL:Q8W6N0 (EMBL:AF447491) (118 aa) fasta scores: E(): 1.5e-30, 89.87% id in 79 aa, and of bacteriophage SFV hypothetical 13.1 kDa protein ORF53 SWALL:Q8SBD7 (EMBL:U82619) (116 aa) fasta scores: E(): 4e-12, 49.33% id in 75 aa;putative bacteriophage-related protein (fragment)	pseudo

BPSS0402A	Probable gene remnant. Similar to the C-terminal region of Coxiella burnetii transposase for insertion sequence element IS1111a SWALL:TRA1_COXBU (SWALL:Q45968) (339 aa) fasta scores: E(): 4.9e-23, 54.54% id in 132 aa, and to Edwardsiella ictaluri RmpB-like protein SWALL:Q6GUC9 (EMBL:AY641982) (358 aa) fasta scores: E(): 7.4e-29, 59.39% id in 133 aa;transposase (fragment)	pseudo
BPSS0600	Similar to Rhizobium loti hypothetical protein mll4983 SWALL:Q98CV6 (EMBL:AP003005) (281 aa) fasta scores: E(): 7.3, 34.88% id in 86 aa. Possible gene remnant	hypothetical protein
BPSS0653	Probable gene remnant. Similar to the C-terminal regions of Pseudomonas aeruginosa transposase for transposon Tn501 TnpA SWALL:TNP5_PSEAE (SWALL:P06695) (988 aa) fasta scores: E(): 1.4e-08, 47.43% id in 78 aa, and to Pseudomonas putida Tn4653 transposase TnpA SWALL:Q8VMH0 (EMBL:AJ344068) (988 aa) fasta scores: E(): 5.6e-09, 48.68% id in 76 aa;transposase (fragment)	pseudo
BPSS0919A	Gene remnant. Similar to internal regions of Nitrosomonas europaea ATCC 19718 integrase, catalytic core SWALL:Q82T33 (EMBL:BX321863) (204 aa) fasta scores: E(): 3.4e-08, 84.21% id in 38 aa, and Bacteriophage phiE125 TnpB SWALL:Q8W6R2 (EMBL:AF447491) (284 aa) fasta scores: E(): 4.5e-08, 84.21% id in 38 aa;putative transposase (fragment)	pseudo
BPSS1185	Gene remnant. Similar to the C-terminal region of Escherichia coli shikimate transporter ShiA SWALL:SHIA_ECOLI (SWALL:P76350) (438 aa) fasta scores: E(): 3.2e-20, 40.65% id in 214 aa. CDS contains at least two frameshift mutations;putative shikimate transporter (fragment)	pseudo
BPSS1208	Similar to an internal region of Mycobacterium avium transposase subunit B SWALL:Q933U0 (EMBL:AF232829) (306 aa) fasta scores: E(): 2.4e-06, 31.2% id in 125 aa, and to Bradyrhizobium japonicum id60 rsbeta3 SWALL:Q9ANN7 (EMBL:AF322012) (211 aa) fasta scores: E(): 4.2e-29, 63.35% id in 131 aa;putative transposase (fragment)	Pfam match to entry PF00665 rve, Integrase core domain , score 19.2, E-value 1.5e-05 (pseudo)
BPSS1384a	Probable gene remnant. Similar to the C-terminal regions of Escherichia coli transposase for transposon Tn1721 TnpA SWALL:TNP9_ECOLI (SWALL:P51565) (988 aa) fasta scores: E(): 6.2e-13, 48.71% id in 117 aa, and Pseudomonas aeruginosa transposase for transposon Tn501 TnpA SWALL:TNP5_PSEAE (SWALL:P06695) (988 aa) fasta scores: E(): 6.2e-13, 48.71% id in 117 aa;transposase (fragment)	pseudo
BPSS1385A	Probable gene remnant. Similar to internal regions of Burkholderia cepacia putative transposase for insertion sequence element IS402 SWALL:T402_BURCE (SWALL:P24536) (211 aa) fasta scores: E(): 1.4e-05, 37.63% id in 93 aa, and to Alcaligenes eutrophus ISJp4 transposase TnpA SWALL:Q6UP96 (EMBL:AY365053) (262 aa) fasta scores: E(): 0.00013, 33.7% id in 89 aa;transposase (fragment)	pseudo
BPSS1449a	Probable gene remnant. Similar to the C-terminal regions of Bordetella pertussis hypothetical protein SWALL:Q7VSI5 (EMBL:BX640412) (134 aa) fasta scores: E(): 5.8e-16, 66.26% id in 83 aa, and to Bordetella parapertussis hypothetical protein SWALL:Q7WC61 (EMBL:BX640424) (134 aa) fasta scores: E(): 5.8e-16, 67.47% id in 83 aa;conserved hypothetical protein (fragment)	pseudo
BPSS1515	Partial CDS. Similar to an internal region of several transposases including Clostridium cellulolyticum transposase Tpna1 SWALL:AAN06001 (EMBL:AY130778) (348 aa) fasta scores: E(): 2.5e-19, 53% id in 100 aa, and to Escherichia coli putative integrase SWALL:CAD33770 (EMBL:AJ488511) (152 aa) fasta scores: E(): 8.5e-20, 52.38% id in 105 aa;putative transposase (fragment)	pseudo

BPSS1774A	Gene remnant. Similar to parts of Xanthomonas axonopodis phage-related tail protein S or xac2653 SWALL:Q8PJ88 (EMBL:AE011905) (148 aa) fasta scores: E(): 0.065, 43.18% id in 44 aa, and to Bacteriophage phi CTX ORF14 SWALL:Q9ZXL2 (EMBL:AB008550) (156 aa) fasta scores: E(): 0.5, 39.21% id in 51 aa;putative phage-related tail protein (fragment)	pseudo
BPSS2004	Probable gene remnant. Similar to the N-terminal regions of Ralstonia solanacearum ISRso16-transposase OrfB protein Rsp0558 or Rs03921 SWALL:Q8XSC0 (EMBL:AL646079) (280 aa) fasta scores: E(): 1.4e-09, 56.25% id in 64 aa, and Yersinia pestis putative transposase ypcd1.94 SWALL:Q9RI05 (EMBL:AL117189) (269 aa) fasta scores: E(): 3.5e-06, 48.43% id in 64 aa;putative IS element protein (fragment)	pseudo
BPSL0288A	Similar to Pseudomonas putida glyoxalase family protein SWALL:Q88H56 (EMBL:AE016787) (125 aa) fasta scores: E(): 5.4e-11, 41.52% id in 118 aa, and to Pseudomonas syringae pv. tomato str. DC3000 hypothetical protein SWALL:Q887D4 (EMBL:AE016860) (131 aa) fasta scores: E(): 1.5e-11, 42.99% id in 107 aa. CDS contains a nonsense mutation (amber) after codon 26, and frameshifts after codons 18, 67 and 91	conserved hypothetical protein (pseudogene)
BPSL1788	Pseudogene. Similar to Thermotoga maritima sugar kinase, FggY family tm0284 SWALL:Q9WYC0 (EMBL:AE001710) (506 aa) fasta scores: E(): 2.1e-90, 45.54% id in 505 aa, and to Yersinia pestis KIM putative xylulose kinase y2893 SWALL:AAM86444 (EMBL:AE013892) (517 aa) fasta scores: E(): 1.3e-38, 32.38% id in 528 aa. Contains a frameshift after residue 455	putative sugar kinase (pseudogene)
BPSL2402A	Weakly similar to the C-terminal region of Burkholderia glumae hypothetical protein SWALL:Q7X299 (EMBL:AB112549) (205 aa) fasta scores: E(): 1.5, 28.31% id in 166 aa. CDS contains a possible frameshift after codon 27. Without the framesift there is no available translational start site for this CDS	hypothetical protein (pseudogene)
BPSS0060	-	hypothetical protein
BPSS0268A	-	hypothetical protein
BPSS0379	flanked by integrase gene and repeats	hypothetical protein
BPSS0397A	-	hypothetical protein (36aa)
BPSS0398	-	hypothetical protein
BPSS0412	-	hypothetical protein
BPSS0480	-	hypothetical protein (41aa)
BPSS0629	-	hypothetical protein
BPSS1003	-	hypothetical protein
BPSS1026	-	hypothetical protein
BPSS1048b	-	hypothetical protein
BPSS1048c	-	hypothetical protein
BPSS1051	-	hypothetical protein
BPSS1876A	-	hypothetical protein
BPSS2122	-	hypothetical protein
BPSL1323a	Similar to Pseudomonas putida hypothetical protein SWALL:O68643 (EMBL:AF052749) (109 aa) fasta scores: E(): 2.2e-18, 48.57% id in 105 aa, and to Salmonella typhimurium putative cytoplasmic protein stm1261 SWALL:Q8ZPY1 (EMBL:AE008755) (108 aa) fasta scores: E(): 2.4e-19, 47.17% id in 106 aa	hypothetical protein

BPSL3260	Similar to Pseudomonas aeruginosa hypothetical protein Pa4674 SWALL:Q9HVC1 (EMBL:AE004881) (101 aa) fasta scores: E(): 0.00078, 35.86% id in 92 aa, and to Bacteroides nodosus virulence-associated protein A' VapA' SWALL:VAPZ_BACNO (SWALL:Q46561) (115 aa) fasta scores: E(): 0.0012, 35.29% id in 85 aa	hypothetical protein
BPSL0135	Similar to Ralstonia solanacearum hypothetical protein rsc1899 or rs03466 SWALL:Q8XY64 (EMBL:AL646067) (68 aa) fasta scores: E(): 2.2, 28.78% id in 66 aa	hypothetical protein
BPSL0557A	Similar to internal regions of Chlorobium tepidum DNA helicase, putative SWALL:Q8KC08 (EMBL:AE012917) (1510 aa) fasta scores: E(): 1.3e-08, 76.59% id in 47 aa, and to Clostridium acetobutylicum superfamily I DNA helicase SWALL:Q97ES1 (EMBL:AE007800) (1351 aa) fasta scores: E(): 0.94, 50% id in 46 aa	hypothetical protein
BPSL2042	Low similarity to Escherichia coli, and Escherichia coli O157:H7 cell division protein FtsL or MraR or b0083 or z0093 or ecs0087 SWALL:FTSL_ECOLI (SWALL:P22187) (121 aa) fasta scores: E(): 0.89, 33.33% id in 93 aa	hypothetical protein
BPSL0749	Similar to Escherichia coli O157:H7 hypothetical protein z0327 or ecs0292 SWALL:Q8X7K4 (EMBL:AE005203) (152 aa) fasta scores: E(): 6.8e-05, 22.44% id in 147 aa	hypothetical protein
BPSL0147	Similar to bacteriophage phi CTX Orf24.5 protein SWALL:Q9ZXK1 (EMBL:AB008550) (39 aa) fasta scores: E(): 1.9e-07, 56.75% id in 37 aa, and to Xanthomonas campestris pv. campestris str. ATCC 33913 phage-related protein orf52 SWALL:AAM42263 (EMBL:AE012413) (37 aa) fasta scores: E(): 4.4e-08, 62.16% id in 37 aa	hypothetical protein
BPSS1086a	Similar to Xanthomonas campestris phage-related protein Orf52 SWALL:Q8P6H8 (EMBL:AE012413) (37 aa) fasta scores: E(): 1.2e-07, 62.16% id in 37 aa, and to Bacteriophage phi CTX Orf24.5 SWALL:Q9ZXK1 (EMBL:AB008550) (39 aa) fasta scores: E(): 6.3e-07, 56.75% id in 37 aa	hypothetical protein
BPSL0161	Similar to bacteriophage phi CTX Orf12.5 protein SWALL:Q9ZXL4 (EMBL:AB008550) (89 aa) fasta scores: E(): 9.3e-09, 41.97% id in 81 aa, and to Salmonella typhimurium Fels-2 prophage: similar to protein from phage CTX STM2713 SWALL:Q8ZMT9 (EMBL:AE008823) (94 aa) fasta scores: E(): 6.2e-09, 43.59% id in 78 aa. Possible alternative translational start site	hypothetical protein
BPSS1054	Limited similarity to Ralstonia solanacearum putative bacteriophage transcriptional activator-related transcription regulator protein rsc0964 or rs04403 SWALL:Q8Y0S9 (EMBL:AL646062) (73 aa) fasta scores: E(): 0.0084, 36.17% id in 47 aa, and to Ralstonia solanacearum putative transcriptional activator transcription regulator protein rsc1904 or rs03470 SWALL:Q8XY59 (EMBL:AL646067) (82 aa) fasta scores: E(): 0.096, 40% id in 50 aa	bacteriophage-acquired protein
BPSS1518	Similar to N-terminal region of Xanthomonas oryzae putative transposase SWALL:Q93LQ4 (EMBL:AY035401) (344 aa) fasta scores: E(): 1.5e-07, 50% id in 66 aa	transposase
BPSS2148a	-	transposase

BPSL0947	N-terminus is similar to the C-terminal region of Escherichia coli type I restriction enzyme EcoEI specificity protein HsdS or Hss SWALL:T1SE_ECOLI (SWALL:P19705) (594 aa) fasta scores: E(): 6.4e-07, 27.55% id in 196 aa. Full length CDS is similar to Methanosarcina mazei type I restriction-modification system specificity subunit mm2703 SWALL:AAM32399 (EMBL:AE013517) (440 aa) fasta scores: E(): 5.9e-20, 37.64% id in 178 aa	putative type I restriction enzyme specificity protein
BPSS2062	Similar to Streptomyces coelicolor putative acetyltransferase SCO3363 or SCE94.14 SWALL:Q9X8M0 (EMBL:AL939116) (147 aa) fasta scores: E(): 1.6e-08, 38.73% id in 142 aa, and to the C-terminal region of Xanthomonas campestris acetyltransferase xcc4205 SWALL:Q8P373 (EMBL:AE012547) (203 aa) fasta scores: E(): 1.1e-05, 34.59% id in 159 aa	acetyltransferase (GNAT) family protein
BPSL1706	Similar to Ralstonia solanacearum putative HNS-like transcription regulator protein rsp0029 or rs02004 SWALL:Q8XTS5 (EMBL:AL646076) (95 aa) fasta scores: E(): 6.8e-07, 32.25% id in 93 aa, and to Escherichia coli ORF1 SWALL:O52122 (EMBL:AF022236) (129 aa) fasta scores: E(): 7.7e-05, 30.7% id in 114 aa	putative HNS-like protein
BPSS0383	Similar to Yersinia pestis putative regulatory protein ypo0878 or y3260 SWALL:Q8ZHL3 (EMBL:AJ414145) (66 aa) fasta scores: E(): 8.5e-05, 42% id in 50 aa, and to Vibrio cholerae transcriptional regulator vc1785 SWALL:Q9KR58 (EMBL:AE004255) (68 aa) fasta scores: E(): 8.8e-05, 37.03% id in 54 aa	DNA-binding protein
BPSL2255A	Similar to Neisseria gonorrhoeae outer membrane protein H.8 precursor SWALL:H82_NEIGO (SWALL:P11910) (88 aa) fasta scores: E(): 0.023, 43.9% id in 82 aa, and to Acinetobacter sp. hypothetical protein SWALL:Q6F875 (EMBL:CR543861) (88 aa) fasta scores: E(): 0.011, 47.56% id in 82 aa	putative lipoprotein
BPSSs01	TPP riboswitch (THI element) as predicted by Rfam (RF00059), score 61.81	misc_RNA
