# Supplementary material for: A Genomic Survey of Positive Selection in Burkholderia pseudomallei Provides Insights into the Evolution of Accidental Virulence
Source: PLoS Pathog. 2010 Apr 1;6(4):e1000845. doi: 10.1371/journal.ppat.1000845 (PMC2848565; doi:10.1371/journal.ppat.1000845)
Supplement: Table S5 — Recurrent SNPs and SNPs identified in five high-quality Bp genomes. ξSNP observed in at least two of eleven Bp genomes. *SNPs observed across 23 Bp genomes after removal of paralogous and non-shared loci [5]. †The five high sequence quality genomes are: B. pseudomallei K96243, B. pseudomallei 22, B. pseudomallei 1106a, B. pseudomallei 1710b, B. pseudomallei 668. (0.04 MB PDF) [file ppat.1000845.s013.pdf]

Table S5: Recurrent SNPs and SNPs identified in five high-quality Bp genomes

	Total no of SNPs across 11 genomes	Evidence 1			Evidence 2	Total Number of SNPs supported by either Evidence 1 or 2
		Recurrent SNP's			SNPs present across five high sequence quality Bp genomes[†]	
		11 Bp genomes[ξ]	23 Bp genomes*	Total		
Bp Chr1	15236	8002	2928	9637	11335	12750 (~84%)
Bp Chr2	11080	5624	1363	6359	8307	9029 (~82%)
