# Supplementary material for: A Genomic Survey of Positive Selection in Burkholderia pseudomallei Provides Insights into the Evolution of Accidental Virulence
Source: PLoS Pathog. 2010 Apr 1;6(4):e1000845. doi: 10.1371/journal.ppat.1000845 (PMC2848565; doi:10.1371/journal.ppat.1000845)
Supplement: Table S6 — Sequence Variations between Primary and Relapse Bp Strains. GeneID: Based on 1106a annotation; SNP a -> b: nucleotide changes; S/N: Synonymous vs Nonsynonymous alteration; BPCG+: Present in Bp core genome. (0.07 MB PDF) [file ppat.1000845.s014.pdf]

Table S6: Sequence Variations Between Primary and Relapse Bp Strains
1106a vs 1106b (top) and 1710a vs 1710b (bottom)

Chr	GeneID	Annotation	1106a Position	SNP a -> b	Presence in gene	S/N	Comments	Support
I	1106a_CHR1_734	sensor histidine kinase	797819	T->G	Yes	N	$BpCG^{+}$	**Real G** (10 strong reads)
I	Intergenic	-	1219136	C->T	No			**Real T** (6 strong reads)
I	1106a_CHR1_3055	UDP-N-acetylmuranoylalanine-D-glutamine ligase, murD	3457383	G->A	Yes	S	$BpCG^{+}$	**Real A** (10 strong reads)
I	1106a_CHR1_3513	Delta 1-pyrroline-5-carboxylate dehydrogenase	3936876	G->A	Yes	N	$BpCG^{+}$	**Real A** (7 strong reads)
II	1106a_CHR2_629	Hypothetical protein	759198	C->T	Yes	S	$BpCG^{+}$	**Real T** (5 strong reads)

Chr	GeneID	Annotation	1710a Position	SNP a -> b	Presence in gene	S/N	Comments	Support
I	1710a_CHR1_902	Not annotated	960721	G->T	Yes	N	$BpCG^{-}$	**Real G** (7 strong reads)
I	1710a_CHR1_2790	HrpA-like helicases	3095929	G->A	Yes	S	$BpCG^{+}$	**Real G** (5 reads)
I	1710a_CHR1_2928	Molybdenum cofactor biosynthesis enzyme	3245933 - 3245949	Various	Yes	-	$BpCG^{+}$	**Real Differences** (7 strong reads)
I	Intergenic	-	3340951	G->C	No			**Real G** (5 strong reads)
II	1710a_CHR2_448	Ribose/xylose/arabinose/galactoside ABC-type transport systems, permease components	533267	G->T	Yes	S	$BpCG^{+}$	**Real G** (2 strong reads)
II	1710a_CHR2_472	hypothetical protein	558833	C->A	Yes	S	$BpCG^{+}$	**Real C** (2 strong reads)
II	Intergenic	-	1124322 - 1124403	Various	No			**Real** (6 strong reads)
II	1710a_CHR2_1025	BsaU protein	1258749	T->G	Yes	N	$BpCG^{-}$	**Real** (4 strong reads)
