# Supplementary material for: A Genomic Survey of Positive Selection in Burkholderia pseudomallei Provides Insights into the Evolution of Accidental Virulence
Source: PLoS Pathog. 2010 Apr 1;6(4):e1000845. doi: 10.1371/journal.ppat.1000845 (PMC2848565; doi:10.1371/journal.ppat.1000845)
Supplement: Table S7 — List of Positively Selected Bp Genes (ranked by Ka/Ks). A) Chromosome 1. B) Chromosome 2. *Genes with a recombination signature. (0.14 MB PDF) [file ppat.1000845.s015.pdf]

Table S7: List of Positively Selected Bp Genes (ranked by K_a/K_s)

A) Chromosome 1

Gene ID	Start	Stop	Protein (aa)	Strand	Functional Annotation	K_a/K_s	P value
BPSL0320F	340129	341037	302	-	Sugar kinases, ribokinase family	999	2.87E-20
BPSL0464F	501260	501553	97	-	Uncharacterized protein conserved in bacteria	999	5.27E-10
BPSL0706.1	808385	808960	191	-	Hypothetical protein	999	1.25E-05
BPSL0814F	942889	944151	420	+	Membrane-fusion protein	999	1.80E-26
BPSL1304F	1519247	1520281	344	-	Transcriptional regulators	999	3.59E-07
BPSL1368F	1598976	1599419	147	-	Hypothetical protein {UniProtKB/TrEMBL	999	2.31E-21
BPSL1437F	1673453	1674487	344	+	ATPase involved in DNA replication	999	5.91E-51
BPSL1860F	2214022	2215164	380	-	Enoyl-CoA hydratase/carnithine racemase	999	1.74E-04
BPSL1994F	2376350	2378350	666	+	Sugar kinases, ribokinase family	999	1.78E-14
BPSL2008F	2394125	2395090	321	+	Transcriptional regulator	999	6.73E-14
BPSL2231F	2684134	2685423	429	-	Permeases of the major facilitator superfamily	999	2.86E-27
BPSL2306F	2780897	2782117	406	-	Nucleotidyltransferase/DNA polymerase involved in DNA repair	999	2.30E-05
BPSL2597F	3116156	3116968	270	-	Septum formation inhibitor	999	3.78E-09
BPSL2621F	3143636	3144160	174	+	Putative membrane protein	999	6.77E-09
BPSL2754F	3294488	3295270	260	-	Tfp pilus assembly protein PilW	999	3.15E-05
BPSL3148F	3747567	3748127	186	-	ABC-type transport system involved in resistance to organic solvents, periplasmic component	999	1.10E-14
BPSL3324F	3944534	3945568	344	+	3-oxoacyl-[acyl-carrier-protein] synthase III	999	1.96E-34
BPSL1239F	1423224	1424399	391	-	Aspartate/tyrosine/aromatic aminotransferase	862.22	2.52E-22
BPSL1795F	2135020	2135730	236	+	Hypothetical protein {UniProtKB/TrEMBL	810.48	9.41E-04
BPSL1881F	2240046	2241272	408	+	Permeases of the major facilitator superfamily	717.79	6.39E-05
BPSL2198F	2639117	2640052	311	+	Predicted esterase of the alpha-beta hydrolase superfamily	684.06	1.69E-20
BPSL2933F	3503247	3504245	332	+	Transcriptional regulators	627.87	3.66E-16
BPSL2832F	3385307	3386329	340	-	Transcriptional regulator of heat shock gene	591	1.05E-27
BPSL1087F	1258240	1260138	632	+	Molecular chaperone, HSP90 family	577.21	2.11E-45
BPSL0640F	725701	727278	525	+	Predicted unusual protein kinase	525.02	3.31E-47
BPSL1104F	1274538	1275410	290	-	Predicted NADH: ubiquinone oxidoreductase, subunit RnfB	501.43	8.95E-12
BPSL2397F	2893986	2894873	295	-	Uncharacterized protein conserved in bacteria	413.83	2.21E-04
BPSL1572F	1828156	1829058	300	+	Transcriptional regulator	408.48	1.15E-15
BPSL0405F	439501	440694	397	+	Predicted integral membrane protein	396.81	2.45E-07
BPSL0266F	279104	279325	73	-	RebB protein, putative {UniProtKB/TrEMBL	396.36	1.80E-04
BPSL0703F	801764	803647	627	-	Signal transduction histidine kinase	305.3	5.44E-43
BPSL1566F	1817818	1819722	634	-	Outer membrane protein	303.07	3.89E-21
BPSL2681F	3208351	3209760	469	-	ABC-type polysaccharide/polyol phosphate transport system, ATPase component	300.95	6.87E-43

BPSL2335F	2821100	2822527	475	-	Adenosylmethionine-8-amino-7-oxononanoate aminotransferase	297.7	8.64E-18
BPSL1767F	2082153	2083436	427	-	Mg-chelatase subunit ChlI	284.2	3.70E-17
BPSL1591F	1844497	1845597	366	+	Saccharopine dehydrogenase and related proteins	281.56	1.51E-30
BPSL2631F	3154437	3155996	519	+	3-polyprenyl-4-hydroxybenzoate decarboxylase and related decarboxylases	271.89	4.81E-30
BPSL1114F	1291364	1292812	482	+	Superfamily II DNA and RNA helicases	265.97	7.38E-40
BPSL1901F	2261328	2262917	529	+	Putative membrane protein	243.72	4.36E-07
BPSL2986F	3559364	3560773	469	+	Putative membrane protein	222.23	2.34E-07
BPSL0498F	541384	543978	864	+	Phosphoenolpyruvate-protein kinase (PTS system EI component in bacteria)	195.66	1.15E-18
BPSL1619F	1877519	1878418	299	+	Alcohol dehydrogenase, class IV	186.66	3.00E-13
BPSL2770F	3307838	3308794	318	-	Predicted sugar phosphate isomerase involved in capsule formation	175.4	8.39E-11
BPSL1457F	1693317	1694507	396	+	Predicted transmembrane transcriptional regulator (anti-sigma factor)	161.89	8.83E-16
BPSL3337F	3957433	3958275	280	+	Predicted hydrolases or acyltransferases (alpha/beta hydrolase superfamily)	142.41	3.34E-11
BPSL0719F	822429	825545	1038	-	Membrane carboxypeptidase (penicillin-binding protein)	140.56	1.25E-05
BPSL3081F	3675933	3676538	201	-	Nitroreductase	128.98	1.53E-04
BPSL3040F	3629441	3630217	258	-	Enoyl-CoA hydratase/carnithine racemase	121.71	6.88E-04
BPSL2956F	3528052	3528819	255	+	Uncharacterized protein conserved in bacteria	120.22	2.97E-06
BPSL0709F	811870	812583	237	+	Transcriptional regulator	116.83	1.38E-07
BPSL1614F	1870831	1871871	346	-	Phenylpropionate dioxygenase and related ring-hydroxylating dioxygenases, large terminal subunit	116.82	2.73E-04
BPSL2533F	3052469	3054313	614	+	FOG: TPR repeat	114.19	5.19E-04
BPSL2138F	2565179	2566126	315	-	Membrane protease subunits, stomatin/prohibitin homologs	101.79	8.70E-06
BPSL0602F	676718	679045	775	+	FOG: EAL domain	96.09	3.90E-04
BPSL3229F	3836722	3837999	425	-	Lysophospholipase L1 and related esterases	94.42	3.96E-23
BPSL1379F	1608253	1608603	116	-	Uncharacterized conserved small protein	87.05	4.09E-04
BPSL2048AF	2449974	2450261	95	-	Hypothetical protein {UniProtKB/TrEMBL	70.64	2.04E-05
BPSL1057F1	1224673	1224987	104	-	Hypothetical protein	62.27	8.38E-04
BPSL2694F	3220452	3221783	443	-	Hypothetical protein {UniProtKB/TrEMBL	60.07	1.14E-04
BPSL0610F	688607	690583	658	-	Hypothetical protein {UniProtKB/TrEMBL	58.23	8.52E-13
BPSL1127F	1310507	1311775	422	-	Permeases of the major facilitator superfamily	55.22	4.67E-13
BPSL0667F	757548	759647	699	-	Glycyl-tRNA synthetase, beta subunit	46.79	1.04E-04
BPSL1852F	2206874	2207845	323	+	tRNA-dihydrouridine synthase	42.33	2.03E-05
BPSL2324F	2806338	2810465	1375	-	HrpA-like helicases	41.32	2.53E-05
BPSL0977F	1135814	1136815	333	+	ABC-type Fe3+-siderophore transport system, permease component	41.23	8.03E-10
BPSL1369F	1599935	1601008	357	+	Membrane-associated phospholipid phosphatase	39.37	5.57E-05
BPSL2015F	2404072	2406096	674	-	Beta-glucosidase-related glycosidases	36.39	7.13E-07

BPSL1263F	1458136	1459971	611	-	Predicted signal transduction protein containing a membrane domain, an EAL and a GGDEF domain	34.74	3.42E-06
BPSL3029F	3610071	3611444	457	-	UDP-N-acetylmuramyl pentapeptide synthase	33.88	8.67E-08
BPSL0608F	685724	686419	231	+	PAP2 superfamily {UniProtKB/TrEMBL	33.1	3.96E-11
BPSL3041F	3630348	3632054	568	+	NAD-dependent aldehyde dehydrogenases	30.97	9.52E-06
BPSL2377F	2872233	2872658	141	-	Hemerythrin	28.05	6.01E-04
BPSL2755F	3295270	3295680	136	-	Putative exported protein	27.38	7.67E-04
BPSL0019F	20480	22135	551	-	Outer membrane protein	24.95	4.43E-10
BPSL0303F	321837	322511	224	+	Uncharacterized conserved protein	24.61	3.25E-04
BPSL2084F	2503503	2505452	649	-	O-Methyltransferase involved in polyketide biosynthesis	23.87	2.90E-04
BPSL1918F	2285354	2288281	975	-	Translation initiation factor 2 (IF-2; GTPase)	22.92	3.58E-09
BPSL2074F	2482474	2485884	1136	+	Glycosidases	22.35	1.78E-05
BPSL0508F	558769	559590	273	-	Histone acetyltransferase HPA2 and related acetyltransferases	22.18	7.57E-04
BPSL3378F	4008608	4011118	836	-	Cation transport ATPase	22.04	2.29E-06
BPSL0103F	114323	115771	482	-	DNA-directed RNA polymerase, sigma subunit (sigma70/sigma32)	21.18	3.20E-07
BPSL2479F	2993401	2993904	167	+	Uncharacterized protein conserved in bacteria	20.84	6.44E-05
BPSL0987F	1144744	1146333	529	+	Cobyric acid synthase	20.12	9.51E-07
BPSL2041F	2438558	2441398	946	-	Uncharacterized protein conserved in bacteria	19.51	2.33E-16
BPSL1812F	2159206	2160171	321	-	Flp pilus assembly protein TadD, contains TPR repeats	18.83	2.35E-18
BPSL1179F	1360450	1363092	880	+	Predicted ABC-type transport system involved in lysophospholipase L1 biosynthesis, permease component	18.38	5.17E-12
BPSL0837F	972462	973673	403	+	Arabinose efflux permease	17.35	2.34E-06
BPSL0706F	806553	808304	583	-	Methyl-accepting chemotaxis protein	17.1	2.81E-10
BPSL0388F	421177	422496	439	-	Putative exported protein	16.79	1.57E-04
BPSL1347F	1572506	1573639	377	-	Hypothetical protein {UniProtKB/TrEMBL	16.05	3.35E-12
BPSL2362F	2854683	2855717	344	-	Collagenase and related proteases	14.87	1.97E-06
BPSL2007F	2392284	2393978	564	-	Permeases of the major facilitator superfamily	14.56	3.51E-22
BPSL0118F	131843	134563	906	-	Topoisomerase IA	14.38	1.74E-05
BPSL1416F	1649862	1653932	1356	+	Phosphoribosylformylglycinamidine (FGAM) synthase, synthetase domain	13.2	8.96E-09
BPSL2092F	2511975	2513438	487	-	Uncharacterized protein conserved in bacteria	12.32	8.04E-04
BPSL2100.1	2522924	2523502	192	+	Hypothetical protein	12.11	3.22E-05
BPSL0492F	533334	534443	369	-	Hypothetical protein {UniProtKB/TrEMBL	11.76	7.66E-04
BPSL2363F	2855806	2857023	405	-	Dioxygenases related to 2-nitropropane dioxygenase	11.16	1.88E-04
BPSL1628F	1885085	1887637	850	+	P pilus assembly protein, porin PapC	10.86	2.21E-06
BPSL3097F	3697190	3701083	1297	+	Uncharacterized protein conserved in bacteria	10.86	1.59E-04
BPSL2773F	3310355	3311437	360	-	Glycosyltransferase	10.76	6.89E-04
BPSL2718F	3252188	3253627	479	+	TRAP-type uncharacterized transport system, periplasmic component	10.45	1.40E-04

BPSL1952F	2323708	2324808	366	+	Predicted xylanase/chitin deacetylase	8.35	1.19E-04
BPSL0892a.1	1038841	1039299	152	+	Hypothetical protein	7.88	4.09E-05
BPSL3386F	4017014	4018654	546	+	Acyl-CoA dehydrogenases	7.67	3.28E-04
BPSL0321F	341034	342491	485	-	N-acyl-D-glucosamine 2-epimerase	6.77	2.01E-04
BPSL3413F	4053013	4054158	381	-	ABC-type branched-chain amino acid transport systems, periplasmic component	5.49	3.32E-12
BPSL0701F	799353	799808	151	-	Putative exported protein	5.4	1.81E-04
BPSL0579F	641119	642699	526	+	Hypothetical protein {UniProtKB/TrEMBL	4.35	6.21E-06

B) Chromosome 2

Gene ID	*Start*	*Stop*	*Protein (aa)*	*Strand*	*Functional Annotation*	K_a/K_s	*P value*
BPSS0483F	655476	656603	375	+	3-oxoacyl-[acyl-carrier-protein] synthase III	999	9.10E-06
BPSS0559F	763153	764025	290	-	Transcriptional regulator	999	5.46E-06
BPSS0875F	1168399	1169010	203	-	Putative threonine efflux protein	999	1.58E-24
BPSS1561.1	2119909	2120361	150	-	hypothetical protein	999	4.46E-27
BPSS1732F	2379008	2379640	210	-	Putative membrane protein	999	5.82E-05
BPSS1998.1	2705881	2706084	67	+	Hypothetical protein	999	5.77E-04
BPSS1474F	2011759	2012517	252	-	Transcriptional regulators of sugar metabolism	998.27	1.20E-25
BPSS0190F	254427	255722	431	-	O-acetylhomoserine sulfhydrylase	930.54	7.66E-45
BPSS0651F	883744	884112	122	+	Regulator of competence-specific genes	637.4	1.45E-06
BPSS1684F	2314235	2316697	820	-	Glycosyltransferase	617.55	4.37E-36
BPSS1318F	1805789	1807282	497	-	FAD/FMN-containing dehydrogenases	473.38	6.49E-18
BPSS0227F	307560	311486	1308	+	Putative membrane protein	415.38	2.46E-06
BPSS2130F	2882057	2884420	787	-	Acyl-CoA synthetase (NDP forming)	415.28	4.27E-66
BPSS1020F	1393691	1394704	337	-	Response regulator containing CheY-like receiver domain and AraC-type DNA-binding domain	415.01	2.38E-12
BPSS1973F	2665124	2667058	644	+	Predicted protease	412.25	6.73E-32
BPSS1570F	2131287	2131895	202	+	N-acyl-L-homoserine lactone synthetase	325.58	6.36E-15
BPSS1475F	2013143	2014453	436	-	Permeases of the major facilitator superfamily	316.03	4.24E-12
BPSS1552F	2105136	2105918	260	+	Type III secretion system protein	302.8	2.93E-21
BPSS1602F	2177487	2178593	368	+	Tfp pilus assembly protein, pilus retraction ATPase PilT	270.85	1.77E-26
BPSS1877F	2551038	2551319	93	+	Hypothetical protein {UniProtKB/TrEMBL:Q62E38}	267.63	3.09E-04
BPSS1580F	2145560	2149621	1353	-	FOG: TPR repeat	258.37	6.80E-09
BPSS1251F	1695207	1696586	459	-	Acetylornithine deacetylase/Succinyl-diaminopimelate desuccinylase and related deacylases	229.83	7.66E-15
BPSS2125F	2875646	2876725	359	+	L-lactate dehydrogenase (FMN-dependent) and related alpha-hydroxy acid dehydrogenases	209.6	7.57E-19

BPSS0460F	626716	628539	607	+	Methyl-accepting chemotaxis protein	205.37	5.00E-04
BPSS0377F	516820	518835	671	+	Predicted symporter	202.12	3.92E-06
BPSS0452F	618584	620524	646	+	DNA polymerase IV (family X)	181.41	4.34E-50
BPSS0805F	1077778	1079844	688	-	FOG: EAL domain	164.19	7.95E-38
BPSS0752F	1010507	1012153	548	-	Putative lipoprotein {UniProtKB/TrEMBL:Q63MA8}	161.55	1.05E-08
BPSS1809F	2468155	2468997	280	-	Predicted thioesterase involved in non-ribosomal peptide biosynthesis	155.44	9.33E-04
BPSS1094F	1466788	1467363	191	+	hypothetical protein	152.97	2.50E-11
BPSS1637F	2255243	2256574	443	-	Acetyl-CoA hydrolase	137.66	5.36E-32
BPSS0059.1	64202	64465	87	-	Hypothetical protein	137.54	1.01E-07
BPSS1384F	1891215	1892447	410	-	Hypothetical protein	123.76	1.64E-04
BPSS1974F	2667859	2669577	572	+	Putative lipoprotein {UniProtKB/TrEMBL:Q63IU2}	121.11	6.70E-18
BPSS1293F	1771907	1772497	196	+	Putative lipoprotein {UniProtKB/TrEMBL:Q63KR9}	119.55	1.52E-12
BPSS0940F	1240463	1241416	317	+	Predicted ornithine cyclodeaminase, mu-crystallin homolog	118.83	1.02E-07
BPSS2132F	2887571	2889091	506	-	Transcriptional regulator containing PAS, AAA-type ATPase, and DNA-binding domains	109.33	1.63E-14
BPSS0994F	1312677	1314146	489	-	SAM-dependent methyltransferases	98.83	1.42E-07
BPSS1326F	1814914	1816338	474	+	Uncharacterized conserved protein	98.2	1.86E-06
BPSS1960F	2648584	2649906	440	-	Thymidine phosphorylase	85.26	1.56E-16
BPSS1394F	1908508	1909839	443	-	Flagellar biosynthesis/type III secretory pathway ATPase	82.9	6.78E-24
BPSS2145F	2903723	2905414	563	-	Exopolysaccharide biosynthesis protein related to N-acetylglucosamine-1-phosphodiester alpha-N-acetylglucosaminidase	82.45	4.35E-04
BPSS1031F	1404343	1405428	361	-	Ribose/xylose/arabinose/galactoside ABC-type transport systems, permease components	80.22	1.12E-10
BPSS1272F	1735704	1737497	597	-	Acyl-CoA dehydrogenases	77.94	1.15E-06
BPSS0995F	1314324	1315883	519	-	AraC-type DNA-binding domain-containing proteins	64.13	5.97E-04
BPSS0759F	1019550	1020812	420	+	Purine-cytosine permease and related proteins	62.07	4.65E-06
BPSS2248F	3021202	3022149	315	+	Predicted glycosyltransferases	61.74	1.77E-05
BPSS0161F	212650	213564	304	+	Probable taurine catabolism dioxygenase	57.64	7.48E-04
BPSS0085F	98817	99356	179	-	Integral membrane protein	54.93	4.77E-08
BPSS0054.1	54051	54548	165	-	hypothetical protein	50.79	3.82E-07
BPSS0893F	1184982	1186175	397	-	Outer membrane protein (porin)	48.72	5.81E-12
BPSS1503F	2049856	2052879	1007	+	Uncharacterized protein conserved in bacteria	46.81	3.25E-04
BPSS0776F	1036929	1037864	311	+	Gluconolactonase	46.04	6.30E-10
BPSS0787F	1052730	1054286	518	+	ABC-type sugar transport system, ATPase component	41.55	2.06E-11
BPSS0023F	22139	23317	392	+	Cytochrome P450	39.72	6.68E-04
BPSS1754F	2404782	2405999	405	+	Predicted acyltransferases	39.27	2.27E-05
BPSS1491F	2032361	2033761	466	+	hypothetical protein	38.82	3.03E-11
BPSS0750F	1007935	1009344	469	+	Amino acid transporters	37.04	5.05E-10

BPSS1520F	2072125	2073117	330	-	AraC-type DNA-binding domain-containing proteins	33.97	1.93E-05
BPSS0283F	378724	380277	517	-	Predicted membrane protein	33.89	9.68E-06
BPSS0117F	147309	150353	1014	-	Signal transduction histidine kinase	32.67	6.13E-06
BPSS0945F	1247073	1248020	315	+	Membrane proteins related to metalloendopeptidases	30.68	6.09E-06
BPSS0147F	192341	193654	437	-	Permeases of the major facilitator superfamily	29.77	1.68E-14
BPSS1010F	1379414	1381072	552	+	Putative halogenase {UniProtKB/TrEMBL:Q63LK5}	29.58	3.07E-15
BPSS2115F	2862875	2863789	304	+	Transcriptional regulator	28.11	2.31E-04
BPSS0151F	197308	198459	383	+	Fatty acid desaturase	27.17	7.59E-04
BPSS1654F	2272681	2275035	784	-	Cytochrome P450	26.53	4.93E-04
BPSS0795.2	1062233	1062445	70	-	No Hits Found	25.06	2.20E-05
BPSS1265F	1715962	1716288	108	+	Hypothetical protein {UniProtKB/TrEMBL:Q63KU9}	24.88	9.83E-05
BPSS0096F	115675	117351	558	+	Outer membrane protein and related peptidoglycan-associated (lipo)proteins	21.34	2.16E-06
BPSS2102F	2843816	2846431	871	-	Serine/threonine protein kinase	20.72	1.07E-06
BPSS0736F	989669	990994	441	-	hypothetical protein	19.82	2.54E-04
BPSS0799F	1071034	1072188	384	-	FOG: EAL domain	19.37	3.27E-04
BPSS0128F	164175	165629	484	+	Pyruvate kinase	19.14	4.27E-12
BPSS1454F	1984227	1985591	454	+	Hypothetical protein {UniProtKB/TrEMBL:Q62CS0}	17.23	9.55E-04
BPSS1686F	2318209	2320530	773	-	Putative exported protein	16.35	4.92E-05
BPSS0027F	25519	27462	647	+	Fe-S oxidoreductase	14.78	2.82E-09
BPSS1403F	1917520	1918806	428	+	Flagellar motor switch/type III secretory pathway protein	14.2	3.56E-04
BPSS0587F	800407	806454	2015	+	Non-ribosomal peptide synthetase modules and related proteins	14.16	1.37E-51
BPSS1850F	2512421	2514466	681	-	Outer membrane receptor proteins, mostly Fe transport	13.63	7.51E-06
BPSS0468F	637724	639142	472	-	Adenosylmethionine-8-amino-7-oxononanoate aminotransferase	13.45	8.71E-04
BPSS0180F	240376	241455	359	+	Uncharacterized protein conserved in bacteria	13.44	2.18E-10
BPSS0095F	114983	115672	229	+	Putative membrane protein	13	5.13E-06
BPSS1504F	2052905	2055547	880	+	Uncharacterized low-complexity proteins	12.93	6.64E-06
BPSS0443F	607554	608903	449	+	hypothetical protein	12.38	1.89E-04
BPSS0087F[*]	102280	103737	485	-	Putative membrane protein	12.31	4.07E-04
BPSS2095F	2836073	2837518	481	-	Uncharacterized protein conserved in bacteria	12.21	2.62E-05
BPSS2328F	3134787	3141578	2263	-	Polyketide synthase modules and related proteins	12.08	1.34E-11
BPSS1938F[*]	2621133	2624357	1074	+	ABC-type multidrug transport system, ATPase component	11.91	2.22E-05
BPSS2103F	2846642	2847583	313	-	Uncharacterized protein conserved in bacteria	11.73	2.00E-04
BPSS2163F	2926408	2927301	297	-	Predicted permeases	11.42	2.95E-05
BPSS1371F	1876037	1877203	388	+	Transcriptional regulator containing an amidase domain and an AraC-type DNA-binding HTH	10.97	9.44E-07

					domain		
BPSS1790F	2448563	2450485	640	-	Uncharacterized conserved protein	9.68	1.29E-04
BPSS1502F	2046791	2049829	1012	+	ATPases with chaperone activity, ATP-binding subunit	9.36	7.87E-25
BPSS0443.1	608942	609427	161	-	No Hits Found	9.1	9.35E-09
BPSS1439F*	1960277	1964869	1530	+	Autotransporter adhesin	8.76	3.71E-06
BPSS0415F	570903	573890	995	-	Putative lipoprotein {UniProtKB/TrEMBL:Q63N81}	8.25	2.11E-06
BPSS2304F	3100875	3103451	858	-	Membrane carboxypeptidase/penicillin-binding protein	7.43	5.45E-04
BPSS1493F	2036034	2037986	650	-	Uncharacterized protein conserved in bacteria	6.67	1.95E-06
BPSS1727F	2368050	2370755	901	-	Large exoproteins involved in heme utilization or adhesion	5.63	4.40E-04
BPSS0146F	191677	192306	209	-	Outer membrane protein (porin)	5.51	4.55E-05
BPSS0086.1	101534	102004	156	+	No Hits Found	4.5	2.98E-06
