# Supplementary material for: A Genomic Survey of Positive Selection in Burkholderia pseudomallei Provides Insights into the Evolution of Accidental Virulence
Source: PLoS Pathog. 2010 Apr 1;6(4):e1000845. doi: 10.1371/journal.ppat.1000845 (PMC2848565; doi:10.1371/journal.ppat.1000845)
Supplement: Table S8 — List of selected gene candidates for transfection (0.06 MB PDF) [file ppat.1000845.s016.pdf]

Table S8: List of selected gene candidates for transfection

Sanger ID	Start	Stop	Strand	Functional Annotation	K_a/K_s	P value	Present
BPSL0492	533334	534443	-	Hypothetical protein	11.76	7.66E-04	BP-BM not in BT
BPSL0837	972462	973673	+	Arabinose efflux permease	17.35	2.34E-06	BP-BM not in BT
BPSL1057F1	1224673	1224987	-	Hypothetical protein	62.27	8.38E-04	BP-BM not in BT
BPSL2100.1	2522924	2523502	+	Hypothetical protein	12.11	3.22E-05	BP-BM not in BT
BPSL2755	3295270	3295680	-	Putative exported protein	27.38	7.67E-04	BP-BM not in BT/Virulence
BPSS0180	240376	241455	+	Uncharacterized protein conserved in bacteria	13.44	2.18E-10	BP-BM not in BT
BPSS0415	570903	573890	-	Putative lipoprotein	8.25	2.11E-06	BP-BT not in BM
BPSS0483	655476	656603	+	3-oxoacyl-[acyl-carrier-protein] synthase III	999.00	9.10E-06	Virulence
BPSS1010	1379414	1381072	+	Putative halogenase	29.58	3.07E-15	BP-BM not in BT /Virulence
BPSS1552	2105136	2105918	+	Type III secretion system protein	302.8	2.93E-21	Virulence
