# Supplementary material for: A Genomic Survey of Positive Selection in Burkholderia pseudomallei Provides Insights into the Evolution of Accidental Virulence
Source: PLoS Pathog. 2010 Apr 1;6(4):e1000845. doi: 10.1371/journal.ppat.1000845 (PMC2848565; doi:10.1371/journal.ppat.1000845)
Supplement: Table S9 — A) List of taurine regulated genes in Bp K96243 Chr 1 (up regulated > = 2 fold). B) List of taurine regulated genes in Bp K96243 Chr 2 (up regulated > = 2 fold). (0.09 MB PDF) [file ppat.1000845.s017.pdf]

Table S9A: List of taurine regulated genes in Bp K96243 Chr 1 (up regulated >=2 fold)

Cluster no	GENE ID	Start	Stop	Strand	Function
1	BPSL0024	26223	26621	+	LrgA family protein
	BPSL0025	26690	27412	+	hypothetical protein
	BPSL0026	28250	28747	+	flagellar basal body protein
	BPSL0027	28770	29768	+	flagellar motor switch protein
	BPSL0028	29761	30258	+	probable flagellar motor switch protein
	BPSL0029	30426	30920	+	flagellar protein
	BPSL0030	30991	31752	+	flagellar biosynthetic protein
	BPSL0031	31778	32050	+	flagellar biosynthesis protein
	BPSL0032	32169	32951	+	flagellar biosynthetic protein
2	BPSL0224	233741	235384	+	putative GMC oxidoreductase
	BPSL0225	235424	236833	-	putative flagellar hook-length control protein
	BPSL0226	236866	237318	-	flagellar fliJ protein
	BPSL0227	237324	238895	-	flagellum-specific ATP synthase
	BPSL0228	238889	239569	-	flagellar assembly protein
	BPSL0229	239562	240557	-	flagellar motor protein
	BPSL0230	240547	242343	-	flagellar M-ring protein
	BPSL0231	242591	242932	+	flagellar hook-basal body complex protein
	BPSL0232	243065	243499	+	flagellar protein
	BPSL0233	243496	243822	+	hypothetical protein
	BPSL0234	243932	245443	+	hypothetical protein
	BPSL0235	245440	245757	+	putative export system protein
	BPSL0236	245950	246696	+	hypothetical protein
3	BPSL0266	279104	279325	-	hypothetical protein
	BPSL0267	279820	280260	-	putative flagella synthesis protein
	BPSL0268	280340	280684	-	putative negative regulator of flagellin synthesis
	BPSL0269	280788	281711	-	flagellar basal body P-ring biosynthesis protein
	BPSL0270	282577	283068	+	putative flagellar basal-body rod protein
	BPSL0271	283152	283577	+	flagellar basal body rod protein
	BPSL0272	283697	284482	+	putative basal-body rod modification protein
	BPSL0273	284510	285751	+	flagellar hook protein
	BPSL0274	285778	286539	+	flagellar basal-body rod protein
	BPSL0275	286573	287361	+	flagellar basal-body rod protein
	BPSL0276	287382	288104	+	flagellar L-ring protein precursor
	BPSL0277	288119	289303	+	flagellar P-ring protein precursor
	BPSL0278	289304	290239	+	putative peptidoglycan hydrolase
	BPSL0279	290455	291213	+	hypothetical protein

	BPSL0280	291419	293422	+	flagellar hook-associated protein
	BPSL0281	293438	294670	+	putative flagellar hook-associated protein
	BPSL0282	295038	296345	+	putative permease
4	BPSL0374	404374	405453	+	metallo-beta-lactamase superfamily protein
5	BPSL0472	509901	513119	+	DNA polymerase III subunit alpha
	BPSL0473	513457	514278	-	hypothetical protein
	BPSL0474	514322	515515	-	putative transporter protein
	BPSL0475	515717	516748	-	putative fatty acid desaturase
	BPSL0476	516794	518146	-	putative diaminobutyrate--2-oxoglutarate aminotransferase
	BPSL0477	518191	518724	-	hypothetical protein
	BPSL0478	518799	519890	-	hypothetical protein
	BPSL0479	519883	520839	-	ABC transport system ATP-binding protein
	BPSL0480	520932	521999	-	hypothetical protein
	BPSL0481	521996	522661	-	hypothetical protein
	BPSL0482	522701	523852	-	hypothetical protein
	BPSL0483	523854	525725	-	hypothetical protein
	BPSL0484	525722	526762	-	hypothetical protein
	BPSL0485	526780	528348	-	putative AMP-binding enzyme
	BPSL0486	528356	529552	-	putative pyridoxal-dependent decarboxylase
	BPSL0487	529542	530540	-	hypothetical protein
	BPSL0488	530545	531486	-	hypothetical protein
	BPSL0489	531547	532113	-	hypothetical protein
	BPSL0490	532110	532997	-	hypothetical protein
	BPSL0491	533030	533278	-	putative acyl carrier protein
	BPSL0492	533334	534527	-	hypothetical protein
	BPSL0493	534524	536290	-	putative AMP-binding enzyme
6	BPSL0602	676682	679045	+	hypothetical protein
	BPSL0603	679223	680491	-	putative polysaccharide biosynthesis protein
	BPSL0604	680568	681764	-	putative glycosyl transferase
	BPSL0605	681799	683391	-	putative mannose-1-phosphate guanylyltransferase
	BPSL0606	683388	684038	-	hypothetical protein
	BPSL0607	684416	685657	+	putative glycosyl transferase
	BPSL0608	685685	686419	+	PAP2 superfamily
	BPSL0609	687164	688552	+	fis family regulatory protein
	BPSL0610	688607	690583	-	hypothetical protein
	BPSL0611	690580	693099	-	glycosyl hydrolase family protein

	BPSL0612	693096	694184	-	hypothetical protein
	BPSL0613	694181	695155	-	hypothetical protein
	BPSL0614	695152	696375	-	putative acyl-CoA dehydrogenase
	BPSL0615	696372	696674	-	acyl carrier protein
	BPSL0616	696875	697669	-	hypothetical protein
	BPSL0617	698536	699402	+	hypothetical protein
	BPSL0618	699399	700817	+	putative sugar transferase
	BPSL0619	700889	702178	+	putative polysaccharide biosynthesis/export protein
	BPSL0620	702458	703603	+	glycosyl transferase group 1 protein
	BPSL0621	703632	704531	-	hypothetical protein
7	BPSL0814	942889	944151	+	putative RND family acriflavine resistance protein A precursor
	BPSL0815	944167	947367	+	putative RND family acriflavine resistance protein
	BPSL0816	947371	948915	+	outer membrane efflux protein
8	BPSL1771	2091314	2091766	+	cobalamin biosynthesis protein CbiG
	BPSL1772	2091825	2092427	+	cob(I)yrinic acid a,c-diamide adenosyltransferase
	BPSL1773	2092432	2094039	+	cobyrinic acid A,C-diamide synthase
	BPSL1774	2094346	2095185	-	putative siderophore biosynthesis related protein
	BPSL1775	2095250	2097514	-	putative iron uptake receptor precursor
	BPSL1776	2097511	2098917	-	putative L-ornithine 5-monooxygenase
	BPSL1777	2099061	2104307	-	putative siderophore-related non-ribosomal peptide synthase
	BPSL1778	2104329	2114201	-	putative siderophore related no-ribosomal peptide synthase
	BPSL1779	2114773	2116518	+	putative siderophore biosynthesis related ABC transport protein
	BPSL1780	2117026	2117601	+	hypothetical protein
	BPSL1781	2117793	2118821	-	putative iron transport-related exported protein
	BPSL1782	2118986	2119792	-	putative iron transport-related membrane protein
	BPSL1783	2119789	2121909	-	putative iron transport-related membrane protein
	BPSL1784	2121906	2122796	-	putative iron transport-related ATP-bidning protein
	BPSL1785	2122834	2123850	-	hypothetical protein
	BPSL1786	2123909	2124151	-	hypothetical protein
	BPSL1787	2124207	2124830	-	RNA polymerase sigma-70 factor

9	BPSL2026	2420543	2421508	-	putative lipoprotein
	BPSL2027	2421524	2423872	-	putative fimbriae-related protein
	BPSL2028	2423980	2424819	-	putative fimbriae-assembly chaperone
	BPSL2029	2424837	2425361	-	hypothetical protein
	BPSL2030	2425438	2425998	-	hypothetical protein
	BPSL2031	2426051	2426596	-	hypothetical protein
10	BPSL2468	2973705	2975111	+	putative multidrug resistance protein
11	BPSL3087	3682680	3683882	-	putative acyltransferase
	BPSL3088	3684236	3684682	-	hypothetical protein
	BPSL3089	3685343	3687508	+	family M1 unassigned peptidase
	BPSL3090	3688531	3688800	+	hypothetical protein
	BPSL3091	3688903	3689133	+	hypothetical protein
	BPSL3092	3689749	3691017	+	putative bacteriocin secretion protein
	BPSL3093	3691032	3693293	+	colicin V processing peptidase
	BPSL3094	3693290	3694738	+	putative outer membrane bacteriocin efflux protein
12	BPSL3288	3904907	3905737	-	5,10-methylenetetrahydrofolate reductase
	BPSL3289	3905755	3906108	-	hypothetical protein
	BPSL3290	3906272	3907693	-	S-adenosyl-L-homocysteine hydrolase
	BPSL3291	3908047	3908778	-	flagellar biosynthesis sigma factor FliA
	BPSL3292	3908803	3909615	-	flagellar biosynthesis protein FlhG
	BPSL3293	3909608	3911359	-	flagellar biosynthesis protein
	BPSL3294	3911356	3913458	-	flagellar biosynthesis protein
	BPSL3295	3913455	3914672	-	flagellar biosynthetic protein FlhB
	BPSL3296	3915368	3915868	-	hypothetical protein
	BPSL3297	3916055	3917359	-	Gly/Ala/Ser-rich lipoprotein
	BPSL3298	3917382	3917885	-	hypothetical protein
	BPSL3299	3918083	3918811	-	chemotaxis protein CheZ
	BPSL3300	3918813	3919241	-	chemotaxis protein CheY
	BPSL3301	3919557	3920660	-	chemotaxis-specific methylesterase
	BPSL3302	3920657	3921361	-	putative chemotaxis protein
	BPSL3303	3921358	3922305	-	chemotaxis protein methyltransferase
	BPSL3304	3922309	3924330	-	methyl-accepting chemotaxis protein I
	BPSL3305	3924371	3924898	-	chemotaxis protein CheW
	BPSL3306	3924929	3927172	-	chemotaxis two-component sensor kinase CheA
	BPSL3307	3927204	3927584	-	chemotaxis two-component response regulator CheY1
	BPSL3308	3927615	3928637	-	flagellar motor protein

	BPSL3309	3928654	3929514	-	flagellar motor protein
	BPSL3310	3929708	3930259	-	flagellar regulon master regulator subunit FlhC
	BPSL3311	3930353	3930673	-	transcriptional activator FlhD
	BPSL3318	3936905	3937117	-	30S ribosomal protein S21
	BPSL3319	3937675	3938841	+	flagellin
	BPSL3320	3938991	3940511	+	flagellar hook-associated protein
	BPSL3321	3940525	3940839	+	hypothetical protein
	BPSL3322	3941062	3943392	+	putative TPR domain protein
	BPSL3323	3943389	3944537	+	putative transferase
	BPSL3324	3944534	3945568	+	putative keto/oxo acyl-ACP synthase
	BPSL3325	3945580	3945801	+	putative acyl carrier protein
	BPSL3326	3945804	3946868	+	putative keto/oxo acyl-ACP synthase
	BPSL3327	3946865	3947629	+	putative short chain dehydrogenase
	BPSL3328	3947629	3948261	+	putative acetyltransferase
	BPSL3329	3948263	3949327	+	Rieske [2Fe-2S] domain protein
	BPSL3330	3949407	3950144	-	hypothetical protein

Table S9B: List of taurine regulated genes in Bp K96243 Chr 2 (up regulated >=2 fold)

Cluster no	GENE ID	Start	Stop	Strand	Function
1	BPSS0061	66124	67476	+	alpha-ketoglutarate permease
	BPSS0062	67529	69460	+	C4-dicarboxylate transport sensor kinase
	BPSS0063	69444	70790	+	C4-dicarboxylate transport transcriptional response regulator
2	BPSS0239	325355	325963	+	hypothetical protein
	BPSS0240	326012	326830	-	hemin ABC transport system, ATP-binding protein
	BPSS0241	327285	328406	-	hemin ABC transport system, membrane protein
	BPSS0242	328774	329700	-	hemin transport system, substrate-binding protein
	BPSS0243	329697	330848	-	hemin ABC transport system-related protein
	BPSS0244	330905	333184	-	exported heme receptor protein
3	BPSS0299	401900	403765	-	fatty-acid CoA ligase
	BPSS0300	403782	404681	-	malonyl CoA-acyl carrier protein
	BPSS0301	404682	405113	-	hypothetical protein
	BPSS0302	405181	407040	-	fatty acid biosynthesis-related CoA ligase
	BPSS0303	407070	408329	-	diaminopimelate decarboxylase
	BPSS0304	408326	409807	-	hypothetical protein
	BPSS0305	409804	410859	-	ketol-acid reductoisomerase
	BPSS0306	410865	423266	-	multifunctional polyketide-peptide syntase

	BPSS0307	423325	424737	-	aldehyde dehydrogenase
	BPSS0308	424731	426152	-	hypothetical protein
	BPSS0309	426161	427240	-	peptide synthase regulatory protein
	BPSS0310	428238	428756	-	hypothetical protein
	BPSS0311	428856	437384	-	multifunctional polyketide-peptide syntase
	BPSS0312	438602	439306	+	LuxR-family transcriptional regulator
	BPSS0313	439636	440475	+	lipoprotein
	BPSS0314	440493	441887	+	monooxygenase
	BPSS0315	441956	443098	+	ABC transport system, ATP-binding protein
	BPSS0316	443091	443747	+	ACB transport system, membrane protein
	BPSS0317	444511	445713	+	monooxygenase
	BPSS0318	445815	446642	-	hypothetical protein
4	BPSS0357	499290	499841	+	hypothetical protein
	BPSS0358	499901	500230	+	hypothetical protein
	BPSS0359	500290	501132	+	hypothetical protein
	BPSS0360	501129	502529	+	hypothetical protein
	BPSS0361	502589	502999	+	hypothetical protein
	BPSS0362	503302	503565	-	hypothetical protein
	BPSS0366	506662	507084	-	biopolymer transport protein
	BPSS0367	507104	507832	-	bipolymer transport protein
	BPSS0368	507869	508564	-	TonB-like transport protein
	BPSS0369	508840	509079	-	bacterioferritin ferredoxin protein
	BPSS0370	509211	510083	-	glutamate racemase
	BPSS0371	510130	510606	-	bacterioferritin
5	BPSS0514	698792	700396	+	acetyl-CoA hydrolase/transferase
	BPSS0515	702116	703054	+	hypothetical protein
	BPSS0516	703088	703636	+	hypothetical protein
	BPSS0517	703633	705144	+	hypothetical protein
	BPSS0518	705288	705815	+	hypothetical protein
	BPSS0519	705856	706326	+	hypothetical protein
	BPSS0520	706340	708202	+	hypothetical protein
	BPSS0521	708199	709188	+	hypothetical protein
	BPSS0522	709191	712061	+	ATPase
	BPSS0523	712052	714343	+	hypothetical protein
	BPSS0524	714509	716797	+	hypothetical protein
	BPSS0525	716801	719017	+	hypothetical protein
	BPSS0526	719014	720087	+	hypothetical protein
	BPSS0527	720090	720806	+	hypothetical protein
	BPSS0528	720849	721238	+	hypothetical protein

	BPSS0529	721244	721837	+	lipoprotein
	BPSS0530	721834	723195	+	hypothetical protein
	BPSS0531	723278	724936	+	hypothetical protein
	BPSS0532	724933	728436	+	hypothetical protein
	BPSS0533	728495	728854	+	hypothetical protein
	BPSS0534	728877	729302	+	hypothetical protein
	BPSS0535	729527	729898	+	hypothetical protein
	BPSS0536	730449	731348	+	3-hydroxydecanoyl-ACP:CoA transacylase
	BPSS0537	731570	732889	+	UDP-glucoronosyl and UDP-glucosyl transferase
	BPSS0538	732886	734472	+	transport protein
	BPSS0539	734725	735720	+	hypothetical protein
	BPSS0540	736025	737608	+	outer membrane efflux protein
	BPSS0541	738188	739354	+	HlyD family secretion protein
	BPSS0542	739867	741531	-	glycosyl hydrolase
6	BPSS0581	790031	791485	-	salicylate biosynthesis isochorismate synthase
	BPSS0582	791482	791787	-	chorismate mutase
	BPSS0583	791784	792557	-	pyochelin biosynthetic protein
	BPSS0584	792554	794197	-	salicyl-AMP ligase
	BPSS0585	794837	795778	+	AraC family regulatory protein
	BPSS0586	795905	800410	+	pyochelin synthetase
	BPSS0587	800407	806454	+	pyochelin synthetase
	BPSS0588	806451	807506	+	pyochelin biosynthetic protein
7	BPSS0993	1310767	1312284	+	catalase precursor
8	BPSS1347	1838603	1839856	-	HlyD family efflux pump protein
	BPSS1348	1840349	1841932	-	outer membrane efflux protein
	BPSS1349	1842237	1843232	-	dTDP-rhamnosyl transferase
	BPSS1350	1843461	1845047	-	efflux/sugar transport/multidrug resistance protein
	BPSS1351	1845044	1846363	-	rhamnosyltransferase protein
	BPSS1352	1846596	1847495	-	fatty acid biosynthetic protein
9	BPSS1572	2133908	2134765	-	permease component of taurine ABC transporter
	BPSS1573	2134762	2135544	-	ATP-binding component of taurine ABC transporter
	BPSS1574	2135565	2136575	-	periplasmic component of taurine ABC transporter
	BPSS1575	2136848	2137681	+	alpha-ketoglutarate-dependent taurine dioxygenase

10	BPSS1593	2167547	2169220	-	type IV pilus biosynthesis protein
	BPSS1594	2169229	2169708	-	hypothetical protein
	BPSS1595	2169748	2170302	-	major pilin subunit
	BPSS1596	2170335	2171411	-	type IV pilus biosynthesis protein
	BPSS1597	2171401	2173011	-	type IV pilus biosynthesis protein
	BPSS1598	2173008	2173511	-	hypothetical protein
	BPSS1599	2173522	2174820	-	type IV pilus biosynthesis protein
	BPSS1600	2174832	2176601	-	type IV pilus biosynthesis protein
	BPSS1601	2176601	2177170	-	type IV pilus biosynthesis protein
	BPSS1602	2177487	2178593	+	twitching motility protein
	BPSS1603	2178792	2180597	-	secretion protein
	BPSS1604	2180594	2181328	-	probable two-component system response regulator
	BPSS1605	2181394	2182479	-	probable two-component system sensor kinase
11	BPSS1631	2206192	2207145	-	hypothetical protein
	BPSS1632	2207213	2225497	-	probable non-ribosomal peptide synthetase
	BPSS1633	2225494	2238816	-	probable non-ribosomal peptide synthetase
	BPSS1634	2238813	2248688	-	probable non-ribosomal peptide synthetase
	BPSS1805	2465125	2465709	-	hypothetical protein
12	BPSS1806	2465765	2466187	-	hypothetical protein
	BPSS1807	2466225	2467268	-	4-hydroxy-2-ketovalerate aldolase
	BPSS1808	2467265	2468158	-	acetaldehyde dehydrogenase
	BPSS1809	2468155	2468997	-	thioesterase
	BPSS1810	2468994	2469938	-	branched-chain amino acid aminotransferase
	BPSS1811	2469953	2470636	-	transferase
	BPSS1812	2470644	2471441	-	non-ribosomal peptide synthesis thioesterase
	BPSS1813	2471445	2472350	-	non-ribosomal peptide synthase related protein
	BPSS1814	2472447	2472707	-	hypothetical protein
	BPSS1815	2472704	2474293	-	non-ribosomal peptide synthase
13	BPSS2214	2988112	2990238	-	catalase HPII
14	BPSS2324	3127021	3128448	-	permease
	BPSS2325	3128448	3129149	-	ABC-transporter ATP binding protein
	BPSS2326	3129149	3130636	-	flavin-binding monooxygenase-like protein
	BPSS2327	3130669	3134790	-	cytochrome P450 family protein
	BPSS2328	3134787	3141608	-	multi-domain beta keto-acyl synthase
	BPSS2329	3141712	3143346	-	acyl transferase
