# Supplementary material for: A Genomic Survey of Positive Selection in Burkholderia pseudomallei Provides Insights into the Evolution of Accidental Virulence
Source: PLoS Pathog. 2010 Apr 1;6(4):e1000845. doi: 10.1371/journal.ppat.1000845 (PMC2848565; doi:10.1371/journal.ppat.1000845)
Supplement: Text S1 — Motifs at inversions (0.06 MB PDF) [file ppat.1000845.s020.pdf]

Text S1: Motifs at inversions

We identified three large-scale inversions involving genomic regions of about 1.6Mb, 1.2Mb and 880Kb on Chromosome 1 (Figure 1 in Main Text). Bp strain 406e contained the largest inversion (~1.6Mb) which was flanked by a NnR5 protein coding gene at the 5' region and 5S_rRNAs, LSU_rRNA, tRNAs at the 3' region. The ~1.2Mb inversion in both 1655 (Australian variant) and Pasteur 52237 (Vietnam variant) was flanked by tRNAs, repeats, a transcriptional regulator, and a universal stress protein at the 5' flanking region and a 5S_rRNA and tRNA gene at the 3' region. The reference genome K96243 also has a unique inversion of ~880kb, flanked by an inverted cluster of four protein coding genes forming PAAR motifs.
