# Supplementary material for: A Genomic Survey of Positive Selection in Burkholderia pseudomallei Provides Insights into the Evolution of Accidental Virulence
Source: PLoS Pathog. 2010 Apr 1;6(4):e1000845. doi: 10.1371/journal.ppat.1000845 (PMC2848565; doi:10.1371/journal.ppat.1000845)
Supplement: Text S2 — Accuracy Estimate of RBSfinder on the B. pseudomallei genome (0.11 MB PDF) [file ppat.1000845.s021.pdf]

Text S2: Accuracy Estimate of RBSfinder on the *B. pseudomallei* genome

Previous studies have shown that the RBS motif in Bp is the same as the default consensus "AGGAG" used in the RBSfinder program [1-2]. To evaluate the accuracy of RBSfinder on the Bp genome, we compared the number of RBSs found at the start sites of previously-identified Bp K96243 genes [3] with the number of RBSs detected in a randomized DNA sequence with similar GC-content. The frequency of RBSs found in the random sequence was used as the background false positive rate.

Let RBS (real) represent the frequency of finding RBSs in a real sequence, and let RBS (random) represent the same quantity for a random sequence, then:

$$k = \frac{\text{RBS (real)} - \text{RBS (random)} \text{ X } 100}{\text{RBS (real)}}$$

where k ≈ 100% means that all (or almost all) predictions of RBSs are correct. The value k can be considered an estimate of specificity [4]. Sensitivity was estimated as the percentage of genes for which a RBS was predicted.

Sensitivity and Specificity of RBSfinder for Bp K96243 genome

	Specificity	Sensitivity
CHR1	72.4%	66%
CHR2	77.7%	70%

These sensitivity and specificity values are highly comparable to similar values reported for *E. coli* (63% specificity and 66% sensitivity, [4]). This result indicates that the RBSfinder program can be applied to identify true RBSs in the Bp genome for the novel predicted genes.

References

1. Woo PC, Leung PK, Tsoi HW, Yuen KY (2001) Cloning and characterisation of malE in *Burkholderia pseudomallei*. J Med Microbiol 50: 330-338.
2. Winstanley C, Hales BA, Hart CA (1999) Evidence for the presence in *Burkholderia pseudomallei* of a type III secretion system-associated gene cluster. J Med Microbiol 48: 649-656.
3. Holden MT, Titball RW, Peacock SJ, Cerdeno-Tarraga AM, Atkins T, et al. (2004) Genomic plasticity of the causative agent of melioidosis, *Burkholderia pseudomallei*. Proc Natl Acad Sci U S A 101: 14240-14245.
4. Suzek BE, Ermolaeva MD, Schreiber M, Salzberg SL (2001) A probabilistic method for identifying start codons in bacterial genomes. Bioinformatics 17: 1123-1130.
5. Pearson T, Giffard P, Beckstrom-Sternberg S, Auerbach R, Hornstra H, et al. (2009) Phylogeographic reconstruction of a bacterial species with high levels of lateral gene transfer. BMC Biol 7: 78.
6. Larkin MA, Blackshields G, Brown NP, Chenna R, McGettigan PA, et al. (2007) Clustal W and Clustal X version 2.0. Bioinformatics 23: 2947-2948.
7. Thompson JD, Gibson TJ, Plewniak F, Jeanmougin F, Higgins DG (1997) The CLUSTAL_X windows interface: flexible strategies for multiple sequence alignment aided by quality analysis tools. Nucleic Acids Res 25: 4876-4882.
8. Lefebure T, Stanhope MJ (2007) Evolution of the core and pan-genome of Streptococcus: positive selection, recombination, and genome composition. Genome Biol 8: R71.
9. Pritchard JK, Stephens M, Donnelly P (2000) Inference of population structure using multilocus genotype data. Genetics 155: 945-959.
10. Pundhir S, Vijayvargiya H, Kumar A (2008) PredictBias: a server for the identification of genomic and pathogenicity islands in prokaryotes. In Silico Biol 8: 223-234.
11. Sim SH, Yu Y, Lin CH, Karuturi RK, Wuthiekanun V, et al. (2008) The core and accessory genomes of *Burkholderia pseudomallei*: implications for human melioidosis. PLoS Pathog 4: e100017.
